# Supplementary material for: Evaluating large language models for ADHD education: A comparative study of ChatGPT 5, DeepSeek V3, and Grok 4
Source: PLoS One. 2026 Jun 1;21(6):e0335335. doi: 10.1371/journal.pone.0335335 (PMC13225419; doi:10.1371/journal.pone.0335335)
Supplement: S1 File — This file contains the qualitative comparative analysis tables (Tables S1–S3 in S1 File) and the complete raw responses generated by ChatGPT 5, DeepSeek V3, and Grok 4 for all three research questions (S4-S6), providing the qualitative foundation for the study’s comparative analysis. (DOCX) [file pone.0335335.s001.docx]

**Supplemental file1**

[Table S1. Comparative Analysis of the 10 Responses for (Q1: What is ADHD?) 2](#_Toc228091640)

[Table S2. Comparative Analysis of the 10 Responses for (Q2: What are its symptoms?) 8](#_Toc228091641)

[Table S3. Comparative Analysis of the 10 Responses for (Q3: What medications are used for ADHD, and do they conflict with physical exercise?) 15](#_Toc228091642)

[S4. Prompts and Generated Responses from ChatGPT 5, 08/08/2025 21](#_Toc228091643)

[S5. Prompts and Generated Responses from Grok 4, 14:28, 07/08/2025 64](#_Toc228091644)

[S6. Prompts and Generated Responses from DeepSeek V3, 15:08, 07/08/2025 121](#_Toc228091645)

# **Table S1. Comparative Analysis of the 10 Responses for (Q1: What is ADHD?)**

| **Response** | **Dimension** | **ChatGPT 5** | **DeepSeek V3** | **Grok 4** |
| --- | --- | --- | --- | --- |
| R1 | **Language Level** | Starts clear, academic tone, clear wording; gradually uses technical terms | Balanced academic tone; uses terms but keeps sentences simple, accessible for educated readers. | Popular-science tone; easy to read |
|  | **Information Depth** | Defines ADHD as a neurodevelopmental disorder, lists main features | Defines ADHD, introduces subtypes and adds causes such as genetics and brain structure. | \| Defines ADHD; symptom clusters; daily life focus \| \| --- \| |
|  | **Structural Clarity** | Well-structured: introduction, symptoms , types, Key points，Relatively long, detailed explanation | Clear sequence: definition, subtypes, causes, evaluation methods, Relatively long, detailed explanation | Intro + examples |
|  | **Additional Content** | Provided literature support | Provided literature support | Highlights daily impacts |
| R2 | **Language Level** | Starts clear, academic tone; less colloquial | Professional, clinical phrasing; uses terms like “presentation,” “environmental risk factors.” | Popular-science tone; easy to read |
|  | **Information Depth** | Adds ADHD subtypes (inattentive, hyperactive-impulsive, combined) | Explains subtypes, adds environmental risks, Treatment Options | Traits-focused; minimal causes |
|  | **Structural Clarity** | Clear logical flow: definition, types, diagnostic aspects, medium length | DSM-5 style order: definition, types, diagnostic requirements. Relatively long, detailed explanation | Simple narrative |
|  | **Additional Content** | Mention how the brain works and the genetics involved. Mention that ADHD is not limited to children. | Adds why it matters | Adds disclaimer |
| R3 | **Language Level** | Starts clear, academic tone; less colloquial | Clear and professional; language shows medical training. | Popular-science tone; easy to read |
|  | **Information Depth** | Mention of factors in the brain, as well as genetic factors. Mention of factors beyond children | Characteristics, focuses on diagnostic process: Emphasize that it can occur with other diseases ,onset before 12 years old. | Analogies; child/adult examples; lifestyle advice |
|  | **Structural Clarity** | Structured like a mini-review: intro, detail, conclusion, Relatively short | Detailed introduction to the concept, characteristics, diagnostic criteria and treatment options, Relatively long, detailed explanation | Analogy-driven, weak |
|  | **Additional Content** | Provides prevalence statistics | The global impact rate is emphasized in the concept | Lifestyle suggestions |
| R4 | **Language Level** | Starts clear, academic tone, clear wording; gradually uses technical terms | Formal, academic style; complex sentence structures. | Popular-science tone; easy to read |
|  | **Information Depth** | Strong DSM-5 framing, details diagnostic categories | Introduces dysregulation of key brain networks  proposes brain differences, core symptoms, and the probability of inheritance, and explains the paradox | Simple checklist; shallow depth |
|  | **Structural Clarity** | Stepwise, layered explanation, Relatively short | Define the term, explain the differences, core manifestations, and modern understanding, and present paradoxes, Relatively long, detailed explanation | Fragmented |
|  | **Additional Content** | Mentions co-morbidities | Increase the love paradox and the proportion of genetic factors | Disclaimer |
| R5 | **Language Level** | Accessible, popular-science style | Heavy academic phrasing; dense terminology. | Simplified, illustrative style |
|  | **Information Depth** | Explains persistence into adulthood, with moderate details | Emphasizes the definition, core characteristics, explains the ADHD brain, and presents strengths and the need for a diagnosis. It is not a character flaw. | Simplified with examples; child focus   \|  \| \| --- \| |
|  | **Structural Clarity** | Smooth narrative, not rigidly structured, Relatively short | Clear definition, characteristics, explanation, increased need for diagnosis, Relatively long, detailed explanation | Example-led |
|  | **Additional Content** | Mentions behavioural therapy | Add diagnosis requires, Not just bad behaviour | Focus on children |
| R6 | **Language Level** | Accessible, popular-science style | Academic, technical tone, minimal explanatory phrasing | Friendly, everyday   \|  \| \| --- \| |
|  | **Information Depth** | Emphasizes 3 core symptoms | Supplemented specific cases in special types and emphasized stereotypes and overlooked advantages | Focus on symptoms; practical advice |
|  | **Structural Clarity** | Clear layering, introduction, details, Relatively short | Clear definition, characteristics, stereotypes, advantages, diagnosis and treatment. Relatively long | Short narrative |
|  | **Additional Content** | Suggesting that children and adults behave differently | Attached stereotypes, sensory and sleep issues raised | Practical advice |
| R7 | **Language Level** | Accessible, popular-science style | Academic, balances professionalism with readability | Conversational |
|  | **Information Depth** | Covers the definition and mentions that ADHD is not related to laziness or poor self-control, explains how things move in the brain | balances professionalism with readability | Focus on challenges; reassurance |
|  | **Structural Clarity** | Clear, simple results that explain the content and are relatively short. | Clear and detailed, relatively long, high information density | Loose style |
|  | **Additional Content** | Additional love about the differences in physical performance between children and adults | Additional specific work-life scenarios are included to emphasize the best treatment options. | \| Reassurance \| \| --- \| |
| R8 | **Language Level** | Accessible, popular-science style | Academic, clinical register | Very simple, conversational |
|  | **Information Depth** | Clearly defined and explains how to develop. No specific everyday examples | The concepts given are specific, combined with real-life cases and characteristics, and provide diagnosis and treatment plans. | Very simplified; little detail |
|  | **Structural Clarity** | Clear, concise but accurate, of medium length | Clear, detailed explanation, relatively long, high information content | Weak clarity |
|  | **Additional Content** | The concept of ADHD as a superpower is not a limitation. | Mentioned the impact of delayed treatment | Disclaimer |
| R9 | **Language Level** | Accessible, popular-science style | Professional, slightly clinical | Popular-science, casual |
|  | **Information Depth** | School/work/social impacts; adult underdiagnosis | Focuses on differential diagnosis (e.g., vs. anxiety) and co-treatment. | Behaviour-focused; coping advice |
|  | **Structural Clarity** | Narrative, medium clarity | Clear medical writing with hierarchical logic. | Loose narrative |
|  | **Additional Content** | Mentions underdiagnosis | Adds co-treatment context. | \| Coping strategies \| \| \| --- \| --- \| \|  \| |
| R10 | **Language Level** | Accessible, popular-science style | Professional, slightly clinical, Strong academic depth; | Light conversational |
|  | **Information Depth** | \| Comprehensive: diagnosis, comorbidities, treatment, prognosis \| \| --- \| | Comprehensive: covers diagnosis, comorbidities, treatment, prognosis. | Short summary; few details |
|  | **Structural Clarity** | Relatively short, Systematic, review style | Rigid, systematic, like a medical review. | Simplified |
|  | **Additional Content** | Mentions prognosis & outcomes | Adds prognosis and notes individual variation. | Disclaimer |

# **Table S2. Comparative Analysis of the 10 Responses for (Q2: What are its symptoms?)**

| **Response** | **Dimension** | **ChatGPT 5** | **DeepSeek V3** | **Grok 4** |
| --- | --- | --- | --- | --- |
| R1 | **Language Level** | Academic but explanatory. | Balanced academic tone; uses clinical terms but explains with clear examples, | Conversational, plain words; uses everyday phrasing |
|  | **Information Depth** | Provides a full checklist of two symptom clusters， plus daily examples like forgetting homework or fidgeting. | Lists both inattentive and hyperactive-impulsive clusters; distinguishes between child and adult contexts. | Lists key inattentive and hyperactive-impulsive symptoms; depth limited, but adds school-related examples. |
|  | **Structural Clarity** | Organized into two clear clusters with bullet-like phrasing, showing diagnostic logic. | Structured in two parts with subpoints under each domain. | Organized as a straightforward checklist; minimal layering. |
|  | **Additional Content** | Refers to DSM-5 diagnostic framing, linking symptoms to official criteria. | Adds notes on how symptoms manifest differently at school vs home. | Mentions daily impacts like classroom disruption. |
| R2 | **Language Level** | More technical, showing a shift to clinical style | Professional, | Popular-science style; simple and reader-friendly. |
|  | **Information Depth** | Specifies thresholds: ≥6 symptoms for children, ≥5 for adults, lasting at least 6 months. Also explains impairment in daily life. | Specifies <12 age of onset, symptom duration (≥6 months), and need for multi-setting impairment. | Adds threshold details (≥6 child, ≥5 adult, ≥6 months), but gives fewer behavioural examples. |
|  | **Structural Clarity** | Clear and simple, medium length | Organized by diagnostic sequence: thresholds → duration → impairment. | Sequential: criteria first, then symptoms. |
|  | **Additional Content** | Notes the requirement that symptoms begin before age 12, aligning with DSM-5. | Adds comment on severity and how impairment must be “clinically significant.” | Adds disclaimer: “This is not medical advice.” |
| R3 | **Language Level** | Academic precision | Semi-clinical, clear, formal. | Simplified, analogy-driven; explains ADHD by comparing child vs adult behaviours. |
|  | **Information Depth** | Expands the inattention domain, covering organization problems, forgetfulness, and avoidance of demanding tasks. | Expands symptom lists with inattentive details (forgetfulness, poor organization) and hyperactive details (interrupting, restlessness). | Explains hyperactivity in kids vs inner restlessness in adults; adds lifestyle suggestions. |
|  | **Structural Clarity** | Itemized within one cluster, very clear and easy to follow. | Divided by two domains, each with bullet-like explanations. | Narrative and loose, not tightly structured. |
|  | **Additional Content** | Highlights functional impairment, especially academic and occupational difficulties. | Explicitly mentions DSM-5 as the source of these criteria. | Suggests managing ADHD with routine and exercise. |
| R4 | **Language Level** | Professional and clinical | Academic, precise, dense. | Conversational, brief phrasing. |
|  | **Information Depth** | Adds details on the need for symptoms to appear across multiple settings (home, school, work) and specifies exclusion rules for other disorders. | Discusses overlap of ADHD symptoms with anxiety and learning disorders; highlights the need for differential diagnosis. | Very shallow; short checklist of 2–3 symptoms only. |
|  | **Structural Clarity** | Flows logically: checklist → contexts → exclusion. | Structured as symptom list → overlap → differentiation. | Fragmented; lacks flow. |
|  | **Additional Content** | Mentions overlap with anxiety and learning disorders, highlighting diagnostic complexity. | Notes that symptom overlap can cause misdiagnosis. | Adds another disclaimer (not a substitute for diagnosis). |
| R5 | **Language Level** | More accessible | Academic but explanatory; includes phrases like “symptoms often differ between males and females.” | Simple, illustrative; compares across life stages. |
|  | **Information Depth** | Explains symptom presentation differences: hyperactivity is obvious in children but may appear as inner restlessness in adults. | Adds gender-specific discussion, noting female underdiagnosis. | Highlights difference between kids and adults; limited details on sub-symptoms. |
|  | **Structural Clarity** | Structured as child vs adult comparison, easy for readers to follow. | Structured as male vs female comparison. | Comparative narrative; child vs adult. |
|  | **Additional Content** | Notes gender differences and underdiagnosis in women. | Highlights social bias in recognition of ADHD. | Mentions gender bias in diagnosis. |
| R6 | **Language Level** | Plain and neutral; avoids heavy jargon, suitable for general readers. | Professional, clinical. | Everyday tone, friendly. |
|  | **Information Depth** | Describes developmental trajectory: hyperactivity may decline with age, but inattention often persists. Mentions comorbidities like anxiety and depression. | Focuses on symptom trajectory across lifespan; explains persistence into adulthood; adds comorbidities. | Talks about symptoms influenced by comorbidities (anxiety, depression). |
|  | **Structural Clarity** | Chronological order: childhood, adolescence, adulthood. | Chronological arrangement: childhood → adolescence → adulthood. | Narrative style, less ordered. |
|  | **Additional Content** | Adds detail about sleep problems and emotional difficulties. | Notes longitudinal studies and how symptoms evolve over decades. | Suggests self-monitoring for symptoms. |
| R7 | **Language Level** | Academic, review-like; | Clinical-academic; uses technical but clear terms. | Conversational and casual; uses simple words. |
|  | **Information Depth** | Goes beyond DSM-5 by adding executive functioning deficits and initiation problems as part of ADHD’s symptom profile. | Mentions assessment sources (parents, teachers, self-report), emphasizing triangulation. | Mentions emotional regulation and time management difficulties, not always in DSM-5. |
|  | **Structural Clarity** | Clear review logic: introduction → expanded detail → conclusion. | Structured around “who provides the information.” | Loose narrative, lacks hierarchical structure. |
|  | **Additional Content** | Mentions assessment tools and multi-informant sources (parents, teachers, self-report). | Adds importance of standardized tools (rating scales, structured interviews). | Adds informal challenges faced in daily life. |
| R8 | **Language Level** | Concise, technical phrasing, slightly drier style. | Review-like, dense, research-oriented. | Very simplified; popular-science. |
|  | **Information Depth** | Provides practical daily-life examples (losing items, missing deadlines), anchoring abstract criteria in reality. | Integrates DSM-5 criteria with functional impairment, linking symptoms to academic/occupational difficulties. | Explains misconceptions vs actual symptoms (fact-check style). |
|  | **Structural Clarity** | Structured by example within symptom categories, easy to follow. | Stepwise: criteria → examples → functional links. | Two-part layout: myths → facts. |
|  | **Additional Content** | Highlights how symptoms impair daily functioning, not just diagnostic labels. | Mentions cultural differences in symptom perception. | Adds another disclaimer (“for awareness only”). |
| R9 | **Language Level** | Professional but readable; balances formal terminology with clarity. | Academic but accessible; moderately formal. | Casual, easy to follow. |
|  | **Information Depth** | Emphasizes functional impairment across social, academic, and occupational domains. | Emphasizes functional impairment across multiple domains, with real-life illustrations. | Focuses on behavioural impacts and gives coping tips. |
|  | **Structural Clarity** | Narrative style, less structured than a checklist, but still coherent. | Narrative style, moderately structured. | Loosely structured; not diagnostic, but anecdotal. |
|  | **Additional Content** | Notes impairment as a diagnostic requirement, aligning with DSM-5. | Notes ADHD persistence into adulthood with consequences on work performance. | Adds coping strategies for stress at school or work. |
| R10 | **Language Level** | Highly professional; systematic, medical-review tone. | Highly academic, medical-review tone. | Light, conversational tone. |
|  | **Information Depth** | Comprehensive DSM-5 summary, integrating thresholds for children/adults, duration, and multi-setting requirement. | Comprehensive: integrates symptom clusters, thresholds, multi-setting requirement, comorbidities. | Very shallow; only summarizes “attention and hyperactivity problems.” |
|  | **Structural Clarity** | Review-style structure: diagnostic criteria → thresholds → conclusion. | Systematic, review-like; criteria → explanation → conclusion. | Minimal structure; one short paragraph. |
|  | **Additional Content** | Adds notes on differential diagnosis and comorbidities. | Adds discussion on differential diagnosis and prognosis. | Ends with disclaimer: “Consult a professional.” |

# **Table S3. Comparative Analysis of the 10 Responses for (Q3: What medications are used for ADHD, and do they conflict with physical exercise?)**

| **Response** | **Dimension** | **ChatGPT 5** | **DeepSeek V3** | **Grok 4** |
| --- | --- | --- | --- | --- |
| R1 | **Language Level** | Academic but clear; | Clinical-academic; | Conversational, plain; uses simple terms |
|  | **Information Depth** | Explains stimulants and non-stimulants; describes mechanisms and side effects. | Explains stimulant drugs and non-stimulants; notes their mechanisms and exercise implications. | Mentions stimulants and non-stimulants, but only briefly. Notes heart rate/blood pressure risks. |
|  | **Structural Clarity** | Structured as: drug classes → examples → mechanisms → exercise implications. | Organized by medication type → mechanism → exercise conflict. | Short checklist-like style. |
|  | **Additional Content** | Notes potential conflicts: stimulants may increase heart rate/blood pressure; advises hydration and monitoring. | Advises monitoring HR during endurance sports. | Adds tip |
| R2 | **Language Level** | Professional, more clinical tone | Highly technical | Popular-science style, friendly. |
|  | **Information Depth** | Details stimulant safety, non-stimulant tolerability, and exercise effects; mentions risk of arrhythmia under intense exertion. | Goes into detailed risks: arrhythmias, BP spikes, dehydration under stimulants; sedation, orthostatic hypotension with non-stimulants. | Lists stimulants (Ritalin, Adderall) vs non-stimulants (Strattera); briefly notes side effects. |
|  | **Structural Clarity** | Clinical-style sequencing: medication → risk → management. | Risk-focused sequence: stimulants vs non-stimulants → side effects → exercise interactions. | Clear two-part division. |
|  | **Additional Content** | Adds advice on pre-exercise medical evaluation. | Suggests ECG screening before starting stimulants in athletes. | Mentions athletes need to check anti-doping rules. |
| R3 | **Language Level** | Clear and moderately technical; | Balanced tone; technical yet explanatory. | Simplified, explanatory |
|  | **Information Depth** | Stimulants explained with focus on dopamine; non-stimulants on norepinephrine; notes both usually safe with moderate exercise. | Describes how stimulants improve focus but may increase HR/BP; non-stimulants less risky; notes that moderate aerobic exercise often enhances medication effects. | Notes stimulants boost focus but risk insomnia; exercise usually safe unless extreme. |
|  | **Structural Clarity** | Parallel structure: stimulants → non-stimulants → exercise context. | Structured as comparison with exercise outcomes. | Narrative flow, not formal. |
|  | **Additional Content** | Mentions positive effects of exercise in ADHD symptom management. | Adds point that exercise itself is therapeutic for ADHD. | Advises timing workouts earlier in the day if on stimulants. |
| R4 | **Language Level** | Professional, clinical phrasing; | Academic, uses pharmacological jargon. | Conversational, very casual. |
|  | **Information Depth** | Explains dosage adjustment strategies; stimulants can heighten cardiovascular load; non-stimulants less risky but may cause fatigue. | Details neurochemical pathways (dopamine/norepinephrine reuptake inhibition) and how these interact with exercise-induced catecholamine changes. | Very shallow—only says meds can interact with exercise “sometimes.” |
|  | **Structural Clarity** | Structured as stimulant vs non-stimulant comparison with exercise effects. | Lab-report style: mechanism → exercise interaction. | One short sentence, no structure. |
|  | **Additional Content** | Notes that individualized management is key, especially for athletes. | Adds hypothesis about synergistic effects of exercise + stimulants. | Adds disclaimer |
| R5 | **Language Level** | Accessible; uses analogy to explain stimulant effects. | Clinical, clear, | Simple, easy to read. |
|  | **Information Depth** | Explains basic drug actions without complex pharmacology; highlights exercise risks like overheating and dehydration. | Notes lifestyle considerations: stimulants may disrupt sleep, which affects exercise recovery. | Notes stimulants might increase heart strain during intense exercise; non-stimulants less risky. |
|  | **Structural Clarity** | Narrative style, less formal but clear enough. | Flow: medication → lifestyle → exercise. | Two short points (stimulant vs non-stimulant). |
|  | **Additional Content** | Advises hydration and monitoring in hot environments. | Advises avoiding late-evening stimulant use in active patients. | Suggests warm-up and cool-down. |
| R6 | **Language Level** | Balanced academic tone; avoids jargon overload. | Clinical-research tone; | Casual, explanatory. |
|  | **Information Depth** | Notes stimulants may raise heart rate; non-stimulants safer; both require supervision in strenuous sports. | Reviews evidence on long-term stimulant safety with sports; finds limited but reassuring data. | Mentions stimulants can reduce appetite and cause dehydration, which may affect sports. |
|  | **Structural Clarity** | Stepwise: drug → risk → exercise context. | Evidence-based review structure. | Narrative with cause-effect style. |
|  | **Additional Content** | Mentions role of sports physicians in ADHD management. | Notes gaps in research and need for more trials. | Recommends extra snacks or fluids before activity. |
| R7 | **Language Level** | Academic, structured; | Accessible, advisory tone. | Friendly, plain. |
|  | **Information Depth** | Discusses treatment monitoring and safety assessments; stimulant risks with endurance sports. | Stimulants can enhance alertness, but risk overheating in endurance sports; non-stimulants safer but may blunt performance. | Notes non-stimulants slower to work; stimulants fast but risk heart palpitations. |
|  | **Structural Clarity** | Organized into monitoring steps. | Comparison table-like structure: stimulants vs non-stimulants. | Parallel: stimulants vs non-stimulants. |
|  | **Additional Content** | Adds notes on tailoring exercise type (e.g., avoid ultra-marathons with stimulants). | Practical tip: adjust training load based on medication type. | Suggests avoiding high-caffeine drinks if on stimulants. |
| R8 | **Language Level** | Concise, clear, moderately clinical. | Technical but coherent. | Pop-science tone, concise. |
|  | **Information Depth** | Explains that ADHD meds and moderate exercise generally safe; highlights rare but serious risks (sudden cardiac events). | Mentions off-label drugs (bupropion, modafinil) and their effects with exercise. | Only lists one or two meds (e.g., Ritalin) and says “can affect workouts.” |
|  | **Structural Clarity** | Simple: safe generally → risks → precautions. | Category expansion: approved meds → off-label meds. | Clear problem → advice format. |
|  | **Additional Content** | Advises medical clearance before competitive sports. | Adds ethical considerations in competitive sports (doping rules). | Includes safety disclaimer. |
| R9 | **Language Level** | Clinical, precise; uses diagnostic vocabulary. | Academic and advisory mix. | Conversational, clear. |
|  | **Information Depth** | Emphasizes drug-exercise interactions; stimulants may amplify dehydration; non-stimulants can cause sedation, reducing exercise tolerance. | Stimulants improve focus but raise BP; exercise generally complementary; warns about unsupervised use. | Notes stimulants might cause overheating; exercise still encouraged with caution. |
|  | **Structural Clarity** | Divided into stimulant vs non-stimulant effects, then practical implications. | Narrative with cause-effect links. | Clear problem → advice format. |
|  | **Additional Content** | Notes importance of warm-up/cool-down to manage risks. | Adds note on individualized risk assessment. | Suggests exercising in cooler environments. |
| R10 | **Language Level** | Highly professional, review-like; systematic phrasing. | Highly professional, systematic. | Light, informal. |
|  | **Information Depth** | Integrates all key points: drug classes, examples, side effects, and exercise implications across sports types. | Integrates full scope: stimulants, non-stimulants, off-labels, lifestyle, exercise physiology. | Very shallow; only says “meds and exercise usually okay.” |
|  | **Structural Clarity** | Review structure: classification → mechanisms → exercise implications → conclusion. | Review-like: categories → mechanisms → exercise → prognosis. | One-sentence style. |
|  | **Additional Content** | Adds prognosis of ADHD with combined medication and exercise. | Adds prognosis of combined pharmacotherapy and physical training. | Ends with disclaimer: “Check with your doctor first.” |

# **S4. Prompts and Generated Responses from ChatGPT 5, 08/08/2025**

**Times 1**

1. **What is ADHD?**

ChatGPT

ADHD stands for Attention-Deficit/Hyperactivity Disorder.
It’s a neurodevelopmental condition that affects how a person pays attention, regulates impulses, and manages activity levels. It’s not about being “lazy” or “unmotivated”—it’s about differences in how the brain processes information and manages focus.

Core symptoms usually fall into three categories:

1. Inattention – Trouble sustaining attention, being easily distracted, forgetfulness, difficulty organizing tasks.
2. Hyperactivity – Restlessness, fidgeting, feeling like you’re “on the go.”
3. Impulsivity – Acting without thinking, interrupting others, difficulty waiting your turn.

ADHD can present in three types:

- Predominantly Inattentive (often what used to be called “ADD”)
- Combined Type

Key points about ADHD:

- It usually starts in childhood, but many people are not diagnosed until adulthood.
- It’s influenced by genetics, brain chemistry, and environmental factors.
- ADHD brains often have differences in dopamine and norepinephrine regulation, which affects motivation, reward processing, and attention control.
- It’s not always a disadvantage—many people with ADHD are highly creative, energetic, and innovative. The challenge is that their brains are wired differently, so standard systems (school, office work) can be harder without support.

If you’d like, I can also explain what ADHD feels like from the inside so it’s easier to imagine the day-to-day experience. Would you like me to do that?

1. **What are its symptoms?**

ChatGPT

ADHD symptoms are grouped into inattention and hyperactivity/impulsivity, and someone can have more of one group than the other—or both.

1. Inattention Symptoms

These aren’t about *never* paying attention—they’re about inconsistent focus, especially for tasks that feel boring, repetitive, or unstimulating.

- Often makes careless mistakes or overlooks details
- Trouble sustaining attention in tasks, conversations, or reading
- Seems not to listen when spoken to directly
- Struggles to follow through on instructions or finish tasks
- Disorganized—loses track of materials, deadlines, or steps in a process
- Avoids or delays tasks requiring sustained mental effort
- Frequently loses things (keys, phone, papers, etc.)
- Easily distracted by unrelated thoughts or noises
- Forgetful in daily activities (appointments, returning calls, paying bills)

2. Hyperactivity & Impulsivity Symptoms

These can be more visible, especially in kids, but they occur in adults too (sometimes in more subtle ways).

- Fidgeting, tapping hands/feet, or difficulty staying seated
- Feeling restless or “driven by a motor”
- Talking excessively or blurting out answers before questions are finished
- Trouble waiting in lines or for their turn
- Interrupting or intruding on others’ conversations or activities
- Engaging in impulsive decisions without thinking through consequences
- In adults: may manifest as internal restlessness rather than constant movement

3. Emotional & Executive Function Challenges *(not officially in the core criteria, but very common)*

- Difficulty regulating emotions (quick frustration, mood swings)
- Trouble prioritizing, starting, and finishing tasks
- Time blindness (underestimating or overestimating how long things take)
- Intense focus on things that are interesting—known as hyperfocus—while struggling to focus on less stimulating tasks

If you’d like, I can give you a side-by-side comparison of how ADHD symptoms often differ in children vs. adults—it can be surprising how it changes with age. Would you like me to show that?

1. **What medications are used for ADHD, and do they conflict with physical exercise?**

ChatGPT

ADHD medications generally fall into two main categories—stimulants and non-stimulants—and in most cases, they *don’t* conflict with physical exercise, but there are some important safety notes.

1. Stimulants (most commonly prescribed)

These increase dopamine and norepinephrine in the brain, improving focus and impulse control.

Examples:

- Methylphenidate-based
  - Ritalin, Concerta, Daytrana, Focalin
- Amphetamine-based
  - Adderall (mixed amphetamine salts), Vyvanse, Dexedrine

Exercise considerations:

- Stimulants can raise heart rate and blood pressure.
- If you’re doing high-intensity workouts, you might notice your heart rate climbs faster than usual.
- For most healthy people, this isn’t a problem, but if you have a heart condition or risk factors, your doctor might want to monitor you.
- Staying well-hydrated is important—stimulants can also mildly increase dehydration risk.

2. Non-stimulants

These are used if stimulants aren’t effective or cause too many side effects.

Examples:

- Atomoxetine (Strattera) – affects norepinephrine reuptake
- Guanfacine ER (Intuniv) – alpha-2 adrenergic agonist
- Clonidine ER (Kapvay) – alpha-2 adrenergic agonist

Exercise considerations:

- These tend to lower blood pressure or cause drowsiness, so you may feel more easily fatigued during workouts, especially early in treatment.
- Hydration and gradual warm-ups can help manage this.

3. Supplements / Adjuncts

Some people use antidepressants like bupropion (Wellbutrin) for ADHD symptoms, especially if depression is also present.
Exercise considerations: Usually no major conflict, but bupropion can slightly increase seizure risk in people with certain conditions—extreme dehydration or heat stress could make that risk more relevant.

Bottom line on ADHD meds & exercise

- Most people can exercise normally while on ADHD medication.
- The main safety concern is heart health—if you have cardiovascular disease, arrhythmias, or unexplained fainting, your doctor will likely recommend a cardiac check before starting stimulants.
- Listen to your body—if you feel dizzy, overly short of breath, or experience chest pain, stop and get checked.
- Many people actually find exercise *boosts* the effectiveness of their ADHD medication because it naturally increases dopamine and norepinephrine.

If you’d like, I can break down exactly how stimulant meds + exercise affect heart rate and performance, so you know what to expect in training. Would you like me to go there?

**Times 2**

- 1. **What is ADHD?**

ChatGPT

ADHD (Attention-Deficit/Hyperactivity Disorder) is a neurodevelopmental condition that affects how the brain regulates attention, impulses, and activity levels.

It’s not about being lazy or careless—it’s about the brain processing and prioritizing information differently, especially when it comes to focus, organization, and self-control.

Core Features

ADHD symptoms generally fall into two main categories:

1. Inattention – difficulty sustaining focus, getting easily distracted, losing things, and forgetting details.
2. Hyperactivity/Impulsivity – restlessness, excessive talking, acting without thinking, and trouble waiting turns.

A person can have:

- Predominantly inattentive type (often what used to be called ADD)
- Predominantly hyperactive-impulsive type
- Combined type (both)

How It Works in the Brain

- ADHD is linked to differences in dopamine and norepinephrine regulation, affecting motivation, reward, and executive function.
- Brain imaging shows differences in areas related to planning, impulse control, and attention management.
- It’s influenced by genetics and environmental factors—it often runs in families.

Not Just for Kids

While ADHD usually starts in childhood, many people aren’t diagnosed until adulthood. In adults, symptoms can look less like “bouncing off the walls” and more like mental restlessness, procrastination, or chronic disorganization.

If you’d like, I can also describe what living with ADHD feels like from the inside, which often paints a clearer picture than just the clinical definition.

- 1. **What are its symptoms?**

ChatGPT

ADHD symptoms fall into two main categories—inattention and hyperactivity/impulsivity—with some extra challenges that many people experience even though they aren’t in the official criteria.

1. Inattention Symptoms

(Not about never paying attention—more about *inconsistent* focus, especially on unstimulating tasks.)

- Careless mistakes or missed details
- Trouble staying focused on tasks, reading, or conversations
- Seems not to listen when spoken to directly
- Starts tasks but doesn’t finish them
- Disorganized with work, belongings, or schedules
- Avoids or delays tasks requiring sustained mental effort
- Frequently loses things like keys, wallet, or phone
- Easily distracted by sounds, sights, or unrelated thoughts
- Forgetfulness in daily activities (appointments, bills, chores)

2. Hyperactivity & Impulsivity Symptoms

(More visible in children, but can show up subtly in adults.)

- Fidgeting, tapping, or difficulty staying still
- Feeling restless or “driven by a motor”
- Talking excessively
- Blurting out answers before a question is finished
- Trouble waiting in lines or for turns
- Interrupting or intruding on others’ activities or conversations
- Impulsive decision-making without thinking through consequences

3. Common but Less-Official Challenges

- Emotional dysregulation – frustration, irritability, mood swings
- Time blindness – difficulty estimating how long things take
- Hyperfocus – intense concentration on interesting tasks, losing track of time
- Executive function struggles – difficulty prioritizing, starting, and finishing tasks

If you’d like, I can make you a side-by-side table showing how ADHD symptoms often look different in children vs. adults, since the signs change quite a bit over time.

- 1. **What medications are used for ADHD, and do they conflict with physical exercise?**

ChatGPT

ADHD treatment often includes medication, and in most cases, it doesn’t prevent you from exercising—but you should know how each type might affect your workout, especially your heart rate and energy levels.

1. Stimulants (most common)

These work by increasing dopamine and norepinephrine in the brain, which improves focus, alertness, and impulse control.

Examples:

- Methylphenidate-based: Ritalin, Concerta, Focalin, Daytrana
- Amphetamine-based: Adderall, Vyvanse, Dexedrine

Exercise notes:

- Can raise heart rate and blood pressure
- You might feel your pulse climb faster during cardio
- Slightly increase dehydration risk—drink enough water
- Usually safe if you have a healthy heart, but if you have any cardiac history, your doctor might order an ECG first

2. Non-stimulants

Used when stimulants aren’t well tolerated or aren’t effective.

Examples:

- Atomoxetine (Strattera) – affects norepinephrine
- Guanfacine ER (Intuniv) – alpha-2 adrenergic agonist
- Clonidine ER (Kapvay) – alpha-2 adrenergic agonist

Exercise notes:

- Can lower blood pressure and cause fatigue, especially early on
- May feel more tired during workouts until your body adjusts
- Warming up gradually helps

3. Other medications sometimes used for ADHD

- Bupropion (Wellbutrin) – antidepressant with dopamine/norepinephrine effects
- Usually no direct issue with exercise, but dehydration or overheating can raise seizure risk in rare cases

Key safety points

- For most people, exercise and ADHD meds are a good combination—both boost brain chemicals that help focus and mood
- Monitor heart rate, hydration, and any unusual symptoms
- Stop exercising and get checked if you experience chest pain, dizziness, or extreme shortness of breath
- If you train intensely (e.g., competitive sports), let your doctor know—they may adjust timing or dosage

If you want, I can give you a chart showing how each ADHD medication type specifically interacts with aerobic vs. strength training, so you know what to expect in each type of workout. Would you like me to make that?

**Times 3**

- 1. **What is ADHD?**

ChatGPT

ADHD (Attention-Deficit/Hyperactivity Disorder) is a neurodevelopmental condition that affects how the brain regulates attention, impulses, and activity levels.

It’s not about a lack of intelligence or willpower—it’s about differences in how the brain’s networks for focus, planning, and self-control work, especially in areas that use dopamine and norepinephrine.

Core Traits

ADHD symptoms usually fall into two main groups:

1. Inattention – trouble sustaining focus, forgetfulness, disorganization.
2. Hyperactivity/Impulsivity – restlessness, acting without thinking, interrupting.

A person can have:

- Predominantly inattentive type (often what used to be called ADD)
- Predominantly hyperactive-impulsive type
- Combined type (both)

How ADHD Works in the Brain

- Linked to differences in dopamine and norepinephrine signaling
- Affects executive functions—skills like organizing, starting tasks, staying on track, and managing time
- Often runs in families (strong genetic component)

Not Just for Kids

ADHD typically starts in childhood, but many people aren’t diagnosed until adulthood.
In adults, it may look less like “bouncing off the walls” and more like chronic procrastination, mental restlessness, or feeling overwhelmed by everyday tasks.

If you want, I can also explain what living with ADHD feels like from the inside, which can make the experience much easier to understand.

- 1. **What are its symptoms?**

ChatGPT

ADHD symptoms are grouped into two main categories—inattention and hyperactivity/impulsivity—with some extra challenges that often come along even though they aren’t part of the official diagnostic list.

1. Inattention

(This is about *inconsistent* focus, not a total inability to pay attention.)

- Overlooking details or making careless mistakes
- Trouble staying focused during tasks, reading, or conversations
- Appears not to listen when spoken to directly
- Starts tasks but struggles to finish them
- Disorganized with time, tasks, or belongings
- Avoids or delays tasks that require sustained mental effort
- Frequently loses items (keys, phone, documents, etc.)
- Easily distracted by noises, sights, or unrelated thoughts
- Forgetful in daily routines (appointments, bills, returning calls)

2. Hyperactivity & Impulsivity

(More obvious in children, often more subtle in adults.)

- Fidgeting, tapping, or difficulty staying seated
- Feeling restless or “driven by a motor”
- Talking excessively
- Blurting out answers before a question is finished
- Trouble waiting turns (in games, lines, or discussions)
- Interrupting or intruding on others’ conversations or activities
- Making quick decisions without thinking through consequences

3. Common “Unofficial” Challenges

(Not part of the core diagnostic criteria but frequently experienced.)

- Emotional dysregulation – quick frustration, mood swings
- Time blindness – underestimating or overestimating how long tasks take
- Hyperfocus – intense concentration on interesting tasks, losing track of time
- Executive function struggles – difficulty starting, prioritizing, and finishing tasks

If you want, I can make you a side-by-side chart showing how ADHD symptoms often look different in children vs. adults, since the presentation changes a lot with age.

- 1. **What medications are used for ADHD, and do they conflict with physical exercise?**

ChatGPT

ADHD medications fall into two main types—stimulants and non-stimulants—and for most people, they don’t prevent normal physical exercise.
The main thing to watch is how they affect heart rate, blood pressure, and energy levels.

1. Stimulants (most common)

Increase dopamine and norepinephrine in the brain, which improves focus and impulse control.

Examples:

- Methylphenidate-based – Ritalin, Concerta, Focalin, Daytrana
- Amphetamine-based – Adderall, Vyvanse, Dexedrine

Exercise effects:

- Can raise heart rate and blood pressure
- May make your pulse climb faster during cardio
- Mild dehydration risk—drink water before and after workouts
- Generally safe if you have a healthy heart, but a cardiac check is advised if you have heart disease or family history of sudden cardiac events

2. Non-stimulants

Used when stimulants cause side effects or aren’t effective.

Examples:

- Atomoxetine (Strattera) – norepinephrine reuptake inhibitor
- Guanfacine ER (Intuniv) – alpha-2 adrenergic agonist
- Clonidine ER (Kapvay) – alpha-2 adrenergic agonist

Exercise effects:

- Can lower blood pressure and cause fatigue, especially when starting
- Might feel sluggish during workouts until your body adjusts
- Gradual warm-ups help

3. Other medications sometimes used for ADHD

- Bupropion (Wellbutrin) – antidepressant with dopamine/norepinephrine activity
- Usually fine with exercise, but overheating or dehydration can very rarely increase seizure risk

Bottom line

- For most healthy people, ADHD meds and exercise work well together—both boost focus, mood, and energy in different ways.
- Key things to monitor:
  - Unusual shortness of breath
  - Chest pain or heart palpitations
  - Dizziness or fainting
- If you train at high intensity or compete in sports, tell your doctor—some meds are on anti-doping watch lists, and dosage timing can matter.

If you want, I can make you a quick chart showing how each ADHD medication type interacts with aerobic vs. strength training, so you know what to expect in each.

**Times 4**

1. **What is ADHD?**

ChatGPT

ADHD (Attention-Deficit/Hyperactivity Disorder) is a neurodevelopmental condition that affects how the brain manages attention, impulse control, and activity levels.

It’s not caused by laziness or a lack of intelligence—it’s about differences in brain networks that handle focus, planning, motivation, and self-regulation, especially in areas using dopamine and norepinephrine.

Main features

ADHD symptoms generally fall into two categories:

1. Inattention – distractibility, forgetfulness, trouble organizing and following through.
2. Hyperactivity/Impulsivity – restlessness, acting without thinking, interrupting.

A person can have:

- Predominantly inattentive type (often called “ADD” in the past)
- Predominantly hyperactive-impulsive type
- Combined type (both)

How ADHD works in the brain

- Linked to dopamine and norepinephrine regulation differences
- Affects executive functions—skills like prioritizing, starting tasks, staying on track, and managing time
- Often has a genetic component, and symptoms usually start in childhood

Across the lifespan

- In children, ADHD may look like constant movement, blurting out answers, and difficulty sitting still.
- In adults, it’s often more internal—mental restlessness, procrastination, time blindness, or trouble managing daily responsibilities.

If you’d like, I can explain what ADHD feels like from the inside so it’s easier to picture the day-to-day experience.

1. **What are its symptoms?**

ChatGPT

ADHD symptoms are grouped into two main categories—inattention and hyperactivity/impulsivity—plus some extra challenges that many people experience even though they’re not part of the official checklist.

1. Inattention

(This is about *inconsistent* focus, not a total inability to pay attention.)

- Overlooking details or making careless mistakes
- Trouble staying focused during tasks, reading, or conversations
- Appears not to listen when spoken to directly
- Starts tasks but has trouble finishing them
- Disorganized with schedules, tasks, or belongings
- Avoids or delays tasks that require sustained mental effort
- Frequently loses items (keys, phone, documents, etc.)
- Easily distracted by noises, sights, or unrelated thoughts
- Forgetful in daily routines (appointments, bills, chores)

2. Hyperactivity & Impulsivity

(More obvious in children; often more subtle in adults.)

- Fidgeting, tapping, or difficulty staying still
- Feeling restless or “driven by a motor”
- Talking excessively
- Blurting out answers before a question is finished
- Trouble waiting turns (in games, lines, or discussions)
- Interrupting or intruding on others’ conversations or activities
- Making quick decisions without thinking through consequences

3. Common “Unofficial” Challenges

(Not part of the formal criteria but very common for people with ADHD.)

- Emotional dysregulation – frustration, mood swings, or feeling emotions intensely
- Time blindness – poor sense of how long things take or when deadlines are approaching
- Hyperfocus – intense concentration on something interesting, losing track of time
- Executive function struggles – difficulty starting, prioritizing, and completing tasks

If you want, I can show you a side-by-side table of how ADHD symptoms differ in children vs. adults, since the way it shows up often changes a lot with age.

1. **What medications are used for ADHD, and do they conflict with physical exercise?**

ChatGPT

ADHD medications fall into two main types—stimulants and non-stimulants—and for most people, they work fine alongside physical exercise.
The main things to watch are heart rate, blood pressure, and energy levels.

1. Stimulants (most common)

Increase dopamine and norepinephrine, improving focus and impulse control.

Examples:

- Methylphenidate-based – Ritalin, Concerta, Focalin, Daytrana
- Amphetamine-based – Adderall, Vyvanse, Dexedrine

Exercise considerations:

- Can raise heart rate and blood pressure
- You may notice your pulse rises faster during cardio
- Slightly increase dehydration risk—stay well-hydrated
- Usually safe for healthy hearts, but a cardiac check is advised if you have heart disease or a family history of sudden cardiac events

2. Non-stimulants

Used when stimulants cause side effects or don’t work well.

Examples:

- Atomoxetine (Strattera) – norepinephrine reuptake inhibitor
- Guanfacine ER (Intuniv) – alpha-2 adrenergic agonist
- Clonidine ER (Kapvay) – alpha-2 adrenergic agonist

Exercise considerations:

- Can lower blood pressure and cause fatigue, especially early on
- May feel sluggish during workouts until your body adjusts
- Gentle warm-ups can help avoid dizziness

3. Other medications sometimes used

- Bupropion (Wellbutrin) – an antidepressant that also boosts dopamine/norepinephrine
- Generally fine with exercise, but overheating or dehydration can rarely raise seizure risk

Bottom line

- For most people, ADHD meds + exercise are safe and can even work well together since both improve mood, focus, and brain chemistry.
- Monitor for:
  - Chest pain, palpitations, unusual shortness of breath
  - Dizziness or fainting
- If you train at high intensity or in competitive sports, tell your doctor—some stimulants are on anti-doping watch lists, and timing your dose may matter.

If you want, I can make you a chart showing how each ADHD medication type interacts with strength vs. cardio training so you know what to expect in both settings.

**Time 5**

1. **What is ADHD?**

ChatGPT

ADHD (Attention-Deficit/Hyperactivity Disorder) is a neurodevelopmental condition that affects how the brain manages focus, impulse control, and activity levels.

It’s not about laziness or lack of intelligence—it’s caused by differences in brain networks, especially those involving dopamine and norepinephrine, which influence motivation, attention, and self-regulation.

Core traits

ADHD symptoms are grouped into:

1. Inattention – distractibility, forgetfulness, disorganization.
2. Hyperactivity/Impulsivity – restlessness, acting without thinking, interrupting.

A person can have:

- Predominantly inattentive type (often called “ADD” in the past)
- Predominantly hyperactive-impulsive type
- Combined type (both)

How it works in the brain

- Involves differences in executive functions—skills for planning, prioritizing, starting, and finishing tasks.
- Often has a genetic component and begins in childhood.
- Symptoms can change over time—children might show more hyperactivity, while adults often have more mental restlessness and organization struggles.

If you want, I can describe what ADHD feels like from the inside, which makes the experience much easier to picture.

1. **What are its symptoms?**

ChatGPT

ADHD symptoms are grouped into two main categories—inattention and hyperactivity/impulsivity—plus some common extra challenges that many people experience even though they aren’t part of the official checklist.

1. Inattention

(This is about *inconsistent* focus, not a total inability to pay attention.)

- Overlooking details or making careless mistakes
- Trouble staying focused during tasks, reading, or conversations
- Appearing not to listen when spoken to directly
- Starting tasks but struggling to finish them
- Disorganized with schedules, tasks, or belongings
- Avoiding or delaying tasks that require sustained mental effort
- Frequently losing items (keys, phone, documents, etc.)
- Easily distracted by noises, sights, or unrelated thoughts
- Forgetful in daily routines (appointments, bills, chores)

2. Hyperactivity & Impulsivity

(More obvious in children; often more subtle in adults.)

- Fidgeting, tapping, or difficulty staying still
- Feeling restless or “driven by a motor”
- Talking excessively
- Blurting out answers before a question is finished
- Trouble waiting turns (in games, lines, or discussions)
- Interrupting or intruding on others’ conversations or activities
- Making quick decisions without thinking through consequences

3. Common “Unofficial” Challenges

(Not part of the formal criteria but very common for people with ADHD.)

- Emotional dysregulation – strong mood swings or frustration
- Time blindness – poor sense of how long things take or when deadlines are close
- Hyperfocus – intense concentration on something interesting, losing track of time
- Executive function struggles – difficulty starting, prioritizing, and finishing tasks

If you’d like, I can also show you a side-by-side chart of how these symptoms look different in children vs. adults, since ADHD often changes in how it shows up over time.

1. **What medications are used for ADHD, and do they conflict with physical exercise?**

ChatGPT

ADHD medications fall into two main groups—stimulants and non-stimulants—and for most people, they don’t stop you from exercising.
The main considerations are heart rate, blood pressure, and energy levels.

1. Stimulants (most common)

Increase dopamine and norepinephrine, helping with focus, motivation, and impulse control.

Examples:

- Methylphenidate-based: Ritalin, Concerta, Focalin, Daytrana
- Amphetamine-based: Adderall, Vyvanse, Dexedrine

Exercise effects:

- Can raise heart rate and blood pressure
- May make your pulse climb faster during cardio
- Mild dehydration risk—drink water before and after workouts
- Usually fine for healthy hearts, but if you have a cardiac history or family history of sudden cardiac events, your doctor may order a heart check first

2. Non-stimulants

Used when stimulants cause too many side effects or aren’t effective.

Examples:

- Atomoxetine (Strattera) – norepinephrine reuptake inhibitor
- Guanfacine ER (Intuniv) – alpha-2 adrenergic agonist
- Clonidine ER (Kapvay) – alpha-2 adrenergic agonist

Exercise effects:

- Can lower blood pressure and cause fatigue
- You might feel more sluggish during workouts at first
- A slower warm-up can reduce dizziness

3. Other medications sometimes used

- Bupropion (Wellbutrin) – antidepressant that also boosts dopamine/norepinephrine
- Generally fine with exercise, but dehydration or overheating can *rarely* raise seizure risk

Bottom line

- For most people, ADHD meds and exercise work well together—both improve mood, focus, and brain chemistry.
- Watch for:
  - Chest pain or palpitations
  - Dizziness or fainting
  - Unusual shortness of breath
- If you train intensely or compete, let your doctor know—some stimulants are on anti-doping lists, and dose timing can matter.

If you want, I can make you a quick chart comparing how these meds affect strength training vs. cardio, so you know exactly what to expect in each type of workout.

**Times 6**

- 1. **What is ADHD?**

ChatGPT

ADHD (Attention-Deficit/Hyperactivity Disorder) is a neurodevelopmental condition that affects how the brain manages attention, impulse control, and activity levels.

It isn’t about laziness or low intelligence—it’s caused by differences in brain networks, especially those using dopamine and norepinephrine, which influence focus, motivation, planning, and self-regulation.

Core traits

ADHD symptoms are grouped into:

1. Inattention – distractibility, forgetfulness, disorganization.
2. Hyperactivity/Impulsivity – restlessness, acting without thinking, interrupting.

A person can have:

- Predominantly inattentive type (often called “ADD” in the past)
- Predominantly hyperactive-impulsive type
- Combined type (both)

How it works in the brain

- Involves differences in executive functions—skills for starting, planning, prioritizing, and completing tasks.
- Often has a genetic component and begins in childhood.
- Symptoms can shift over time—kids might show more visible hyperactivity, while adults often experience mental restlessness, procrastination, and time management struggles.

If you’d like, I can also explain what ADHD feels like from the inside, which can make it easier to imagine the day-to-day experience.

- 1. **What are its symptoms?**

ChatGPT

ADHD symptoms are grouped into two main categories—inattention and hyperactivity/impulsivity—plus some common extra challenges that many people with ADHD experience even though they aren’t in the official criteria.

1. Inattention

*(Inconsistent focus, especially for tasks that feel unstimulating)*

- Overlooking details or making careless mistakes
- Trouble staying focused during tasks, reading, or conversations
- Appearing not to listen when spoken to directly
- Starting tasks but struggling to finish them
- Disorganization with schedules, belongings, or workflow
- Avoiding or delaying tasks requiring sustained mental effort
- Frequently losing items (keys, phone, papers, etc.)
- Easily distracted by sights, sounds, or random thoughts
- Forgetfulness in daily routines (appointments, chores, bills)

2. Hyperactivity & Impulsivity

*(More obvious in children; often subtler in adults)*

- Fidgeting, tapping, or difficulty staying seated
- Feeling restless or “driven by a motor”
- Talking excessively
- Blurting out answers before a question is finished
- Trouble waiting turns in games, lines, or conversations
- Interrupting or intruding on others’ activities
- Making quick decisions without thinking through consequences

3. Common “Unofficial” Challenges

(Not in the formal DSM-5 checklist, but very common)

- Emotional dysregulation – intense frustration, mood swings, or irritability
- Time blindness – trouble judging how long tasks take or when deadlines are close
- Hyperfocus – intense concentration on something interesting, losing track of time
- Executive function struggles – difficulty starting, prioritizing, and completing tasks

If you want, I can make you a side-by-side table showing how ADHD symptoms tend to look in children vs. adults, since the signs often change with age.

- 1. **What medications are used for ADHD, and do they conflict with physical exercise?**

ChatGPT

ADHD medications fall into two main categories—stimulants and non-stimulants—and for most people they’re fine to take alongside physical exercise.
The main things to watch for are heart rate, blood pressure, hydration, and energy levels.

1. Stimulants (most common)

Increase dopamine and norepinephrine, which improve focus, motivation, and impulse control.

Examples:

- Methylphenidate-based: Ritalin, Concerta, Focalin, Daytrana
- Amphetamine-based: Adderall, Vyvanse, Dexedrine

Exercise considerations:

- Can raise heart rate and blood pressure
- Your pulse might climb faster during cardio
- Mild dehydration risk—drink water before and after workouts
- Usually fine for healthy hearts, but if you have heart disease, high blood pressure, or a family history of sudden cardiac events, a doctor may want a cardiac screening first

2. Non-stimulants

Used when stimulants aren’t effective or cause unwanted side effects.

Examples:

- Atomoxetine (Strattera) – norepinephrine reuptake inhibitor
- Guanfacine ER (Intuniv) – alpha-2 adrenergic agonist
- Clonidine ER (Kapvay) – alpha-2 adrenergic agonist

Exercise considerations:

- Can lower blood pressure and cause fatigue, especially at the start
- You might feel more sluggish during workouts until your body adapts
- A slower warm-up can reduce dizziness

3. Other medications sometimes used

- Bupropion (Wellbutrin) – an antidepressant with dopamine/norepinephrine activity
- Generally fine with exercise, but overheating or dehydration can very rarely increase seizure risk

Bottom line

- For most people, ADHD meds + exercise are safe and can actually complement each other—both improve mood and focus.
- Keep an eye out for:
  - Chest pain, palpitations, or unusual shortness of breath
  - Dizziness or fainting
- If you do high-intensity or competitive sports, let your doctor know—some stimulants are on anti-doping lists, and dosage timing might need adjusting.

If you want, I can put together a quick chart comparing how these medication types interact with cardio vs. strength training so you’ll know exactly what to expect.

**Times 7**

1. **What is ADHD?**

ChatGPT

ADHD (Attention-Deficit/Hyperactivity Disorder) is a neurodevelopmental condition that affects how the brain manages attention, impulse control, and activity levels.

It isn’t about laziness or a lack of intelligence—ADHD brains process information and regulate focus differently, especially in areas involving dopamine and norepinephrine, which are key for motivation, planning, and self-control.

Core Traits

ADHD symptoms are grouped into:

1. Inattention – distractibility, forgetfulness, disorganization.
2. Hyperactivity/Impulsivity – restlessness, acting without thinking, interrupting.

A person can have:

- Predominantly inattentive type (often called “ADD” in the past)
- Predominantly hyperactive-impulsive type
- Combined type (both)

How It Works in the Brain

- Affects executive functions—skills for planning, prioritizing, starting, and completing tasks.
- Often has a genetic component and usually begins in childhood.
- Symptoms can change over time—children often show more physical hyperactivity, while adults may experience more mental restlessness and time management struggles.

If you want, I can also explain what ADHD feels like from the inside, so it’s easier to imagine the day-to-day experience.

1. **What are its symptoms?**

ChatGPT

ADHD symptoms are grouped into two main categories—inattention and hyperactivity/impulsivity—plus some common extra challenges that many people experience even though they’re not part of the official diagnosis.

1. Inattention

*(Inconsistent focus, especially for tasks that feel boring or repetitive)*

- Overlooking details or making careless mistakes
- Trouble staying focused during work, reading, or conversations
- Appearing not to listen when spoken to directly
- Starting tasks but not finishing them
- Disorganized with schedules, belongings, or projects
- Avoiding or delaying tasks requiring sustained mental effort
- Frequently losing items (keys, phone, wallet, etc.)
- Easily distracted by noises, sights, or unrelated thoughts
- Forgetfulness in daily routines (appointments, chores, bills)

2. Hyperactivity & Impulsivity

*(More obvious in children; often more internal in adults)*

- Fidgeting, tapping, or difficulty sitting still
- Feeling restless or “driven by a motor”
- Talking excessively
- Blurting out answers before a question is finished
- Trouble waiting turns (in lines, games, or discussions)
- Interrupting or intruding on others’ conversations or activities
- Making quick decisions without thinking about consequences

3. Common “Unofficial” Challenges

(Not part of the formal DSM-5 list but very common in daily life)

- Emotional dysregulation – frustration, irritability, mood swings
- Time blindness – poor sense of how long things take or when deadlines are near
- Hyperfocus – deep concentration on something interesting, losing track of time
- Executive function struggles – difficulty starting, prioritizing, and finishing tasks

If you’d like, I can also make you a side-by-side chart showing how ADHD symptoms often differ in children vs. adults, since the way it appears changes a lot over time.

1. **What medications are used for ADHD, and do they conflict with physical exercise?**

ChatGPT

ADHD medications fall into two main groups—stimulants and non-stimulants—and in most cases they can be taken without problems during physical exercise.
The main things to watch are heart rate, blood pressure, hydration, and energy levels.

1. Stimulants (most common)

Boost dopamine and norepinephrine in the brain, which improves focus, motivation, and impulse control.

Examples:

- Methylphenidate-based: Ritalin, Concerta, Focalin, Daytrana
- Amphetamine-based: Adderall, Vyvanse, Dexedrine

Exercise considerations:

- Can raise heart rate and blood pressure
- You may notice your pulse climbs faster during cardio
- Slight dehydration risk—drink water before and after workouts
- Usually fine for healthy hearts, but if you have heart disease, high blood pressure, or a family history of sudden cardiac events, a doctor may recommend a cardiac screening first

2. Non-stimulants

Used when stimulants cause too many side effects or aren’t effective.

Examples:

- Atomoxetine (Strattera) – norepinephrine reuptake inhibitor
- Guanfacine ER (Intuniv) – alpha-2 adrenergic agonist
- Clonidine ER (Kapvay) – alpha-2 adrenergic agonist

Exercise considerations:

- Can lower blood pressure and cause fatigue, especially at the start
- Might feel more sluggish during workouts until your body adjusts
- Gentle warm-ups can help avoid dizziness

3. Other medications sometimes used

- Bupropion (Wellbutrin) – an antidepressant that also boosts dopamine/norepinephrine
- Generally fine with exercise, but in rare cases, overheating or dehydration can increase seizure risk

Bottom line

- For most people, ADHD meds and exercise are a safe and even beneficial combination—both boost brain chemicals that help focus and mood.
- Pay attention to:
  - Chest pain, palpitations, or unusual shortness of breath
  - Dizziness or fainting
- If you do intense or competitive sports, let your doctor know—some stimulants are on anti-doping watch lists, and dose timing might need adjusting.

If you want, I can create a comparison chart showing how each medication type affects cardio vs. strength training, so you’ll know exactly what to expect in each type of workout.

**Times 8**

1. **What is ADHD?**

ChatGPT

ADHD (Attention-Deficit/Hyperactivity Disorder) is a neurodevelopmental condition that affects the brain’s ability to manage attention, self-control, and activity levels.

It’s not about being lazy or careless—ADHD brains work differently, especially in areas involving dopamine and norepinephrine, the chemicals that help with motivation, planning, and regulating behavior.

Key features

ADHD symptoms fall into three patterns:

1. Inattention – distractibility, forgetfulness, disorganization
2. Hyperactivity – restlessness, fidgeting, excess energy
3. Impulsivity – acting without thinking, interrupting, impatience

A person can be:

- Predominantly inattentive type (historically called “ADD”)
- Predominantly hyperactive-impulsive type
- Combined type (both sets of symptoms)

How it develops

- Usually starts in childhood but can persist into adulthood
- Strong genetic component—runs in families
- Can affect school, work, relationships, and self-esteem if untreated

If you’d like, I can also explain what ADHD feels like day-to-day so it’s easier to picture from the inside.

1. **What are its symptoms?**

ChatGPT

ADHD symptoms are grouped into two main categories—inattention and hyperactivity/impulsivity—plus some extra challenges that many people experience even though they aren’t in the official checklist.

1. Inattention

*(Difficulty staying focused or managing tasks that feel boring or repetitive)*

- Frequently makes careless mistakes or overlooks details
- Has trouble maintaining focus during work, reading, or conversations
- Seems not to listen when spoken to directly
- Starts tasks but doesn’t finish them
- Struggles with organization (time, belongings, schedules)
- Avoids or delays tasks that require sustained mental effort
- Loses items often (keys, phone, glasses)
- Easily distracted by unrelated sights, sounds, or thoughts
- Forgets daily routines (appointments, chores, bills)

2. Hyperactivity & Impulsivity

*(More visible in children, often more subtle in adults)*

- Fidgeting, tapping, or trouble staying seated
- Feeling restless or “driven by a motor”
- Talking excessively
- Blurting out answers before a question is finished
- Struggling to wait turns (lines, games, discussions)
- Interrupting or intruding on others
- Making quick decisions without weighing the consequences

3. Common “Unofficial” Symptoms

(Not part of the formal DSM-5 criteria but common in real life)

- Emotional dysregulation – frustration, irritability, mood swings
- Time blindness – poor sense of how long things take or when deadlines are approaching
- Hyperfocus – intense, sometimes hours-long focus on interesting tasks, losing track of time
- Executive function struggles – trouble starting, prioritizing, or completing tasks

If you want, I can also make you a side-by-side chart showing how ADHD symptoms often differ in children vs. adults, since the condition can look very different at different ages.

1. **What medications are used for ADHD, and do they conflict with physical exercise?**

ChatGPT

ADHD medications fall into two main categories—stimulants and non-stimulants—and for most healthy people, they’re safe to use alongside physical exercise.
The main things to watch are heart rate, blood pressure, hydration, and how your body feels during workouts.

1. Stimulants (most common and most effective)

Increase dopamine and norepinephrine in the brain, improving focus and impulse control.

Examples:

- Methylphenidate-based: Ritalin, Concerta, Focalin, Daytrana
- Amphetamine-based: Adderall, Vyvanse, Dexedrine

Exercise considerations:

- Can raise heart rate and blood pressure
- You may feel your pulse climb faster during cardio
- Possible mild dehydration—drink water before and after workouts
- Usually safe for people without heart problems, but doctors often check heart health first, especially if you do intense training

2. Non-stimulants

Used when stimulants cause side effects or aren’t effective.

Examples:

- Atomoxetine (Strattera) – norepinephrine reuptake inhibitor
- Guanfacine ER (Intuniv) – alpha-2 adrenergic agonist
- Clonidine ER (Kapvay) – alpha-2 adrenergic agonist

Exercise considerations:

- Can lower blood pressure and cause fatigue, especially early on
- Might feel more sluggish during workouts until your body adjusts
- Warm up slowly to avoid dizziness

3. Other options sometimes prescribed

- Bupropion (Wellbutrin) – an antidepressant that also helps with dopamine/norepinephrine levels
- Generally safe with exercise, but overheating or dehydration may slightly increase seizure risk in rare cases

Bottom line

- For most people, ADHD meds and exercise are not only compatible but beneficial, since both improve mood, focus, and brain function.
- Watch for:
  - Chest pain, palpitations, or unusual shortness of breath
  - Dizziness or fainting
- If you compete in sports, check anti-doping rules—some stimulants are banned without a doctor’s exemption.

If you’d like, I can make you a chart comparing how each ADHD medication type affects cardio vs. strength training so you know exactly what to expect.

**Times 9**

1. **What is ADHD?**

ChatGPT

ADHD (Attention-Deficit/Hyperactivity Disorder) is a neurodevelopmental condition that affects the brain’s ability to regulate attention, activity level, and impulse control.

It’s caused by differences in how certain brain networks and chemicals—especially dopamine and norepinephrine—function. These differences make it harder to manage focus, organize tasks, control impulses, and regulate energy.

Core features

- Inattention – distractibility, forgetfulness, disorganization
- Hyperactivity – restlessness, excessive movement or talking
- Impulsivity – acting without thinking, interrupting, impatience

People may have:

- Predominantly inattentive presentation (often called “ADD” in the past)
- Predominantly hyperactive/impulsive presentation
- Combined presentation (both sets of symptoms)

Other important points

- ADHD usually begins in childhood but can continue into adulthood
- It’s influenced by genetics and brain development
- It’s not about laziness or a lack of intelligence—it’s about how the brain processes and regulates attention and actions

If you want, I can also explain what ADHD feels like from the inside, which can make it much easier to understand beyond the textbook definition.

1. **What are its symptoms?**

ChatGPT

ADHD symptoms are grouped into two main categories—inattention and hyperactivity/impulsivity—plus some extra traits that are common in real life but not officially in the diagnostic checklist.

1. Inattention

*(Trouble focusing, organizing, or following through on tasks)*

- Often makes careless mistakes or overlooks details
- Struggles to stay focused in work, school, or conversations
- Appears not to listen even when spoken to directly
- Starts tasks but doesn’t finish them
- Has trouble organizing time, materials, or priorities
- Avoids or delays tasks that require sustained mental effort
- Frequently loses important items (phone, keys, papers)
- Easily distracted by sights, sounds, or unrelated thoughts
- Often forgets daily responsibilities or appointments

2. Hyperactivity & Impulsivity

*(More obvious in kids, but still present in many adults)*

- Fidgets, taps, or can’t stay seated
- Feels restless or “driven by a motor”
- Talks excessively or too quickly
- Blurts out answers before a question is finished
- Struggles to wait their turn
- Interrupts or intrudes on others’ activities or conversations
- Acts without fully thinking through consequences

3. Common “Unofficial” Symptoms

(Not part of the DSM-5 criteria, but often reported)

- Emotional regulation issues – frustration, irritability, mood swings
- Time blindness – poor sense of how long things take or when deadlines are close
- Hyperfocus – intense, tunnel-vision concentration on something interesting, losing track of time
- Executive function challenges – trouble starting, planning, prioritizing, or finishing tasks

If you’d like, I can show you how ADHD symptoms often look different in children vs. adults, since the presentation changes over time.

1. **What medications are used for ADHD, and do they conflict with physical exercise?**

ChatGPT

ADHD medications fall into two main groups—stimulants and non-stimulants—and for most healthy people, they can be safely combined with physical exercise.
The main thing to watch is how they affect heart rate, blood pressure, hydration, and workout intensity.

1. Stimulants

*(Most commonly prescribed and usually the most effective)*
Increase dopamine and norepinephrine in the brain, improving focus and self-control.

Examples:

- Methylphenidate-based: Ritalin, Concerta, Focalin, Daytrana
- Amphetamine-based: Adderall, Vyvanse, Dexedrine

Exercise considerations:

- Can raise heart rate and blood pressure
- You may feel your pulse climb faster during cardio
- May slightly increase risk of overheating, so hydrate well
- Safe for most people without heart disease, but intense athletes may get an ECG check first

2. Non-Stimulants

*(Used when stimulants cause side effects or aren’t effective)*

Examples:

- Atomoxetine (Strattera) – norepinephrine reuptake inhibitor
- Guanfacine ER (Intuniv) – alpha-2 adrenergic agonist
- Clonidine ER (Kapvay) – alpha-2 adrenergic agonist

Exercise considerations:

- Some can lower blood pressure, causing lightheadedness when standing or during intense workouts
- Fatigue may be more noticeable early in treatment
- Warm up gradually to avoid dizziness

3. Other medications sometimes used

- Bupropion (Wellbutrin) – antidepressant that also boosts dopamine and norepinephrine
- Generally safe for exercise, but overheating and dehydration can slightly raise seizure risk in rare cases

Bottom line

- For most people, ADHD meds and exercise work well together—both improve mood, focus, and overall brain health.
- Red flags to stop and check with a doctor:
  - Chest pain, palpitations, or shortness of breath
  - Dizziness or fainting
- If you compete in sports, check anti-doping rules—many stimulants require a doctor’s medical exemption.

If you want, I can make you a table showing each ADHD medication type and its specific exercise effects so it’s easier to see at a glance.

**Times 10**

1. **What is ADHD?**

ChatGPT

ADHD (Attention-Deficit/Hyperactivity Disorder) is a neurodevelopmental condition that affects the brain’s ability to regulate attention, activity levels, and impulse control.

It’s linked to differences in how certain brain networks work—especially those involving dopamine and norepinephrine—which help manage focus, motivation, and self-regulation.

Core characteristics

- Inattention – easily distracted, forgetful, disorganized
- Hyperactivity – restless, fidgety, “on the go” feeling
- Impulsivity – acting or speaking without thinking through consequences

People may have:

- Predominantly inattentive type (formerly called “ADD”)
- Predominantly hyperactive-impulsive type
- Combined type (a mix of both)

Other key points

- Usually starts in childhood, but many continue to experience it into adulthood
- Strong genetic component—tends to run in families
- Not caused by laziness, low intelligence, or poor parenting—it’s about how the brain is wired
- Can be managed through a mix of strategies: medication, therapy, lifestyle adjustments, and support systems

If you’d like, I can also explain what living with ADHD feels like from the inside, which often makes it easier to truly understand.

1. **What are its symptoms?**

ChatGPT

ADHD symptoms fall into two main categories—inattention and hyperactivity/impulsivity—plus some extra traits that aren’t officially in the diagnosis but are common in daily life.

1. Inattention

*(Difficulty sustaining focus, organizing, or following through on tasks)*

- Makes careless mistakes, misses details
- Has trouble staying focused in work, school, or conversations
- Seems not to listen when spoken to directly
- Starts tasks but often leaves them unfinished
- Disorganized with time, materials, or priorities
- Avoids or delays tasks that require long mental effort
- Frequently loses important items (phone, keys, documents)
- Easily distracted by outside stimuli or unrelated thoughts
- Often forgets to do routine tasks or keep appointments

2. Hyperactivity & Impulsivity

*(More visible in children, but still present in adults)*

- Fidgets, taps hands/feet, or can’t stay seated
- Feels restless or “driven by a motor”
- Talks excessively or too fast
- Blurts out answers before questions are finished
- Has difficulty waiting their turn
- Interrupts or intrudes on other people’s activities
- Acts without thinking through risks or consequences

3. Common “Unofficial” Symptoms

(Not in the official DSM-5 criteria but widely reported)

- Emotional swings – frustration, irritability, quick mood changes
- Time blindness – poor sense of time passing or how long tasks take
- Hyperfocus – intense concentration on something interesting, losing track of time
- Executive function struggles – difficulty planning, prioritizing, starting, or finishing projects

If you’d like, I can also break down how ADHD symptoms show up differently in children vs. adults, since the signs often shift with age.

1. **What medications are used for ADHD, and do they conflict with physical exercise?**

ChatGPT

ADHD medications fall into two main categories—stimulants and non-stimulants—and for most healthy people, they can be taken safely alongside regular physical exercise.
The main concern is how they affect heart rate, blood pressure, body temperature, and hydration during activity.

1. Stimulants

*(Most prescribed, usually most effective)*
They boost dopamine and norepinephrine in the brain, improving focus and self-control.

Examples:

- Methylphenidate-based: Ritalin, Concerta, Focalin, Daytrana
- Amphetamine-based: Adderall, Vyvanse, Dexedrine

Exercise notes:

- Can raise heart rate and blood pressure, especially during intense cardio
- May slightly increase body temperature and dehydration risk
- Safe for most people without underlying heart problems
- Athletes in competitive sports may need a medical exemption because stimulants are often on anti-doping lists

2. Non-Stimulants

*(Used when stimulants cause side effects or aren’t effective)*

Examples:

- Atomoxetine (Strattera) – norepinephrine reuptake inhibitor
- Guanfacine ER (Intuniv) – alpha-2 adrenergic agonist
- Clonidine ER (Kapvay) – alpha-2 adrenergic agonist

Exercise notes:

- Some can lower blood pressure, which may cause dizziness or lightheadedness when standing quickly or during high-intensity workouts
- Fatigue is more common early in treatment, so warming up gradually is wise

3. Other options

- Bupropion (Wellbutrin) – antidepressant sometimes used off-label
- Generally safe for exercise, but overheating and dehydration can slightly increase seizure risk in rare cases

Bottom line

- For most healthy individuals, ADHD meds and exercise go well together—in fact, both can help boost focus, mood, and energy regulation.
- Red flags to stop and check with a doctor:
  - Chest pain, irregular heartbeat, shortness of breath
  - Dizziness or fainting during workouts
- Always discuss with your doctor before starting an intense new fitness routine while on ADHD meds, especially if you have heart issues.

If you want, I can make you a simple chart comparing each ADHD medication type with its possible exercise effects so it’s easier to see at a glance.

# **S5. Prompts and Generated Responses from Grok 4, 14:28, 07/08/2025**

**Times 1**

- 1. **What is ADHD?**

ADHD, or Attention-Deficit/Hyperactivity Disorder, is a neurodevelopmental condition characterized by persistent challenges with attention, impulse control, and/or hyperactivity that are inappropriate for a person's age. It typically emerges in childhood and can continue into adulthood, affecting daily functioning in areas like school, work, or relationships. Key Features:

Inattention: Difficulty sustaining focus, organizing tasks, or following through on instructions. People might seem forgetful, easily distracted, or struggle with details. Hyperactivity: Excessive movement or restlessness, such as fidgeting, talking excessively, or being unable to stay seated when expected. Impulsivity: Acting without thinking, interrupting others, or making hasty decisions.

Types of ADHD:

Predominantly Inattentive Presentation: Primarily struggles with attention and organization. Predominantly Hyperactive-Impulsive Presentation: Mainly shows hyperactivity and impulsive behaviours. Combined Presentation: A mix of both inattention and hyperactivity-impulsivity.

Causes: The exact cause isn’t fully understood, but factors include:

Genetics: ADHD often runs in families. Brain Structure/Function: Differences in brain areas related to attention and impulse control. Environmental Factors: Exposure to toxins, premature birth, or maternal substance use during pregnancy may contribute.

Diagnosis:

Diagnosed by professionals (e.g., psychologists, psychiatrists) using criteria from the DSM-5. Involves assessing symptoms, their duration (at least 6 months), and impact across settings (e.g., home, school). Symptoms must be present before age 12 and not better explained by other conditions.

Treatment:

Behavioural Therapy: Teaches coping strategies and organizational skills. Medication: Stimulants (e.g., methylphenidate, amphetamines) or non-stimulants to improve focus and impulse control. Lifestyle Changes: Regular exercise, structured routines, and dietary adjustments can help.

Prevalence:

Affects about 5-10% of children and 2-5% of adults globally. More commonly diagnosed in males, though females may be underdiagnosed due to subtler symptoms.

ADHD varies widely in severity and presentation, and with proper support, many individuals lead successful, fulfilling lives. If you want specifics on symptoms, treatments, or personal strategies, let me know!

- 1. **What are its symptoms?**

ADHD symptoms vary by individual and can differ based on the type of ADHD (inattentive, hyperactive-impulsive, or combined). Below is a breakdown of the primary symptoms, grouped by category, based on the DSM-5 criteria used for diagnosis. These symptoms must be persistent (lasting at least 6 months), inappropriate for the person’s age, and present in multiple settings (e.g., home, school, work). Inattention Symptoms These are more common in the Predominantly Inattentive Presentation but can appear in the combined type:

Difficulty sustaining attention: Struggling to focus on tasks or activities, such as schoolwork, conversations, or reading. Easily distracted: Frequently sidetracked by external stimuli (e.g., noises) or unrelated thoughts. Poor organization: Trouble planning or completing tasks, managing time, or keeping track of belongings (e.g., losing keys, homework). Forgetfulness: Forgetting daily tasks, appointments, or instructions. Avoiding tasks requiring sustained effort: Procrastinating or avoiding activities like studying or paperwork that demand prolonged mental focus. Frequent careless mistakes: Overlooking details in work or activities due to lack of attention. Difficulty following instructions: Starting tasks but failing to complete them or missing steps. Losing focus in conversations: Not listening when spoken to or drifting off during discussions.

Hyperactivity-Impulsivity Symptoms These are more common in the Predominantly Hyperactive-Impulsive Presentation but can appear in the combined type:

Fidgeting or restlessness: Constantly moving, tapping hands/feet, or squirming in seats. Inability to stay seated: Leaving seats in situations where remaining seated is expected (e.g., in class or meetings). Excessive talking: Talking too much or out of turn, often dominating conversations. Running or climbing inappropriately: In children, this might mean excessive physical activity in unsuitable settings; in adults, it may manifest as feeling “driven by a motor” or inability to relax. Difficulty engaging quietly: Struggling to do activities like reading or playing without making noise. Impulsivity: Acting without thinking, such as blurting out answers, interrupting others, or making snap decisions. Trouble waiting for a turn: Impatience in lines, games, or conversations. Intruding on others: Taking over activities or conversations without permission or consideration.

Key Notes:

Age and Context: Symptoms must be inappropriate for the person’s developmental stage. For example, hyperactivity in a 5-year-old might look like constant running, while in an adult, it might feel like inner restlessness or excessive multitasking. Children vs. Adults: In children, hyperactivity is often more physical; in adults, it may appear as fidgeting or feeling overwhelmed by responsibilities. Inattentive symptoms in adults might include chronic disorganization or difficulty meeting deadlines. Diagnosis Threshold: For a diagnosis, children must show at least 6 symptoms (from either or both categories) before age 12, while adults need at least 5, with significant impact on daily life. Co-occurring Conditions: Symptoms may overlap with anxiety, depression, or learning disabilities, complicating diagnosis.

Examples in Daily Life:

A child with ADHD might forget homework, interrupt teachers, or struggle to sit through class. An adult might miss deadlines, lose personal items frequently, or act impulsively in social or financial decisions.

If you’re curious about how these symptoms might look in a specific context (e.g., school, workplace, or relationships) or want details on managing them, let me know! Disclaimer: Grok is not a doctor; please consult one. Don't share information that can identify you.

- 1. **What medications are used for ADHD, and do they conflict with physical exercise?**

Medications Used for ADHD Medications for ADHD primarily aim to improve focus, reduce impulsivity, and manage hyperactivity by targeting brain neurotransmitters like dopamine and norepinephrine. Below are the main types of medications used, based on current medical standards: 1. Stimulants These are the most commonly prescribed and effective for ADHD, working by increasing dopamine and norepinephrine levels in the brain.

Methylphenidate-based:

Examples: Ritalin, Concerta, Methylin, Daytrana (patch), Aptensio, Focalin (dexmethylphenidate). Forms: Short-acting (3-5 hours), intermediate-acting (6-8 hours), or long-acting (8-12+ hours).

Amphetamine-based:

Examples: Adderall, Vyvanse, Dexedrine, Evekeo. Forms: Short-acting, extended-release, or prodrug (e.g., Vyvanse, which is converted to dextroamphetamine in the body).

Effects: Improve attention, reduce impulsivity, and calm hyperactivity in about 70-80% of patients. Side Effects: Appetite suppression, insomnia, increased heart rate, anxiety, or headaches. Rare but serious risks include heart complications or psychiatric effects (e.g., mood swings).

2. Non-Stimulants These are used when stimulants are ineffective, cause intolerable side effects, or are not suitable (e.g., due to substance abuse history).

Atomoxetine (Strattera):

A norepinephrine reuptake inhibitor, often used for inattentive ADHD or when anxiety is present. Effects: Takes 2-6 weeks to show benefits, less immediate than stimulants. Side Effects: Fatigue, nausea, mood swings, or rare liver issues.

Guanfacine (Intuniv, Tenex) and Clonidine (Kapvay):

Alpha-2 agonists, often used for hyperactivity or impulsivity, especially in children or as an adjunct to stimulants. Effects: Can help with emotional regulation and sleep. Side Effects: Drowsiness, low blood pressure, or dizziness.

Bupropion (Wellbutrin):

An antidepressant sometimes used off-label for ADHD, particularly with co-occurring depression. Side Effects: Insomnia, dry mouth, or risk of seizures at high doses.

3. Other Medications

Antidepressants: Tricyclic antidepressants (e.g., desipramine) or SSRIs may be used off-label for ADHD with co-occurring conditions like depression or anxiety. Modafinil: Occasionally used off-label for ADHD, though not FDA-approved for this purpose. Combination Therapies: Doctors may combine medications (e.g., a stimulant with guanfacine) to target specific symptoms or manage side effects.

Key Notes on Medications:

Individual Response: Effectiveness and side effects vary widely. Finding the right medication and dose often requires trial and error. Administration: Stimulants come in various forms (pills, capsules, patches, liquids) with different durations to suit daily schedules. Monitoring: Regular follow-ups with a healthcare provider are crucial to adjust doses and monitor side effects, especially for heart health or growth in children.

Do ADHD Medications Conflict with Physical Exercise? Generally, ADHD medications do not conflict with physical exercise and can often complement it, as exercise itself is a beneficial non-pharmacological strategy for managing ADHD symptoms. However, there are considerations to ensure safety and optimize benefits: 1. Stimulants and Exercise

Potential Concerns:

Cardiovascular Effects: Stimulants can increase heart rate and blood pressure, which may be amplified during intense exercise. This is rarely an issue for healthy individuals but requires caution in those with pre-existing heart conditions (e.g., arrhythmias, hypertension). Dehydration: Stimulants may reduce thirst perception, increasing dehydration risk during exercise, especially in hot environments. Appetite Suppression: Reduced appetite can lead to low energy for exercise if nutrition isn’t managed.

Precautions:

Get a medical evaluation before starting stimulants, especially if engaging in vigorous exercise or competitive sports. An ECG may be recommended for those with heart risk factors. Stay hydrated and maintain a balanced diet to support energy needs. Avoid exercising at peak medication times if side effects like elevated heart rate feel uncomfortable.

Benefits: Stimulants can enhance focus during exercise, which may improve performance in structured activities like sports or workouts. Exercise itself boosts dopamine, potentially enhancing medication effects.

2. Non-Stimulants and Exercise

Atomoxetine:

Concerns: May cause fatigue or dizziness initially, which could affect exercise performance. These effects often diminish over time. Precautions: Start exercise gradually and monitor for light-headedness, especially when initiating treatment. Benefits: Less impact on heart rate than stimulants, making it safer for some during intense physical activity.

Guanfacine/Clonidine:

Concerns: Can cause sedation or lower blood pressure, potentially leading to fatigue or dizziness during exercise. Precautions: Schedule exercise when sedation is minimal (e.g., morning if taken at night). Monitor for low blood pressure symptoms (e.g., fainting). Benefits: May help with emotional regulation during team sports or high-pressure activities.

Bupropion:

Concerns: Risk of seizures at high doses, particularly with intense exercise or dehydration. Precautions: Ensure proper hydration and avoid extreme exertion without medical guidance.

General Benefits of Exercise for ADHD:

Symptom Management: Exercise increases dopamine and norepinephrine, mimicking some medication effects, improving focus and mood. Complementary Effects: Regular physical activity (e.g., aerobic exercise, yoga) can reduce hyperactivity, enhance impulse control, and improve sleep, potentially reducing reliance on medication. Types of Exercise: Aerobic activities (running, swimming), martial arts, or team sports are particularly effective for ADHD due to their structure and engagement.

Practical Tips:

Timing: Schedule exercise when medication side effects (e.g., sedation from guanfacine or peak stimulant effects) don’t interfere. For stimulants, this might be later in the day for short-acting forms. Monitor Heart Rate: Use a fitness tracker to ensure heart rate stays within safe limits, especially with stimulants. Nutrition and Hydration: Counter appetite suppression with nutrient-dense meals/snacks before exercise and maintain hydration. Consult a Doctor: If starting a new exercise regimen or medication, discuss potential interactions, especially for competitive athletes or those with health conditions.

Summary ADHD medications, particularly stimulants and non-stimulants, are generally safe with physical exercise for most individuals, but precautions are needed to manage cardiovascular effects, hydration, and energy levels. Exercise can enhance ADHD treatment by improving symptoms and overall well-being. Always consult a healthcare provider to tailor medication and exercise plans, especially if you have underlying health conditions or are starting a new regimen. If you want details on specific medications, exercise routines for ADHD, or managing side effects, let me know! Disclaimer: Grok is not a doctor; please consult one. Don't share information that can identify you.

**Times 2**

- 1. **What is ADHD?**

ADHD, or Attention-Deficit/Hyperactivity Disorder, is a neurodevelopmental condition characterized by persistent patterns of inattention, impulsivity, and/or hyperactivity that are inappropriate for a person’s age and interfere with daily functioning. It typically emerges in childhood and can continue into adulthood. Symptoms are grouped into three main types:

Inattentive Type: Difficulty sustaining attention, organizing tasks, or following through on instructions; easily distracted; forgetful in daily activities. Hyperactive-Impulsive Type: Excessive fidgeting, restlessness, interrupting others, difficulty waiting for one’s turn, or acting without thinking. Combined Type: A mix of inattentive and hyperactive-impulsive symptoms.

The exact cause isn’t fully understood, but genetics, brain structure differences, and environmental factors (like prenatal exposure to toxins) are thought to play roles. Diagnosis is based on clinical criteria, often using tools like the DSM-5, and involves assessing behaviour across multiple settings (e.g., home, school). Treatment typically includes behavioural therapy, medication (like stimulants or non-stimulants), and lifestyle adjustments. ADHD affects about 5-7% of children and 2-5% of adults globally, with males diagnosed more often than females. If you want more details, like specific diagnostic criteria or treatment options, let me know!

- 1. **What are its symptoms?**

I’m assuming you’re referring to ADHD again, as we were just discussing it. If you meant something else, please clarify! Below is a concise list of ADHD symptoms, categorized by the two main types (inattention and hyperactivity-impulsivity), based on DSM-5 criteria. These must be persistent, age-inappropriate, and impact multiple settings (e.g., home, school, work).

**Inattention Symptoms**

- Difficulty sustaining focus on tasks or conversations.
- Easily distracted by external stimuli or unrelated thoughts.
- Poor organization, losing items, or forgetting daily tasks.
- Avoiding tasks requiring sustained mental effort (e.g., studying, paperwork).
- Making careless mistakes or failing to follow instructions.

**Hyperactivity-Impulsivity Symptoms**

- Fidgeting, squirming, or feeling restless (in adults, often internal restlessness).
- Inability to stay seated when expected (e.g., in class or meetings).
- Excessive talking or interrupting others.
- Acting impulsively, such as blurting out answers or making hasty decisions.
- Difficulty waiting for a turn or intruding on others’ activities.

**Key Points**

- **Children**: Need 6+ symptoms from either/both categories, starting before age 12.
- **Adults**: Need 5+ symptoms, often presenting as disorganization or restlessness rather than overt hyperactivity.
- **Impact**: Symptoms must disrupt daily life (e.g., school, work, relationships).
- **Variations**: Symptoms differ by ADHD type (inattentive, hyperactive-impulsive, or combined) and may overlap with conditions like anxiety.

If you want examples of how these symptoms show up in specific contexts (e.g., kids vs. adults, workplace vs. home) or more details, let me know!

*Disclaimer: Grok is not a doctor; please consult one. Don't share information that can identify you.*

- 1. **What medications are used for ADHD, and do they conflict with physical exercise?**

I notice you previously asked about ADHD medications and their interaction with physical exercise. Since you’re asking again, I’ll provide a concise, updated answer to ensure clarity and address any potential nuances, while avoiding unnecessary repetition. If you’re looking for specific details (e.g., a particular medication, exercise type, or new angle), please let me know!

**Medications Used for ADHD**

ADHD medications primarily target dopamine and norepinephrine to improve focus, reduce impulsivity, and manage hyperactivity. Here’s a summary of the main types:

**1. Stimulants (First-line, ~70-80% effective)**

- **Methylphenidate-based**: Ritalin, Concerta, Focalin, Daytrana (patch).
- **Amphetamine-based**: Adderall, Vyvanse, Dexedrine.
- **Forms**: Short-acting (3-5 hours), intermediate (6-8 hours), or long-acting (8-12+ hours).
- **Side Effects**: Appetite loss, insomnia, increased heart rate, anxiety, headaches. Rare risks: heart issues, mood changes.

**2. Non-Stimulants (Used if stimulants are unsuitable)**

- **Atomoxetine (Strattera)**: Norepinephrine reuptake inhibitor, takes 2-6 weeks to work. Side effects: fatigue, nausea, rare liver issues.
- **Guanfacine (Intuniv)** and **Clonidine (Kapvay)**: Alpha-2 agonists, help with hyperactivity and emotional regulation. Side effects: sedation, low blood pressure.
- **Bupropion (Wellbutrin)**: Off-label, used for ADHD with depression. Side effects: insomnia, seizure risk at high doses.

**3. Other:**

- Rarely, tricyclic antidepressants or modafinil are used off-label.
- Combinations (e.g., stimulant + guanfacine) may target specific symptoms.

**Note**: Medication choice depends on individual response, side effects, and co-occurring conditions. Regular medical monitoring is essential.

**Do ADHD Medications Conflict with Physical Exercise?**

ADHD medications generally **do not conflict with physical exercise** and can complement it, as exercise boosts dopamine and helps manage ADHD symptoms. However, considerations apply:

**Stimulants and Exercise**

- **Concerns**:
  - Increased heart rate/blood pressure from stimulants may amplify during intense exercise, posing risks for those with heart conditions.
  - Appetite suppression can reduce energy for workouts if nutrition is neglected.
  - Reduced thirst perception may increase dehydration risk.
- **Precautions**:
  - Get a cardiac evaluation before combining stimulants with vigorous exercise, especially for competitive sports.
  - Stay hydrated and eat nutrient-dense meals/snacks before exercising.
  - Time exercise to avoid peak medication effects if heart rate feels too high.
- **Benefits**: Improved focus from stimulants may enhance performance in structured activities (e.g., sports).

**Non-Stimulants and Exercise**

- **Atomoxetine**:
  - **Concerns**: Initial fatigue or dizziness may affect exercise stamina.
  - **Precautions**: Start exercise gradually; monitor for lightheadedness.
  - **Benefits**: Minimal cardiovascular impact compared to stimulants.
- **Guanfacine/Clonidine**:
  - **Concerns**: Sedation or low blood pressure may cause fatigue or dizziness during exercise.
  - **Precautions**: Schedule exercise when sedation is low (e.g., morning for nighttime doses).
  - **Benefits**: Can aid emotional control in team sports.
- **Bupropion**:
  - **Concerns**: Seizure risk with high doses, especially if dehydrated.
  - **Precautions**: Ensure hydration and avoid extreme exertion.

**General Benefits of Exercise:**

- Boosts dopamine/norepinephrine, enhancing medication effects.
- Reduces hyperactivity, improves focus, and aids sleep.
- Aerobic exercise, martial arts, or team sports are especially effective.

**Practical Tips:**

- **Timing**: Exercise when medication side effects (e.g., sedation, peak stimulant effects) are minimal.
- **Monitoring**: Use a fitness tracker to check heart rate, especially with stimulants.
- **Nutrition/Hydration**: Counter appetite suppression with balanced meals and ensure adequate water intake.
- **Medical Guidance**: Consult a doctor to align medication and exercise plans, particularly for intense activities or health conditions.

**Summary**

ADHD medications (stimulants and non-stimulants) are generally safe with exercise, but precautions are needed for cardiovascular effects, hydration, and energy levels. Exercise can enhance ADHD management. Always work with a healthcare provider to tailor plans.

If you want specifics (e.g., a particular medication’s interaction with a sport, managing side effects, or exercise recommendations), let me know!

*Disclaimer: Grok is not a doctor; please consult one. Don't share information that can identify you.*

**Times 3**

- 1. **What is ADHD?**

ADHD, or Attention-Deficit/Hyperactivity Disorder, is a neurodevelopmental condition characterized by persistent patterns of inattention, impulsivity, and/or hyperactivity that are inappropriate for a person's age and interfere with daily functioning. It typically begins in childhood and can continue into adulthood, affecting areas like school, work, or relationships.

**Key Features:**

1. **Inattention**: Difficulty focusing, staying organized, or completing tasks. Examples include forgetting daily activities, losing things, or being easily distracted.
2. **Hyperactivity**: Excessive movement or restlessness, such as fidgeting, inability to sit still, or talking excessively.
3. **Impulsivity**: Acting without thinking, interrupting others, or struggling to wait for one's turn.

**Types of ADHD:**

- **Predominantly Inattentive Presentation**: Mostly struggles with attention and organization.
- **Predominantly Hyperactive-Impulsive Presentation**: Primarily shows hyperactivity and impulsive behaviors.
- **Combined Presentation**: A mix of both inattention and hyperactivity-impulsivity.

**Causes:**

The exact cause is unknown, but factors include:

- Genetics (strong hereditary component).
- Brain structure differences, particularly in areas regulating attention and impulse control.
- Environmental factors, like exposure to toxins or prenatal substance use.

**Diagnosis:**

Diagnosed by professionals (e.g., psychologists, psychiatrists) using criteria from the DSM-5, based on behavior patterns observed over time. It often involves input from parents, teachers, or others.

**Treatment:**

- **Medication**: Stimulants (e.g., methylphenidate, amphetamines) or non-stimulants to manage symptoms.
- **Therapy**: Behavioral therapy, cognitive-behavioral therapy (CBT), or coaching to build coping strategies.
- **Lifestyle**: Structured routines, exercise, and diet can help manage symptoms.

ADHD affects about 5-7% of children and 2-5% of adults globally. It’s more commonly diagnosed in males, though females often present with inattentive symptoms, which can be underdiagnosed.

If you’d like, I can search for recent discussions or research on ADHD or provide tips for managing it!

*Disclaimer: Grok is not a doctor; please consult one. Don't share information that can identify you.*

- 1. **What are its symptoms?**

ADHD symptoms vary by individual and can differ based on the type of ADHD (inattentive, hyperactive-impulsive, or combined). Below is a breakdown of common symptoms, based on the DSM-5 criteria and general understanding of the condition:

**Inattention Symptoms**

These are more prominent in the inattentive presentation but can appear in the combined type:

- Difficulty sustaining attention in tasks or play.
- Frequently making careless mistakes in schoolwork, work, or other activities.
- Trouble organizing tasks or activities (e.g., messy workspaces, poor time management).
- Easily distracted by unrelated thoughts or external stimuli.
- Forgetfulness in daily activities (e.g., forgetting appointments, losing items like keys or homework).
- Avoiding or disliking tasks requiring sustained mental effort (e.g., homework, reports).
- Frequently losing things necessary for tasks (e.g., books, tools, phone).
- Difficulty following through on instructions or completing tasks.
- Appearing not to listen when spoken to directly.

**Hyperactivity-Impulsivity Symptoms**

These are more common in the hyperactive-impulsive presentation but can also appear in the combined type:

- Fidgeting, tapping hands/feet, or squirming in seat.
- Inability to stay seated in situations where it’s expected (e.g., classroom, meetings).
- Running or climbing in inappropriate situations (in children; in adults, this may manifest as restlessness).
- Difficulty engaging in quiet activities.
- Excessive talking or blurting out answers before questions are completed.
- Trouble waiting for one’s turn (e.g., in conversations, lines, or games).
- Interrupting or intruding on others’ conversations or activities.
- Acting without thinking about consequences (e.g., impulsive decisions).

**Key Notes:**

- **Age and Context**: Symptoms must be inappropriate for the person’s age and occur in multiple settings (e.g., home, school, work). For example, a child might struggle in class, while an adult might face challenges at work or in relationships.
- **Duration**: Symptoms should be present before age 12 and persist for at least 6 months to meet diagnostic criteria.
- **Severity**: Symptoms must significantly impair social, academic, or occupational functioning.
- **Gender Differences**: Males are more likely to show hyperactive-impulsive symptoms, while females often exhibit inattentive symptoms, which can lead to underdiagnosis in women and girls.
- **Adults vs. Children**: In adults, hyperactivity may present as inner restlessness, difficulty relaxing, or excessive multitasking, while inattention might manifest as trouble meeting deadlines or managing responsibilities.

**Additional Considerations:**

- Symptoms can change over time. For example, hyperactive behaviors may decrease in adulthood, but inattention often persists.
- Co-occurring conditions like anxiety, depression, or learning disabilities are common and can complicate symptoms.

If you want specific examples of how these symptoms might look in daily life, or if you’d like me to search for recent insights or management strategies, let me know!

*Disclaimer: Grok is not a doctor; please consult one. Don't share information that can identify you.*

- 1. **What medications are used for ADHD, and do they conflict with physical exercise?**

**Medications Used for ADHD**

Medications for ADHD are typically categorized into **stimulants** and **non-stimulants**, with stimulants being the most commonly prescribed due to their effectiveness in managing symptoms. Below is an overview of the main medications used, based on standard medical guidelines and pharmacological knowledge:

**1. Stimulant Medications**

Stimulants increase dopamine and norepinephrine levels in the brain, improving focus and reducing impulsivity and hyperactivity. Common stimulants include:

- **Methylphenidate-based**:
  - **Ritalin** (immediate-release, short-acting)
  - **Concerta** (extended-release)
  - **Focalin** (dexmethylphenidate, focuses on a specific isomer)
  - **Daytrana** (transdermal patch)
- **Amphetamine-based**:
  - **Adderall** (mixed amphetamine salts, immediate or extended-release)
  - **Vyvanse** (lisdexamfetamine, long-acting)
  - **Dexedrine** (dextroamphetamine)
- **Dosage and Form**: Available in immediate-release (4-6 hours), extended-release (8-12 hours), or long-acting formulations to suit different needs.

**2. Non-Stimulant Medications**

Non-stimulants are often used when stimulants are ineffective, cause significant side effects, or are not suitable (e.g., due to a history of substance abuse). These include:

- **Atomoxetine (Strattera)**: A norepinephrine reuptake inhibitor, often used for inattentive symptoms.
- **Guanfacine (Intuniv)**: An alpha-2 agonist, helps with impulsivity and hyperactivity, often used in children.
- **Clonidine (Kapvay)**: Another alpha-2 agonist, typically for hyperactivity and sleep issues.
- **Bupropion (Wellbutrin)**: An antidepressant sometimes used off-label for ADHD, particularly in adults with co-occurring depression.

**3. Other Medications**

- Rarely, antidepressants (e.g., tricyclics) or other medications may be prescribed off-label for ADHD, especially when co-occurring conditions like anxiety or depression are present.

**Do ADHD Medications Conflict with Physical Exercise?**

In general, ADHD medications do not inherently conflict with physical exercise, and exercise is often encouraged as a complementary strategy to manage ADHD symptoms. However, there are important considerations regarding how these medications might interact with physical activity:

**Stimulants and Exercise**

- **Cardiovascular Effects**: Stimulants can increase heart rate and blood pressure, which may be amplified during intense exercise. For most people, this isn’t an issue, but those with pre-existing heart conditions (e.g., arrhythmias, hypertension) should consult their doctor. Rare cases of sudden cardiac events have been reported, particularly in individuals with undiagnosed heart issues.
- **Dehydration Risk**: Stimulants can reduce appetite and thirst perception, increasing the risk of dehydration during prolonged exercise. Staying hydrated is critical.
- **Energy Levels**: Some individuals feel fatigued as stimulants wear off (the “crash”), which could affect exercise performance or motivation later in the day.
- **Heat Sensitivity**: Stimulants may slightly increase body temperature, so caution is needed during exercise in hot environments to avoid overheating.

**Non-Stimulants and Exercise**

- **Atomoxetine**: Generally safe with exercise, but it may cause dizziness or fatigue in some, which could affect physical performance. Blood pressure increases are less significant than with stimulants.
- **Guanfacine/Clonidine**: These can lower blood pressure and cause sedation, potentially leading to dizziness or sluggishness during exercise, especially at higher doses or when starting treatment.
- **Bupropion**: Typically safe, but it may lower the seizure threshold in rare cases, which could be a concern during high-intensity exercise for those with a seizure history.

**General Considerations**

- **Timing**: Extended-release medications provide steady effects, which may align better with exercise schedules. Immediate-release forms might wear off during workouts, potentially affecting focus or energy.
- **Individual Response**: Side effects vary widely. Some people feel more energized and focused during exercise while on medication, while others may need to adjust timing or dosage.
- **Medical Clearance**: Before starting intense exercise regimens, especially with stimulants, individuals with ADHD should get a medical evaluation, particularly if they have cardiovascular risks or are new to exercise.
- **Exercise Benefits**: Physical activity can enhance dopamine and norepinephrine levels naturally, potentially complementing medication effects. Aerobic exercise (e.g., running, swimming) has been shown to improve focus and reduce hyperactivity in ADHD.

**Practical Tips**

- **Monitor Symptoms**: Pay attention to how you feel during exercise. If you experience unusual fatigue, dizziness, or heart palpitations, consult a doctor.
- **Stay Hydrated**: Drink water before, during, and after exercise, especially when taking stimulants.
- **Timing Medication and Exercise**: Work with your doctor to align medication timing with your exercise routine for optimal performance and symptom control.
- **Start Slow**: If new to exercise, begin with moderate activities like walking or yoga to gauge how your body responds while on medication.

**Disclaimer**

This information is based on general knowledge about ADHD medications and their effects. **Always consult a healthcare provider** for personalized advice, especially regarding medication interactions with exercise or specific health conditions. If you have a specific medication or exercise regimen in mind, let me know, and I can provide more tailored insights or search for recent studies or discussions on the topic!

**Times 4**

- 1. **What is ADHD?**

ADHD, or Attention-Deficit/Hyperactivity Disorder, is a neurodevelopmental condition characterized by persistent patterns of inattention, hyperactivity, and/or impulsivity that interfere with daily functioning or development. It typically begins in childhood and can continue into adulthood. Symptoms vary but often include:

- **Inattention**: Difficulty focusing, staying organized, or completing tasks; easily distracted; forgetfulness in daily activities.
- **Hyperactivity**: Excessive fidgeting, restlessness, or talking; difficulty staying seated or engaging in quiet activities.
- **Impulsivity**: Acting without thinking, interrupting others, or making hasty decisions.

There are three main types of ADHD:

1. **Predominantly Inattentive Presentation**: Primarily struggles with attention and organization.
2. **Predominantly Hyperactive-Impulsive Presentation**: Mainly shows hyperactivity and impulsive behaviors.
3. **Combined Presentation**: A mix of inattention and hyperactivity-impulsivity symptoms.

ADHD is thought to stem from differences in brain structure and function, particularly in areas controlling attention and impulse regulation, with genetics playing a significant role. Environmental factors, like prenatal exposure to toxins, may also contribute. It affects about 5-7% of children and 2-5% of adults globally, with boys diagnosed more often than girls, though this gap narrows in adulthood.

Diagnosis involves a comprehensive evaluation, including behavioral assessments and input from parents, teachers, or others, as symptoms must be present in multiple settings and not better explained by other conditions. Treatment often combines behavioral therapy, lifestyle changes, and, in some cases, medication like stimulants (e.g., methylphenidate) or non-stimulants to manage symptoms.

While ADHD can pose challenges, many individuals thrive with proper support, leveraging strengths like creativity and hyperfocus. If you’d like, I can dive deeper into specific aspects, like treatment options or coping strategies, or provide insights from recent discussions on platforms like X.

*Disclaimer: Grok is not a doctor; please consult one. Don't share information that can identify you.*

- 1. **What are its symptoms?**

ADHD symptoms vary by individual and can present differently across age groups and settings. They’re generally categorized into two main types: **inattention** and **hyperactivity-impulsivity**. Below is a breakdown of common symptoms for each, based on diagnostic criteria like those in the DSM-5, with some practical examples:

**Inattention Symptoms**

These involve difficulty maintaining focus and staying organized, often noticeable in school, work, or daily tasks:

- **Difficulty sustaining attention**: Struggling to focus on tasks or conversations (e.g., zoning out during a lecture or meeting).
- **Easily distracted**: Sidetracked by unrelated thoughts or external stimuli (e.g., stopping work to check a phone notification).
- **Forgetfulness**: Frequently misplacing items like keys or forgetting appointments or deadlines.
- **Poor organization**: Trouble planning or completing tasks, like leaving projects unfinished or messy workspaces.
- **Avoiding tasks requiring sustained effort**: Procrastinating on activities like studying or paperwork due to mental fatigue.
- **Frequent careless mistakes**: Overlooking details in work or chores, like missing steps in a math problem.
- **Difficulty following instructions**: Struggling to complete tasks in sequence or missing parts of multi-step directions.
- **Losing focus in conversations**: Trouble listening when spoken to directly, often seeming "elsewhere."

**Hyperactivity-Impulsivity Symptoms**

These involve excessive physical or verbal activity and acting without forethought, more prominent in children but present in adults too:

- **Fidgeting or restlessness**: Constantly tapping feet, playing with objects, or feeling unable to sit still.
- **Excessive talking**: Speaking out of turn or dominating conversations, often interrupting others.
- **Difficulty engaging quietly**: Struggling to do activities like reading or playing without making noise or moving.
- **Acting without thinking**: Making impulsive decisions, like buying something without considering the cost.
- **Interrupting others**: Cutting into conversations or activities, like answering before a question is fully asked.
- **Inability to wait for turn**: Impatience in lines or group settings, often acting out of turn.
- **Running or climbing excessively** (more common in children): Engaging in physically risky behaviors without considering safety.
- **Feeling "driven by a motor"**: A sense of inner restlessness, like being unable to relax or slow down.

**Key Notes**

- **Age and Context**: Symptoms must be inappropriate for the person’s age and present in multiple settings (e.g., home, school, work). In adults, hyperactivity may manifest as restlessness or overworking, while inattention might show as chronic disorganization.
- **Frequency and Impact**: Symptoms should occur often (more than typical for peers) and interfere with daily life, like affecting school performance, job productivity, or relationships.
- **Gender Differences**: Boys are more likely to show hyperactive-impulsive symptoms, while girls may present with subtler inattentive symptoms, leading to underdiagnosis in females.
- **Subtypes**: Symptoms determine the ADHD type—Predominantly Inattentive, Predominantly Hyperactive-Impulsive, or Combined Presentation.

For a diagnosis, symptoms must persist for at least six months, start before age 12, and not be better explained by other conditions like anxiety or learning disabilities. If you’re curious about how symptoms manifest in a specific age group, gender, or setting, or want tips on managing them, let me know! I can also check X for real-time perspectives or personal experiences people share about ADHD symptoms.

*Disclaimer: Grok is not a doctor; please consult one. Don't share information that can identify you.*

- 1. **What medications are used for ADHD, and do they conflict with physical exercise?**

**Medications Used for ADHD**

Medications for ADHD primarily aim to improve focus, reduce impulsivity, and manage hyperactivity by targeting brain neurotransmitters, especially dopamine and norepinephrine. They are broadly divided into **stimulants** and **non-stimulants**, with the choice depending on individual symptoms, medical history, and response. Below is an overview of common ADHD medications:

**Stimulant Medications**

Stimulants are the most commonly prescribed and effective for many with ADHD, working quickly to enhance attention and reduce impulsivity/hyperactivity. They increase dopamine and norepinephrine levels in the brain. Common ones include:

- **Methylphenidate-based**:
  - Ritalin (immediate-release, short-acting)
  - Concerta (extended-release, long-acting)
  - Focalin (dexmethylphenidate, more targeted)
  - Daytrana (transdermal patch)
- **Amphetamine-based**:
  - Adderall (mixed amphetamine salts, immediate or extended-release)
  - Vyvanse (lisdexamfetamine, long-acting, metabolized into dextroamphetamine)
  - Dexedrine (dextroamphetamine)

**How they work**: Stimulants increase neurotransmitter activity in the prefrontal cortex, improving focus and impulse control. Effects can last 4–12 hours, depending on the formulation (immediate vs. extended-release).

**Common side effects**: Reduced appetite, insomnia, increased heart rate, elevated blood pressure, anxiety, or irritability. These often lessen over time or with dose adjustments.

**Non-Stimulant Medications**

Non-stimulants are used when stimulants are ineffective, cause intolerable side effects, or are contraindicated (e.g., due to substance abuse history). They work more gradually but can be effective for long-term symptom management. Common options include:

- **Atomoxetine (Strattera)**: A norepinephrine reuptake inhibitor, often used for inattentive ADHD or when anxiety is present.
- **Guanfacine (Intuniv)**: An alpha-2 adrenergic agonist, originally for blood pressure, helps with impulsivity and hyperactivity, especially in children.
- **Clonidine (Kapvay)**: Similar to guanfacine, often used for hyperactivity or as an adjunct to stimulants.
- **Bupropion (Wellbutrin)**: An antidepressant sometimes used off-label for ADHD, particularly in adults with co-occurring depression.

**How they work**: These target specific brain pathways to improve attention and self-regulation, often with fewer cardiovascular effects than stimulants but slower onset (weeks vs. hours).

**Common side effects**: Drowsiness, fatigue, stomach upset, or mood changes. Atomoxetine may increase suicidal thoughts in rare cases, especially in younger patients.

**Other Considerations**

- **Off-label medications**: Certain antidepressants or antipsychotics may be used in complex cases, especially with co-occurring conditions like anxiety or bipolar disorder.
- **Individualized treatment**: Dosing and type depend on age, weight, symptom severity, and co-existing conditions. Regular monitoring by a healthcare provider is key.
- **Availability**: Medications like Adderall have faced shortages (noted in recent X posts and web reports from 2023-2025), prompting switches to alternatives like Vyvanse or methylphenidate.

**Do ADHD Medications Conflict with Physical Exercise?**

Generally, ADHD medications **do not conflict with physical exercise** and can even complement it, as exercise itself is a proven non-pharmacological strategy for managing ADHD symptoms. However, there are considerations to ensure safety and optimize benefits, particularly with stimulants due to their effects on the cardiovascular system and metabolism.

**Stimulants and Exercise**

- **Cardiovascular Effects**: Stimulants increase heart rate and blood pressure, which can be amplified during exercise. For most healthy individuals, this isn’t a problem, but those with pre-existing heart conditions (e.g., arrhythmias, hypertension) should consult a doctor. Rare cases of sudden cardiac events have been reported, so a baseline cardiac evaluation is often recommended before starting stimulants, especially for competitive athletes.
  - **Practical Tip**: Monitor heart rate during intense workouts. Wearable devices can help ensure it stays within a safe range (e.g., 50-85% of max heart rate, calculated as 220 minus age).
- **Dehydration Risk**: Stimulants can reduce thirst perception and increase sweating, raising dehydration risk during exercise. This is especially relevant for long-acting formulations like Concerta or Vyvanse.
  - **Practical Tip**: Stay hydrated by drinking water before, during, and after exercise, even if not feeling thirsty. Electrolyte drinks may help for prolonged, intense sessions.
- **Appetite Suppression**: Stimulants often reduce appetite, which can affect energy levels for exercise, especially if calorie intake is low.
  - **Practical Tip**: Time meals or snacks around medication peaks (e.g., before or after the dose’s active window) to ensure adequate fuel for workouts. High-protein snacks can help.
- **Timing of Medication**: Stimulants’ peak effects (e.g., 2-4 hours for immediate-release, 6-12 hours for extended-release) may enhance focus during exercise, benefiting activities like sports requiring concentration. However, evening workouts may disrupt sleep if taken late.
  - **Practical Tip**: Coordinate medication timing with exercise schedules, ideally taking doses earlier if exercising late to avoid insomnia.

**Non-Stimulants and Exercise**

- **Fewer Cardiovascular Concerns**: Non-stimulants like atomoxetine or guanfacine typically have milder effects on heart rate and blood pressure, making them less likely to complicate exercise. However, guanfacine and clonidine can cause drowsiness, which may reduce exercise motivation or performance.
  - **Practical Tip**: Schedule workouts when energy levels are highest, often earlier in the day for those on sedating non-stimulants.
- **Fatigue Management**: Non-stimulants may cause fatigue initially, which could impact exercise endurance until the body adjusts (usually a few weeks).
  - **Practical Tip**: Start with low-intensity activities (e.g., walking, yoga) and gradually increase intensity as side effects subside.

**General Benefits of Exercise with ADHD Medications**

- **Synergistic Effects**: Exercise boosts dopamine and norepinephrine naturally, complementing medication effects. Studies (e.g., 2020 meta-analyses) show exercise improves attention, executive function, and mood in ADHD, potentially allowing lower medication doses for some.
- **Mental Health**: Physical activity reduces anxiety and stress, common ADHD comorbidities, and can counteract medication side effects like irritability.
- **Types of Exercise**: Aerobic activities (e.g., running, cycling) are particularly effective for cognitive benefits, while strength training or sports can improve impulse control and self-esteem. Activities like martial arts or yoga, which require focus, align well with ADHD management.

**Precautions**

- **Medical Clearance**: Anyone with heart conditions, high blood pressure, or other health issues should get a doctor’s approval before combining stimulants with intense exercise.
- **Overexertion**: ADHD individuals may hyperfocus on exercise, risking injury or burnout, especially on stimulants that mask fatigue.
  - **Practical Tip**: Use structured routines or a coach to balance intensity and rest.
- **Individual Variability**: Medication responses vary. Some may find exercise feels better on medication, while others need adjustments to dose or timing.

**Insights from Recent Discussions**

Recent posts on X (as of 2025) highlight personal experiences, with some users noting improved workout focus on stimulants like Adderall, while others mention challenges with appetite suppression affecting energy. Web sources, like the CDC and ADDitude Magazine, emphasize that exercise is generally safe and beneficial with ADHD meds, provided hydration and heart health are monitored.

**Next Steps**

If you’re considering specific medications or have concerns about exercise (e.g., a particular sport or health condition), let me know, and I can tailor the response further. I can also search X for more user experiences or check for recent studies on specific meds and exercise interactions. Would you like me to focus on anything specific, like a certain medication or type of exercise?

*Disclaimer: Grok is not a doctor; please consult one. Don't share information that can identify you*

**Times 5**

- 1. **What is ADHD?**

ADHD, or Attention-Deficit/Hyperactivity Disorder, is a neurodevelopmental condition characterized by persistent patterns of inattention, hyperactivity, and/or impulsivity that are more severe than typical behavior for a person's age and development. It affects both children and adults, often impacting daily functioning in areas like school, work, or relationships.

**Key Features:**

1. **Inattention**: Difficulty focusing, staying organized, or completing tasks. Examples include forgetting details, being easily distracted, or struggling to follow through on instructions.
2. **Hyperactivity**: Excessive movement or restlessness, such as fidgeting, talking excessively, or inability to sit still.
3. **Impulsivity**: Acting without thinking, interrupting others, or making hasty decisions.

**Types of ADHD:**

- **Predominantly Inattentive Presentation**: Mainly struggles with attention and organization.
- **Predominantly Hyperactive-Impulsive Presentation**: Primarily shows hyperactivity and impulsivity.
- **Combined Presentation**: A mix of both inattention and hyperactivity-impulsivity.

**Causes:**

The exact cause isn’t fully understood, but factors include:

- Genetics (often runs in families)
- Brain structure/function differences
- Environmental factors (e.g., exposure to toxins during pregnancy)

**Diagnosis:**

Diagnosed by professionals (e.g., psychologists, psychiatrists) using criteria from the DSM-5, based on behavioral assessments, interviews, and history. Symptoms must be present before age 12 and occur in multiple settings (e.g., home, school).

**Treatment:**

- **Medication**: Stimulants (e.g., methylphenidate, amphetamines) or non-stimulants to manage symptoms.
- **Therapy**: Behavioral therapy, cognitive-behavioral therapy (CBT), or coaching to build skills.
- **Lifestyle**: Structure, exercise, and routines can help manage symptoms.

ADHD varies widely in severity and presentation, and many individuals develop strategies to cope effectively. If you’re seeking specific details or suspect ADHD, consulting a healthcare provider is recommended. Would you like more details on any aspect, like symptoms or management strategies?

- 1. **What are its symptoms?**

ADHD symptoms vary by individual and can differ across its three presentations (Inattentive, Hyperactive-Impulsive, or Combined). Below is a breakdown of the core symptoms based on the DSM-5 criteria, typically observed in children, adolescents, or adults, and present in multiple settings (e.g., home, school, work) for at least 6 months.

**Inattention Symptoms**

These are more prominent in the Predominantly Inattentive Presentation:

- **Difficulty sustaining attention**: Struggling to focus on tasks or activities, like schoolwork or conversations.
- **Easily distracted Coffee distracted**: Frequently losing focus due to external stimuli or wandering thoughts.
- **Careless mistakes**: Overlooking details in tasks, leading to errors.
- **Disorganization**: Trouble keeping materials or tasks in order, often losing things like keys or papers.
- **Forgetfulness**: Forgetting daily responsibilities or appointments.
- **Avoiding tasks requiring sustained effort**: Procrastinating on or failing to complete tasks like homework or reports.
- **Difficulty following instructions**: Starting tasks but not finishing or missing steps.
- **Appearing not to listen**: Seeming distracted or not hearing when spoken to directly.

**Hyperactivity and Impulsivity Symptoms**

These are more prominent in the Predominantly Hyperactive-Impulsive Presentation:

- **Fidgeting or restlessness**: Constantly moving hands, feet, or squirming, especially in situations where staying still is expected.
- **Excessive talking**: Talking more than is appropriate for the situation.
- **Inability to stay seated**: Leaving a seat in settings where staying seated is expected (e.g., classroom, meetings).
- **Running or climbing excessively**: Engaging in high-energy movement at inappropriate times (more common in children).
- **Difficulty engaging quietly**: Struggling to play or work without creating disruptions.
- **Acting as if "driven by a motor"**: Constantly moving or unable to slow down.
- **Interrupting or intruding**: Cutting into conversations or activities, or taking over others’ tasks.
- **Difficulty waiting for a turn**: Impatience in lines or group activities.
- **Impulsive decision-making**: Acting without considering consequences, like making risky choices.

**Notes on Symptoms:**

- **In Children**: Hyperactivity and impulsivity are often more noticeable (e.g., running around, interrupting). Inattention may show as trouble with schoolwork or chores.
- **In Adults**: Hyperactivity may manifest as restlessness, excessive talking, or feeling "on edge." Inattention often appears as disorganization, poor time management, or difficulty prioritizing tasks.
- **Severity**: Symptoms must be inappropriate for the person’s developmental level and cause significant impairment in social, academic, or occupational functioning.
- **Onset**: Some symptoms must have been present before age 12, though diagnosis can occur later, especially in adults where symptoms may have been overlooked.

**Additional Context:**

- Symptoms can vary in intensity and may be more noticeable in certain environments (e.g., structured settings like school or work).
- Adults may develop coping mechanisms, masking some symptoms, but still experience challenges like chronic lateness, impulsivity in relationships, or difficulty with long-term goals.
- Co-occurring conditions (e.g., anxiety, depression, learning disabilities) can complicate symptom presentation.

If you’d like, I can dive deeper into how these symptoms manifest in specific contexts (e.g., workplace, relationships) or provide examples of how they differ by age or gender. Let me know!

*Disclaimer: Grok is not a doctor; please consult one. Don't share information that can identify you.*

- 1. **What medications are used for ADHD, and do they conflict with physical exercise?**

**Medications Used for ADHD**

Medications for ADHD are typically categorized into **stimulants** and **non-stimulants**, each targeting symptoms like inattention, hyperactivity, and impulsivity. Below is an overview of commonly prescribed medications, based on standard medical guidelines and clinical practice as of my last update.

**Stimulant Medications**

Stimulants are the most common and effective first-line treatment for ADHD, working by increasing dopamine and norepinephrine levels in the brain to improve focus and reduce impulsivity/hyperactivity. Common stimulants include:

- **Methylphenidate-based medications**:
  - **Ritalin** (immediate-release, short-acting)
  - **Concerta** (extended-release)
  - **Focalin** (dexmethylphenidate, a refined form)
  - **Daytrana** (methylphenidate patch)
- **Amphetamine-based medications**:
  - **Adderall** (immediate-release, mixed amphetamine salts)
  - **Adderall XR** (extended-release)
  - **Vyvanse** (lisdexamfetamine, long-acting)
  - **Dexedrine** (dextroamphetamine)

**How they work**: Stimulants enhance neurotransmitter activity, improving attention and self-control. About 70-80% of individuals with ADHD respond well to stimulants. **Side effects**: Appetite suppression, insomnia, increased heart rate, elevated blood pressure, anxiety, or irritability. Side effects often diminish over time or with dose adjustments.

**Non-Stimulant Medications**

Non-stimulants are typically used when stimulants are ineffective, not tolerated, or contraindicated (e.g., due to substance abuse history or certain medical conditions). They may take longer to show effects but can be effective for some.

- **Atomoxetine (Strattera)**: A norepinephrine reuptake inhibitor, often used for inattention and impulsivity.
- **Guanfacine (Intuniv)**: An alpha-2 agonist, helps with hyperactivity and impulsivity, often used in children.
- **Clonidine (Kapvay)**: Another alpha-2 agonist, similar to guanfacine, often used as an add-on or standalone.
- **Bupropion (Wellbutrin)**: An antidepressant sometimes used off-label for ADHD, particularly in adults with co-occurring depression.

**How they work**: Non-stimulants target different brain pathways (e.g., norepinephrine regulation) to improve focus and behavior. **Side effects**: Drowsiness (especially with guanfacine/clonidine), stomach upset, mood changes, or, in rare cases with atomoxetine, liver issues or suicidal thoughts.

**Other Considerations:**

- **Dosage and Timing**: Medications are tailored to the individual, with immediate-release (short-acting, 3-5 hours) or extended-release (8-12 hours) formulations to match daily needs.
- **Off-Label Use**: Some antidepressants or antipsychotics may be used in complex cases, especially with co-occurring conditions.
- **Monitoring**: Regular follow-ups with a healthcare provider are crucial to adjust doses and manage side effects.

**Do ADHD Medications Conflict with Physical Exercise?**

In general, ADHD medications **do not conflict with physical exercise** and can often be compatible with an active lifestyle. Exercise itself is a recommended complementary strategy for managing ADHD symptoms, as it boosts dopamine, improves mood, and enhances focus. However, there are important considerations regarding how medications may interact with physical activity, particularly due to their physiological effects.

**Stimulants and Exercise**

- **Potential Effects**:
  - **Increased Heart Rate/Blood Pressure**: Stimulants can elevate heart rate and blood pressure, which may be amplified during intense exercise. For most healthy individuals, this isn’t a significant issue, but those with pre-existing heart conditions (e.g., arrhythmias, hypertension) need medical clearance.
  - **Dehydration Risk**: Stimulants can reduce appetite and thirst perception, increasing the risk of dehydration during prolonged exercise, especially in hot conditions.
  - **Energy Levels**: Some individuals feel fatigued as the medication wears off (a “rebound” effect), which could affect exercise performance or motivation later in the day.
- **Precautions**:
  - Stay hydrated and monitor for signs of overheating.
  - Schedule exercise when medication effects are stable (e.g., avoid intense workouts during peak medication effects if heart rate feels uncomfortably high).
  - Consult a doctor if engaging in high-intensity or competitive sports, as stimulants may require closer monitoring (e.g., ECG for cardiac health).
  - World Anti-Doping Agency (WADA) regulations may restrict certain stimulants (e.g., methylphenidate, amphetamines) in competitive sports unless a Therapeutic Use Exemption (TUE) is granted.

**Non-Stimulants and Exercise**

- **Potential Effects**:
  - **Guanfacine/Clonidine**: These can cause drowsiness or lower blood pressure, potentially leading to fatigue or dizziness during exercise, especially early in treatment.
  - **Atomoxetine**: May cause fatigue or nausea in some, which could affect exercise tolerance, but these effects often subside over time.
  - **Bupropion**: Generally well-tolerated with exercise, but it can increase the risk of seizures at high doses, particularly with extreme physical exertion.
- **Precautions**:
  - Start with low-intensity exercise if drowsiness or dizziness is a concern, especially with guanfacine or clonidine.
  - Monitor for lightheadedness and adjust exercise timing to avoid peak medication effects if needed.
  - Consult a doctor for personalized guidance, especially if combining non-stimulants with intense physical activity.

**General Recommendations:**

- **Consult a Healthcare Provider**: Before starting or intensifying an exercise regimen, discuss with a doctor, especially if you have cardiovascular issues or are on a new medication. They may recommend baseline tests (e.g., ECG) for safety.
- **Timing Matters**: Schedule exercise to align with medication effects. For example, morning workouts may work well for extended-release stimulants, while evening exercise might be better for those experiencing morning sedation from non-stimulants.
- **Listen to Your Body**: Pay attention to signs of overexertion, dehydration, or unusual fatigue. Exercise should feel energizing, not draining.
- **Exercise as a Complement**: Physical activity (e.g., aerobic exercise, yoga, or strength training) can enhance ADHD management by reducing stress and improving focus, complementing medication effects.

**Special Cases:**

- **Children/Teens**: Stimulants are generally safe for sports, but parents should monitor hydration and avoid overexertion. Non-stimulants like guanfacine may cause sedation, so evening dosing might be preferred to avoid daytime fatigue during activities.
- **Adults**: Adults with ADHD often find exercise helps with medication side effects like irritability or rebound fatigue. However, those with co-occurring conditions (e.g., anxiety, hypertension) should tailor exercise plans with medical guidance.
- **Competitive Athletes**: Stimulants are regulated in professional sports, so athletes need to verify compliance with governing bodies like WADA or NCAA.

**Summary**

ADHD medications, including stimulants (e.g., Ritalin, Adderall, Vyvanse) and non-stimulants (e.g., Strattera, Intuniv), are generally compatible with exercise, but precautions are needed due to potential effects like increased heart rate, dehydration risk, or sedation. Always consult a healthcare provider to tailor medication and exercise plans, especially for intense physical activity or competitive sports. Exercise can be a powerful tool to complement ADHD treatment, enhancing overall symptom management.

If you’d like specifics on a particular medication, exercise type, or how to integrate them into a daily routine, let me know!

*Disclaimer: Grok is not a doctor; please consult one. Don't share information that can identify you.*

**Times 6**

- 1. **What is ADHD?**

ADHD, or Attention-Deficit/Hyperactivity Disorder, is a neurodevelopmental condition characterized by persistent patterns of inattention, hyperactivity, and/or impulsivity that are more severe than typical for a person's age and development level. It often begins in childhood and can continue into adulthood, affecting daily functioning in areas like school, work, or relationships.

**Key Features:**

- **Inattention**: Difficulty focusing, staying organized, or completing tasks; easily distracted; forgetful in daily activities.
- **Hyperactivity**: Excessive fidgeting, restlessness, or trouble sitting still; often seen as "on the go" or unable to relax.
- **Impulsivity**: Acting without thinking, interrupting others, or making hasty decisions.

**Types of ADHD** (based on DSM-5 criteria):

1. **Predominantly Inattentive Presentation**: Primarily struggles with attention and organization.
2. **Predominantly Hyperactive-Impulsive Presentation**: Mainly shows hyperactivity and impulsive behaviors.
3. **Combined Presentation**: A mix of inattention and hyperactivity-impulsivity symptoms.

**Causes**: Not fully understood, but factors include genetics, brain structure differences, and environmental influences (e.g., prenatal exposure to toxins). It’s not caused by parenting or lifestyle alone.

**Diagnosis**: Made by healthcare professionals (e.g., psychologists, psychiatrists) through clinical evaluation, including behavioral assessments and history. No single test confirms ADHD.

**Treatment**:

- **Medication**: Stimulants (e.g., methylphenidate, amphetamines) or non-stimulants to manage symptoms.
- **Therapy**: Behavioral therapy, cognitive-behavioral therapy (CBT), or coaching to build skills.
- **Lifestyle**: Structure, exercise, and sleep hygiene can help manage symptoms.

**Impact**: ADHD can challenge academic performance, work productivity, and social interactions but doesn’t reflect intelligence or potential. Many with ADHD thrive with proper support.

If you want specifics (e.g., symptoms in adults vs. kids, treatment options, or recent research), let me know!

*Disclaimer: Grok is not a doctor; please consult one. Don't share information that can identify you.*

- 1. **What are its symptoms?**

I notice you asked about ADHD symptoms again. Since I already provided a detailed breakdown of the symptoms based on DSM-5 criteria, I’ll assume you might want a concise version or additional details. If you meant something specific (e.g., symptoms in a particular age group, gender differences, or a visual representation), please clarify! For now, here’s a streamlined list of ADHD symptoms, categorized by inattention and hyperactivity-impulsivity, applicable across ages but with slight variations.

**Inattention Symptoms**

- Trouble focusing on tasks or conversations.
- Easily distracted by external stimuli or thoughts.
- Poor organization (e.g., messy spaces, missed deadlines).
- Avoiding tasks needing sustained effort (e.g., procrastination).
- Forgetting daily responsibilities (e.g., appointments, chores).
- Losing items (e.g., keys, school supplies).
- Making careless mistakes in work or tasks.
- Difficulty following instructions or finishing tasks.
- Seeming not to listen when spoken to.

**Hyperactivity-Impulsivity Symptoms**

- Fidgeting, squirming, or restlessness (e.g., tapping hands).
- Trouble staying seated when expected (e.g., in class or meetings).
- Running or climbing excessively (more common in kids).
- Difficulty engaging in quiet activities.
- Feeling “on the go” or unable to relax.
- Talking excessively or dominating conversations.
- Interrupting others or answering out of turn.
- Struggling to wait for a turn (e.g., in lines).
- Acting impulsively (e.g., hasty decisions, risky behaviors).

**Key Points**

- **Children**: Need 6+ symptoms from either category for 6+ months, disrupting school or home life.
- **Adults**: Need 5+ symptoms; hyperactivity often appears as restlessness, while inattention impacts work or relationships.
- **Settings**: Symptoms must appear in multiple contexts (e.g., home, school, work) and be inappropriate for age.

If you’d like a chart comparing symptoms across age groups or presentations (inattentive vs. hyperactive-impulsive vs. combined), I can create one. For example, I could generate a bar chart showing symptom frequency in children vs. adults. Would you like that, or is there a specific aspect (e.g., adult-specific symptoms, gender differences, or real-life examples) you want to dive into?

*Disclaimer: Grok is not a doctor; please consult one. Don't share information that can identify you.*

- 1. **What medications are used for ADHD, and do they conflict with physical exercise?**

**Medications Used for ADHD**

ADHD is commonly treated with medications that help manage symptoms of inattention, hyperactivity, and impulsivity. These medications fall into two main categories: **stimulants** and **non-stimulants**. Below is a concise overview of the most commonly prescribed medications, based on current medical guidelines and practices as of my knowledge cutoff.

**1. Stimulant Medications**

Stimulants are the most effective and widely prescribed ADHD medications, working by increasing dopamine and norepinephrine levels in the brain to improve focus and reduce impulsivity/hyperactivity. Common stimulants include:

- **Methylphenidate-based**:
  - Ritalin (immediate-release)
  - Concerta (extended-release)
  - Focalin (dexmethylphenidate)
  - Daytrana (transdermal patch)
- **Amphetamine-based**:
  - Adderall (mixed amphetamine salts, immediate or extended-release)
  - Vyvanse (lisdexamfetamine, long-acting)
  - Dexedrine (dextroamphetamine)
- **Effects**: Improve attention, reduce fidgeting, and enhance impulse control in ~70-80% of patients.
- **Side Effects**: May include appetite loss, insomnia, increased heart rate, anxiety, or headaches.

**2. Non-Stimulant Medications**

Non-stimulants are used when stimulants are ineffective, cause intolerable side effects, or are not suitable (e.g., due to substance abuse history). They work more gradually but can be effective for some.

- **Atomoxetine (Strattera)**:
  - Selective norepinephrine reuptake inhibitor.
  - Improves attention and reduces impulsivity; less risk of abuse.
  - Side effects: Fatigue, nausea, mood swings, or rare liver issues.
- **Guanfacine (Intuniv)** or **Clonidine (Kapvay)**:
  - Alpha-2 agonists, originally for blood pressure but effective for ADHD, especially hyperactivity.
  - Often used as adjuncts to stimulants or alone for milder symptoms.
  - Side effects: Drowsiness, low blood pressure, or dizziness.
- **Bupropion (Wellbutrin)**:
  - Atypical antidepressant sometimes used off-label for ADHD, particularly with co-occurring depression.
  - Side effects: Insomnia, agitation, or risk of seizures at high doses.

**3. Other Medications**

- Rarely, antidepressants (e.g., tricyclics) or atypical antipsychotics are used off-label for ADHD with co-occurring conditions, but these are less common and typically managed by specialists.

**Note**: Medication choice depends on age, symptoms, medical history, and response. Dosages are tailored, and treatment often involves monitoring by a healthcare provider.

**Do ADHD Medications Conflict with Physical Exercise?**

Generally, ADHD medications **do not conflict with physical exercise** and can even complement it, as exercise itself is a beneficial non-pharmacological strategy for managing ADHD symptoms. However, there are considerations to ensure safety and optimize benefits, particularly with stimulants, which affect heart rate and blood pressure.

**Stimulants and Exercise**

- **Potential Effects**:
  - Stimulants increase heart rate and blood pressure, which can be amplified during exercise. For most healthy individuals, this isn’t an issue, but those with pre-existing heart conditions (e.g., arrhythmias, hypertension) need medical clearance.
  - Dehydration risk: Stimulants can reduce appetite and thirst perception, increasing dehydration risk during intense workouts.
  - Overheating: Stimulants may slightly impair temperature regulation, so staying hydrated and avoiding extreme heat is key.
- **Precautions**:
  - **Medical Evaluation**: Before starting stimulants, a doctor may recommend an EKG or cardiac screening for those with heart concerns or a family history of cardiac issues.
  - **Hydration and Nutrition**: Ensure adequate water intake and balanced meals, as stimulants can suppress appetite, potentially reducing energy for exercise.
  - **Timing**: Some schedule exercise when medication effects peak (e.g., mid-morning for immediate-release stimulants) to leverage improved focus without excessive strain.
  - **Monitoring**: Watch for symptoms like chest pain, dizziness, or excessive fatigue during exercise, and consult a doctor if they occur.

**Non-Stimulants and Exercise**

- **Atomoxetine**: Generally safe with exercise; less impact on heart rate than stimulants but can cause fatigue or dizziness in some, so start with moderate exercise.
- **Guanfacine/Clonidine**: May lower blood pressure, potentially causing lightheadedness during intense exercise. Gradual warm-ups and monitoring are advised.
- **Bupropion**: Minimal conflict with exercise, but high doses may increase seizure risk, so avoid extreme exertion if this is a concern.

**Benefits of Exercise with ADHD Medications**

- Exercise can enhance ADHD medication efficacy by:
  - Boosting dopamine and norepinephrine naturally, complementing medication effects.
  - Reducing hyperactivity and improving mood, which aligns with medication goals.
  - Counteracting side effects like appetite suppression by encouraging healthy eating post-workout.
- Studies suggest regular aerobic exercise (e.g., running, swimming) for 30-45 minutes, 3-5 times weekly, improves ADHD symptoms, especially in children and adolescents.

**Practical Tips**

- **Start Slow**: If new to exercise, begin with low-impact activities (e.g., walking, yoga) to gauge how medications and exercise interact.
- **Consult a Doctor**: Especially for high-intensity sports or if there’s a history of heart issues, diabetes, or other conditions.
- **Timing Matters**: Avoid exercising late if medications cause insomnia. For long-acting stimulants (e.g., Concerta, Vyvanse), effects last 8-12 hours, so morning workouts may align better.
- **Listen to Your Body**: Stop and seek medical advice if you experience unusual symptoms (e.g., palpitations, severe fatigue).

**Additional Notes**

- **Individual Variation**: Responses to medications and exercise vary. Some may feel energized, while others need to adjust timing or intensity.
- **Children vs. Adults**: Children on stimulants can often exercise normally (e.g., sports), but parents should monitor for overexertion. Adults may need to balance exercise with work schedules and medication timing.
- **Recent Research**: As of 2025, no major studies indicate direct conflicts between ADHD medications and exercise in healthy individuals, but ongoing research emphasizes personalized treatment plans.

If you want specifics (e.g., medication side effect profiles, exercise recommendations for a specific age group, or a chart comparing medication effects), let me know! Would you like me to generate a chart showing, for example, common side effects of ADHD medications that might affect exercise?

*Disclaimer: Grok is not a doctor; please consult one. Don't share information that can identify you.*

**Times 7**

- 1. **What is ADHD?**

ADHD, or Attention-Deficit/Hyperactivity Disorder, is a neurodevelopmental condition characterized by persistent patterns of inattention, impulsivity, and/or hyperactivity that are inappropriate for a person's age and interfere with daily functioning. It typically emerges in childhood and can continue into adulthood.

Key features include:

- **Inattention**: Difficulty focusing, staying organized, or completing tasks; easily distracted; forgetfulness.
- **Hyperactivity**: Excessive fidgeting, restlessness, or difficulty staying still (more common in children).
- **Impulsivity**: Acting without thinking, interrupting others, or making hasty decisions.

ADHD is often categorized into three types:

1. **Predominantly Inattentive**: Struggles primarily with attention and organization.
2. **Predominantly Hyperactive-Impulsive**: More issues with hyperactivity and impulsive behavior.
3. **Combined**: A mix of both inattention and hyperactivity-impulsivity.

It’s caused by a mix of genetic, neurological, and environmental factors, with differences in brain structure and function (e.g., dopamine regulation) playing a role. Diagnosis involves clinical evaluation, often using criteria from the DSM-5. Treatment can include behavioral therapy, medication (e.g., stimulants like methylphenidate), and lifestyle adjustments.

If you want more details or have specific questions about ADHD, let me know!

- 1. **What are its symptoms?**

ADHD symptoms vary by individual and can differ depending on the type (inattentive, hyperactive-impulsive, or combined). They generally fall into two main categories: **inattention** and **hyperactivity-impulsivity**. Below is a breakdown of common symptoms, based on clinical criteria like those in the DSM-5, tailored to both children and adults since symptoms can evolve with age.

**Inattention Symptoms**

These involve difficulty sustaining focus and organizing tasks:

- **Difficulty sustaining attention**: Struggling to focus on tasks, conversations, or activities (e.g., zoning out during lectures or meetings).
- **Easily distracted**: Frequently sidetracked by unrelated thoughts or external stimuli (e.g., noises, phone notifications).
- **Forgetfulness**: Often forgetting daily tasks, appointments, or losing items like keys, wallets, or homework.
- **Poor organization**: Trouble planning or completing tasks, leading to messy work or missed deadlines.
- **Avoiding tasks requiring sustained effort**: Procrastinating on or avoiding activities like studying, paperwork, or complex projects.
- **Difficulty following instructions**: Starting tasks but not finishing them or missing steps in multi-step directions.
- **Making careless mistakes**: Overlooking details in schoolwork, job tasks, or chores.

**Hyperactivity-Impulsivity Symptoms**

These involve excessive movement or impulsive actions:

- **Fidgeting or restlessness**: Constantly moving, tapping hands/feet, or feeling unable to sit still (in adults, this may feel like inner restlessness).
- **Excessive talking**: Talking more than is typical, often dominating conversations or speaking out of turn.
- **Difficulty staying seated**: Frequently leaving seats in inappropriate situations (e.g., classrooms, meetings).
- **Running or climbing excessively**: In children, this may involve inappropriate physical activity; in adults, it might manifest as feeling driven or overly busy.
- **Interrupting others**: Blurting out answers, finishing others’ sentences, or intruding on conversations or activities.
- **Acting without thinking**: Making impulsive decisions, like impulsive spending, quitting jobs abruptly, or risky behaviors.
- **Difficulty waiting for turns**: Impatience in lines or conversations, often cutting in.

**Key Notes**

- **Age Differences**: In children, hyperactivity is often more physical (e.g., running around), while in adults, it may appear as restlessness or an inability to relax. Inattention in adults often manifests as trouble with time management or prioritizing tasks.
- **Frequency and Impact**: Symptoms must occur frequently (more than is typical for someone’s age) and significantly impair functioning in at least two settings (e.g., home, school, work, social life).
- **Onset**: Symptoms typically appear before age 12, though diagnosis can happen later, especially in adults where ADHD may have been missed in childhood.
- **Co-occurring Conditions**: ADHD often comes with anxiety, depression, learning disabilities, or other mental health conditions, which can complicate symptoms.

**Examples in Context**

- **Child**: A 9-year-old who can’t sit still in class, frequently loses homework, and interrupts the teacher.
- **Adult**: A 30-year-old who struggles to meet work deadlines, feels constantly overwhelmed by tasks, and impulsively makes big purchases.

If you want specifics, like how symptoms differ by gender, examples in a particular age group, or strategies to manage certain symptoms, let me know!

*Disclaimer: Grok is not a doctor; please consult one. Don't share information that can identify you.*

- 1. **What medications are used for ADHD, and do they conflict with physical exercise?**

Medications for ADHD primarily aim to improve focus, reduce impulsivity, and manage hyperactivity by targeting brain neurotransmitters like dopamine and norepinephrine. Below, I’ll outline the main types of ADHD medications, their mechanisms, and whether they conflict with physical exercise, based on current medical understanding.

### **Medications for ADHD**

1. **Stimulants**
   - **Mechanism**: Increase dopamine and norepinephrine levels in the brain, enhancing attention and self-control.
   - **Common Medications**:
     - **Methylphenidate-based**: Ritalin, Concerta, Daytrana (patch), Focalin, Quillivant XR.
     - **Amphetamine-based**: Adderall, Vyvanse, Dexedrine.
   - **Duration**: Available in short-acting (3-6 hours), intermediate-acting (6-8 hours), or long-acting (8-12+ hours) forms.
   - **Usage**: Most commonly prescribed due to effectiveness in about 70-80% of patients.
   - **Side Effects**: May include reduced appetite, insomnia, increased heart rate, elevated blood pressure, anxiety, or irritability.
2. **Non-Stimulants**
   - **Mechanism**: Work more gradually, often targeting norepinephrine or other pathways without the direct dopamine boost of stimulants.
   - **Common Medications**:
     - **Atomoxetine (Strattera)**: Selective norepinephrine reuptake inhibitor.
     - **Guanfacine (Intuniv)**: Alpha-2 agonist, often used for hyperactivity and impulsivity.
     - **Clonidine (Kapvay)**: Similar to guanfacine, often for calming effects.
     - **Bupropion (Wellbutrin)**: Occasionally used off-label, primarily for adults with ADHD and co-occurring depression.
   - **Usage**: Prescribed when stimulants are ineffective, cause intolerable side effects, or if there’s a risk of substance abuse.
   - **Side Effects**: May include drowsiness, fatigue, nausea, or lowered blood pressure.
3. **Other Medications**
   - Antidepressants (e.g., tricyclics like desipramine) or certain antipsychotics may be used off-label for complex cases, especially with co-occurring conditions.
   - These are less common and typically managed by specialists.

### **Do ADHD Medications Conflict with Physical Exercise?**

Generally, ADHD medications **do not conflict with physical exercise** and can even complement it, as exercise itself boosts dopamine and improves ADHD symptoms. However, there are considerations based on medication type and individual response:

1. **Stimulants and Exercise**
   - **Potential Effects**: Stimulants can increase heart rate and blood pressure, which exercise also elevates. For most people, this isn’t an issue, but those with pre-existing heart conditions (e.g., arrhythmias, hypertension) need medical clearance.
   - **Considerations**:
     - **Cardiovascular Monitoring**: Rarely, stimulants may cause palpitations or irregular heart rhythms during intense exercise. A doctor may recommend an EKG or stress test before starting stimulants, especially for adults or athletes.
     - **Dehydration**: Stimulants can reduce appetite and thirst perception, increasing dehydration risk during exercise. Staying hydrated is critical.
     - **Timing**: Short-acting stimulants wear off faster, so scheduling exercise when medication effects are lower (e.g., late afternoon for morning doses) may reduce strain. Long-acting forms need less timing consideration.
   - **Benefits**: Stimulants can enhance focus during exercise, potentially improving performance in structured activities like sports.
2. **Non-Stimulants and Exercise**
   - **Potential Effects**: Non-stimulants like guanfacine or clonidine may lower blood pressure or cause drowsiness, potentially leading to fatigue during exercise. Atomoxetine has less impact on heart rate but may still cause mild dizziness.
   - **Considerations**:
     - **Fatigue**: If non-stimulants cause tiredness, morning exercise might be better than evening sessions.
     - **Blood Pressure**: Low blood pressure from alpha-2 agonists could cause lightheadedness during intense workouts, so gradual warm-ups are advised.
   - **Benefits**: Non-stimulants are less likely to affect heart rate significantly, making them safer for those with cardiovascular concerns.
3. **General Guidance**
   - **Consult a Doctor**: Before starting or changing an exercise routine, especially intense activities (e.g., competitive sports, heavy weightlifting), consult a healthcare provider to ensure the medication and dose are safe.
   - **Monitor Symptoms**: Watch for signs like excessive fatigue, dizziness, or heart palpitations during exercise, and report them to a doctor.
   - **Hydration and Nutrition**: Both stimulants and exercise can suppress appetite, so ensure adequate calorie and fluid intake to support activity.
   - **Exercise as Therapy**: Regular exercise (e.g., 30 minutes of aerobic activity most days) can reduce ADHD symptoms, potentially allowing lower medication doses in some cases, under medical supervision.

### **Key Takeaways**

- ADHD medications (stimulants and non-stimulants) are generally compatible with exercise, but individual factors like heart health, medication timing, and hydration need attention.
- Stimulants may require more monitoring due to cardiovascular effects, while non-stimulants might cause fatigue or low blood pressure.
- Exercise is highly beneficial for ADHD, often enhancing medication effects by naturally boosting focus and mood.

If you have a specific medication, type of exercise, or health condition in mind, let me know, and I can provide more tailored information!

Disclaimer: Grok is not a doctor; please consult one. Don't share information that can identify you.

**Times 8**

1. **What is ADHD?**

ADHD, or Attention-Deficit/Hyperactivity Disorder, is a neurodevelopmental condition characterized by persistent patterns of inattention, hyperactivity, and/or impulsivity that interfere with daily functioning or development. It typically begins in childhood and can continue into adulthood. Symptoms are grouped into three main types:

1. **Inattentive Type**: Difficulty sustaining attention, being easily distracted, forgetting daily tasks, or struggling with organization (e.g., losing things, missing details in work).
2. **Hyperactive-Impulsive Type**: Excessive fidgeting, restlessness, talking excessively, interrupting others, or acting without thinking.
3. **Combined Type**: A mix of both inattentive and hyperactive-impulsive symptoms.

Symptoms must be present for at least six months, occur in multiple settings (e.g., home, school, work), and be inappropriate for the person’s age or developmental level. ADHD is thought to stem from differences in brain structure and function, particularly in areas regulating attention and impulse control, with genetics playing a significant role.

Diagnosis involves a comprehensive evaluation by a healthcare professional, often including behavioral questionnaires and ruling out other conditions. Treatment typically includes behavioral therapy, medication (like stimulants or non-stimulants), and lifestyle adjustments. About 5-7% of children and 2-5% of adults worldwide are estimated to have ADHD, based on diagnostic criteria. It can impact academic, occupational, and social functioning but can often be managed effectively with tailored strategies.

Disclaimer: Grok is not a doctor; please consult one. Don't share information that can identify you.

1. **What are its symptoms?**

ADHD symptoms vary by individual and can present differently across the three types: inattentive, hyperactive-impulsive, or combined. Below is a breakdown of common symptoms, based on diagnostic criteria like those in the DSM-5:

**Inattentive Type Symptoms**:

- Difficulty sustaining attention on tasks or activities (e.g., trouble focusing during conversations or reading).
- Frequently making careless mistakes in schoolwork, work, or other activities.
- Trouble organizing tasks or managing time (e.g., missing deadlines, messy workspaces).
- Often losing items needed for tasks (e.g., keys, books, phone).
- Being easily distracted by external stimuli or unrelated thoughts.
- Forgetting daily responsibilities (e.g., appointments, paying bills).
- Avoiding or disliking tasks requiring sustained mental effort.
- Appearing not to listen when spoken to directly.

**Hyperactive-Impulsive Type Symptoms**:

- Fidgeting, tapping hands/feet, or squirming in seat.
- Inability to stay seated in situations where it’s expected (e.g., classroom, meetings).
- Running or climbing excessively in inappropriate settings (in children; in adults, this may manifest as restlessness).
- Talking excessively or blurting out answers before questions are completed.
- Difficulty waiting for one’s turn (e.g., interrupting conversations or cutting in line).
- Acting impulsively without considering consequences (e.g., making rash decisions).
- Feeling driven by a motor, unable to relax quietly.

**Combined Type**:

- A mix of symptoms from both categories, meeting criteria for both inattention and hyperactivity-impulsivity.

Symptoms must:

- Persist for at least six months.
- Be inconsistent with the person’s developmental level.
- Occur in multiple settings (e.g., home, school, work).
- Cause significant impairment in social, academic, or occupational functioning.

In children, symptoms are often more noticeable in structured settings like school. In adults, hyperactivity may present as inner restlessness, and inattention might manifest as chronic disorganization or difficulty completing tasks. Symptoms can evolve over time, and not all individuals experience every symptom. Diagnosis requires a professional evaluation to confirm ADHD and rule out other conditions with similar symptoms.

Disclaimer: Grok is not a doctor; please consult one. Don't share information that can identify you.

1. **What medications are used for ADHD, and do they conflict with physical exercise?**

Medications Used for ADHD

Medications for ADHD primarily aim to improve focus, reduce impulsivity, and manage hyperactivity by targeting neurotransmitters like dopamine and norepinephrine. The main categories and commonly prescribed medications include:

1. Stimulants: These are the most common and effective ADHD medications, working by increasing dopamine and norepinephrine levels in the brain.

• Methylphenidate-based: Ritalin, Concerta, Daytrana (patch), Focalin, Quillivant XR, Metadate.

• Amphetamine-based: Adderall, Vyvanse, Dexedrine, Evekeo.

• Effects: Improve attention, reduce impulsivity, and help with task organization. About 70-80% of people with ADHD respond well to stimulants.

2. Non-Stimulants: Used when stimulants are ineffective, cause intolerable side effects, or are not suitable due to other health conditions.

• Atomoxetine (Strattera): A norepinephrine reuptake inhibitor, often used for those with anxiety or substance abuse history.

• Guanfacine (Intuniv) and Clonidine (Kapvay): Alpha-2 agonists, often used to manage hyperactivity and impulsivity, sometimes in combination with stimulants.

• Bupropion (Wellbutrin): An antidepressant sometimes used off-label for ADHD, particularly in adults with co-occurring depression.

• Effects: Slower onset than stimulants (weeks vs. hours) but can provide steady symptom control.

3. Other Medications: Rarely, tricyclic antidepressants or other off-label medications may be prescribed, typically for complex cases or when other treatments fail.

Note: Medication choice depends on age, symptom severity, co-existing conditions (e.g., anxiety, tics), and individual response. Dosages are tailored, and treatment often includes behavioral therapy alongside medication. A healthcare provider must monitor effects and side effects.

Do ADHD Medications Conflict with Physical Exercise?

Generally, ADHD medications do not conflict with physical exercise, and exercise is often encouraged as a complementary strategy for managing ADHD symptoms. However, there are considerations based on medication type, side effects, and individual health:

1. Stimulants:

• Potential Effects on Exercise:

• Increased Heart Rate/Blood Pressure: Stimulants can elevate heart rate and blood pressure, which may be amplified during intense exercise. For most healthy individuals, this isn’t a problem, but those with pre-existing heart conditions (e.g., arrhythmias, hypertension) need medical clearance.

• Reduced Appetite: A common side effect, this may affect energy levels for exercise, especially if caloric intake is low. Timing meals or snacks before workouts can help.

• Dehydration Risk: Stimulants may reduce thirst perception, increasing dehydration risk during exercise. Staying hydrated is critical.

• Anxiety or Jitteriness: High doses may cause feelings of restlessness, which could impact exercise performance or comfort.

• Considerations:

• Most people with ADHD can exercise safely while on stimulants. Aerobic exercise (e.g., running, swimming) may even enhance focus and reduce hyperactivity, complementing medication effects.

• Avoid exercising near the medication’s peak effect if it causes jitteriness. Timing workouts during “off” periods (e.g., before a dose or after it wears off) may help.

• Monitor heart rate during intense exercise, especially for new users or those on higher doses. Consult a doctor if you experience chest pain, palpitations, or dizziness.

2. Non-Stimulants:

• Atomoxetine:

• Effects on Exercise: Less likely to increase heart rate significantly compared to stimulants, but it can cause fatigue or dizziness in some, which may affect exercise stamina. These side effects often diminish over time.

• Considerations: Generally safe for exercise. Hydration and gradual increases in exercise intensity are recommended.

• Guanfacine/Clonidine:

• Effects on Exercise: These can lower blood pressure and cause sedation, potentially reducing exercise endurance or causing dizziness during intense activity.

• Considerations: Schedule exercise when sedation is minimal (e.g., morning if taken at night). Avoid sudden, intense exertion without medical approval.

• Bupropion:

• Effects on Exercise: May increase energy in some, aiding exercise, but can cause agitation or, rarely, seizures at high doses, particularly with intense physical exertion.

• Considerations: Safe for most, but monitor for unusual symptoms during exercise.

3. General Guidelines:

• Consult a Doctor: Before starting an exercise regimen, especially intense activities (e.g., competitive sports, heavy weightlifting), consult a healthcare provider, particularly if you have cardiovascular or other health concerns.

• Monitor Symptoms: Watch for side effects like dizziness, excessive fatigue, or irregular heart rate during exercise, and report them to your doctor.

• Hydration and Nutrition: Exercise can exacerbate medication-related appetite suppression or dehydration. Ensure adequate water and nutrient intake before and after workouts.

• Exercise as a Benefit: Physical activity, especially aerobic exercise, can improve ADHD symptoms by boosting dopamine and norepinephrine naturally, potentially enhancing medication effects. Activities like martial arts, yoga, or team sports may also improve focus and self-regulation.

• Timing: If side effects interfere with exercise, discuss adjusting medication timing or dosage with your doctor. For example, extended-release formulations may provide smoother coverage without peaks that disrupt workouts.

Special Cases:

• Children/Teens: Exercise is highly beneficial for young people with ADHD, but parents should monitor for overexertion or medication-related side effects. Non-competitive activities may be better for some.

• Adults with Co-existing Conditions: Those with heart conditions, high blood pressure, or other health issues should have a thorough medical evaluation before combining ADHD medications with exercise.

• High-Intensity Sports: Athletes on stimulants should be cautious, as these medications are sometimes restricted in competitive sports due to their performance-enhancing potential. Check with sports governing bodies (e.g., WADA) for regulations.

Data Point: Studies suggest exercise can reduce ADHD symptoms by 20-30% in some individuals, making it a valuable adjunct to medication. No large-scale studies indicate significant conflicts between ADHD medications and exercise in healthy individuals, but individual responses vary.

If you have specific concerns about a medication or exercise routine, share details (e.g., medication name, dosage, exercise type), and I can provide more tailored guidance. Always consult your healthcare provider for personalized advice.

Disclaimer: Grok is not a doctor; please consult one. Don't share information that can identify yo

**Times 9**

- 1. **What is ADHD?**

ADHD, or Attention-Deficit/Hyperactivity Disorder, is a neurodevelopmental condition characterized by persistent patterns of inattention, hyperactivity, and/or impulsivity that interfere with daily functioning or development. It typically emerges in childhood and can continue into adulthood. Symptoms vary but often include:

- **Inattention**: Difficulty focusing, staying organized, or completing tasks; easily distracted; forgetfulness in daily activities.
- **Hyperactivity**: Excessive fidgeting, restlessness, or difficulty staying seated when expected.
- **Impulsivity**: Acting without thinking, interrupting others, or struggling to wait for one’s turn.

There are three main types: predominantly inattentive, predominantly hyperactive-impulsive, or combined. Diagnosis is based on clinical criteria, like those in the DSM-5, and often involves input from parents, teachers, or others.

ADHD is thought to stem from differences in brain structure and function, particularly in areas controlling attention and impulse regulation, with genetics playing a significant role. Treatment may include behavioral therapy, medication (like stimulants), and lifestyle adjustments. It affects about 5-7% of children and 2-5% of adults globally, though exact numbers vary by region and diagnostic criteria.

If you want specifics, like symptoms in adults vs. kids or treatment options, let me know!

Disclaimer: Grok is not a doctor; please consult one. Don't share information that can identify you.

- 1. **What are its symptoms?**

ADHD symptoms vary by individual and can differ across age groups and the type of ADHD (inattentive, hyperactive-impulsive, or combined). They generally fall into two main categories: inattention and hyperactivity-impulsivity. Below is a breakdown of common symptoms, based on clinical criteria like those in the DSM-5:

### **Inattention Symptoms**

- Difficulty sustaining attention during tasks or conversations
- Frequently making careless mistakes in work, school, or other activities
- Trouble organizing tasks or managing time (e.g., missing deadlines, losing items)
- Easily distracted by external stimuli or unrelated thoughts
- Forgetfulness in daily activities (e.g., forgetting appointments, chores, or to return calls)
- Avoiding or procrastinating on tasks requiring sustained mental effort
- Frequently losing items like keys, books, or phones
- Struggling to follow through on instructions or complete tasks

### **Hyperactivity-Impulsivity Symptoms**

- Fidgeting, tapping hands/feet, or squirming when seated
- Inability to stay seated in situations where it’s expected (e.g., classroom, meetings)
- Running or climbing in inappropriate settings (more common in children)
- Feeling restless or unable to relax (in adults, often described as inner restlessness)
- Talking excessively or interrupting others during conversations
- Difficulty waiting for one’s turn (e.g., in lines or discussions)
- Acting without thinking (e.g., impulsive decisions, blurting out answers)
- Intruding on others’ activities or conversations

### **Differences by Age**

- **Children**: Hyperactivity is often more visible (e.g., running around, excessive talking). Inattention may show as trouble focusing in school or forgetting homework.
- **Adults**: Hyperactivity often shifts to restlessness or feeling "on edge." Inattention may manifest as chronic disorganization, trouble meeting work deadlines, or difficulty managing household responsibilities.

### **Additional Notes**

- Symptoms must be persistent (lasting at least 6 months), inappropriate for the person’s age, and present in multiple settings (e.g., home, school, work) to meet diagnostic criteria.
- Symptoms can lead to challenges like poor academic/work performance, strained relationships, or low self-esteem.
- Not everyone with ADHD has all symptoms; severity varies widely.

If you want a deeper dive into how symptoms differ by ADHD type, age group, or gender, or how they’re assessed, just ask!

Disclaimer: Grok is not a doctor; please consult one. Don't share information that can identify you.

- 1. **What medications are used for ADHD, and do they conflict with physical exercise?**

### Medications Used for ADHD

Medications for ADHD primarily aim to improve focus, reduce impulsivity, and manage hyperactivity by targeting brain neurotransmitters, especially dopamine and norepinephrine. They fall into two main categories: stimulants and non-stimulants. Below is an overview of commonly used medications, based on current medical guidelines and clinical practice:

#### **Stimulants**

These are the most commonly prescribed and often the most effective for managing ADHD symptoms. They work by increasing dopamine and norepinephrine levels in the brain.

- **Methylphenidate-based**:
  - Ritalin (immediate-release, short-acting)
  - Concerta (extended-release)
  - Focalin (dexmethylphenidate)
  - Daytrana (transdermal patch)
- **Amphetamine-based**:
  - Adderall (mixed amphetamine salts, immediate or extended-release)
  - Vyvanse (lisdexamfetamine, long-acting)
  - Dexedrine (dextroamphetamine)
- **Effects**: Improve attention, reduce impulsivity, and decrease hyperactivity. Onset varies (15-60 minutes for immediate-release; longer for extended-release).
- **Common Side Effects**: Reduced appetite, insomnia, increased heart rate/blood pressure, anxiety, irritability, or headaches.

#### **Non-Stimulants**

Used when stimulants are ineffective, not tolerated, or contraindicated (e.g., due to substance abuse history or certain medical conditions).

- **Atomoxetine (Strattera)**:
  - Selective norepinephrine reuptake inhibitor.
  - Takes 2-6 weeks for full effect.
  - Side effects: Fatigue, nausea, mood swings, or rare liver issues.
- **Guanfacine (Intuniv)** and **Clonidine (Kapvay)**:
  - Alpha-2 agonists, often used in children or as adjuncts to stimulants.
  - Help with hyperactivity and impulsivity; may cause sedation or low blood pressure.
- **Bupropion (Wellbutrin)**:
  - Atypical antidepressant, sometimes used off-label for ADHD.
  - Side effects: Insomnia, agitation, or increased seizure risk.
- **Others**: Tricyclic antidepressants or modafinil may be used in rare cases, but less commonly.

#### **Other Considerations**

- **Dosage/Timing**: Varies by individual, medication type, and formulation (short-acting vs. long-acting). Doctors titrate doses to balance efficacy and side effects.
- **Off-Label Use**: Some medications (e.g., bupropion) are not FDA-approved for ADHD but may be prescribed based on clinical judgment.
- **Monitoring**: Regular check-ins are needed to assess efficacy, side effects, and cardiovascular health (especially with stimulants).

### Do ADHD Medications Conflict with Physical Exercise?

Generally, ADHD medications **do not conflict with physical exercise**, and exercise is often recommended as a complementary strategy for managing ADHD symptoms. However, there are considerations to ensure safety and optimize benefits, particularly due to the physiological effects of these medications:

#### **Stimulants and Exercise**

- **Cardiovascular Effects**:
  - Stimulants can increase heart rate and blood pressure, which may be amplified during intense exercise. For most healthy individuals, this isn’t an issue, but those with pre-existing heart conditions (e.g., arrhythmias, hypertension) need medical clearance before engaging in vigorous exercise.
  - **Risk**: Rare cases of sudden cardiac events have been reported, particularly in individuals with undiagnosed heart issues. A pre-treatment ECG or cardiac evaluation may be recommended for some.
- **Dehydration and Appetite**:
  - Stimulants can reduce appetite and increase body temperature, potentially leading to dehydration or reduced energy reserves during exercise. Staying hydrated and eating adequately before workouts is crucial.
- **Timing**:
  - Short-acting stimulants (e.g., Ritalin) may wear off during the day, potentially affecting exercise performance if taken hours earlier. Long-acting formulations (e.g., Concerta, Vyvanse) provide more consistent coverage.
  - Some people schedule exercise during “off” periods (when medication effects wane) to avoid overstimulation.
- **Positive Effects**:
  - Exercise can enhance the benefits of stimulants by boosting dopamine and norepinephrine naturally, potentially improving mood and focus. Activities like running, swimming, or team sports are often beneficial for ADHD.

#### **Non-Stimulants and Exercise**

- **Atomoxetine**:
  - Less likely to affect heart rate or blood pressure significantly compared to stimulants, making it generally safe for exercise. However, fatigue or sedation in some users could reduce exercise motivation.
- **Guanfacine/Clonidine**:
  - These can lower blood pressure or cause drowsiness, which may affect exercise performance, especially during high-intensity workouts. Gradual warm-ups and monitoring for dizziness are advised.
- **Bupropion**:
  - Generally safe with exercise, but its stimulating effects may increase agitation or heart rate in some cases. Hydration and moderation are key.

#### **General Guidelines for Exercise with ADHD Medications**

- **Consult a Doctor**: Before starting a new exercise regimen, especially intense activities (e.g., competitive sports, heavy weightlifting), discuss with your healthcare provider, particularly if you have cardiovascular risks or are on stimulants.
- **Monitor Symptoms**: Watch for signs of overexertion, such as excessive fatigue, dizziness, or irregular heart rate. Adjust exercise intensity or timing if needed.
- **Stay Hydrated and Nourished**: Stimulants’ appetite suppression can lead to low energy for workouts. Plan meals or snacks around medication schedules to fuel exercise.
- **Exercise as Therapy**: Physical activity (e.g., 30-60 minutes of moderate aerobic exercise) can reduce ADHD symptoms, improve mood, and enhance medication efficacy. Activities like yoga or martial arts may also help with impulse control and focus.
- **Individual Variation**: Some people may feel overstimulated combining stimulants with intense exercise, while others thrive. Listen to your body and adjust as needed.

#### **When to Be Cautious**

- **Pre-existing Conditions**: Heart conditions, high blood pressure, or a history of seizures require careful monitoring and medical guidance.
- **New Medications**: When starting or adjusting ADHD meds, ease into exercise to gauge how your body responds.
- **High-Intensity Sports**: Competitive athletes may need tailored medication schedules to avoid performance issues or side effects during events.

If you have specific details (e.g., which medication you’re considering, type of exercise, or health conditions), I can provide more tailored advice. Would you like me to dig deeper into any aspect, like specific medications, exercise types, or research on ADHD and physical activity?

Disclaimer: Grok is not a doctor; please consult one. Don't share information that can identify you.

**Times 10**

- 1. **What is ADHD?**

ADHD, or Attention-Deficit/Hyperactivity Disorder, is a neurodevelopmental condition characterized by persistent patterns of inattention, impulsivity, and/or hyperactivity that are inappropriate for a person's age and can interfere with daily functioning. It typically begins in childhood and can continue into adulthood. Symptoms are grouped into three main types:

1. **Inattentive Type**: Difficulty focusing, staying organized, or completing tasks; often forgetful or easily distracted.
2. **Hyperactive-Impulsive Type**: Excessive fidgeting, trouble staying still, impulsive decision-making, or interrupting others.
3. **Combined Type**: A mix of inattention and hyperactivity-impulsivity symptoms.

Common signs include trouble concentrating, difficulty following instructions, restlessness, or acting without thinking. It’s diagnosed through clinical evaluation, often involving input from parents, teachers, or others, and must meet criteria outlined in diagnostic manuals like the DSM-5. ADHD is thought to stem from differences in brain structure and function, particularly in areas controlling attention and impulse regulation, with genetics playing a significant role.

Management includes behavioral therapy, lifestyle changes, and sometimes medication like stimulants (e.g., methylphenidate) or non-stimulants. Support strategies, like structured routines or accommodations at school/work, can also help. It affects about 5-7% of children and 2-5% of adults globally, with varying severity.

- 1. **What are its symptoms?**

ADHD symptoms vary by individual and can present differently depending on age, environment, and the specific type of ADHD (inattentive, hyperactive-impulsive, or combined). Below is a breakdown of the core symptoms, based on diagnostic criteria like those in the DSM-5:

### **Inattentive Symptoms**

These are more common in the inattentive type of ADHD and may include:

- Difficulty sustaining attention in tasks or play.
- Frequently making careless mistakes in schoolwork or other activities.
- Trouble organizing tasks or managing time effectively.
- Often losing items needed for tasks (e.g., keys, books, phone).
- Being easily distracted by unrelated thoughts or external stimuli.
- Forgetfulness in daily activities, like forgetting appointments or chores.
- Avoiding or disliking tasks requiring sustained mental effort (e.g., homework or paperwork).
- Difficulty following through on instructions or completing tasks.
- Seeming not to listen when spoken to directly.

### **Hyperactive-Impulsive Symptoms**

These are more prominent in the hyperactive-impulsive type of ADHD and may include:

- Fidgeting, tapping hands or feet, or squirming in seats.
- Inability to stay seated in situations where it’s expected (e.g., in class or meetings).
- Running or climbing in inappropriate settings (more common in children).
- Trouble engaging in quiet activities.
- Acting as if “driven by a motor,” with excessive energy or restlessness.
- Talking excessively or interrupting conversations.
- Blurting out answers before questions are completed.
- Difficulty waiting for one’s turn (e.g., in lines or conversations).
- Intruding on others’ activities or conversations.

### **Combined Type**

Individuals with combined ADHD exhibit a mix of both inattentive and hyperactive-impulsive symptoms, with at least six symptoms from each category (or fewer in adults, per DSM-5 criteria).

### **Additional Notes**

- **Age and Context**: Symptoms must be inappropriate for the person’s developmental stage and present in multiple settings (e.g., home, school, work). In children, symptoms are often more noticeable in structured environments. In adults, hyperactivity may manifest as restlessness or trouble relaxing.
- **Duration and Impact**: Symptoms must persist for at least six months and negatively affect social, academic, or occupational functioning.
- **Adult-Specific Presentation**: Adults may show subtler symptoms, like chronic disorganization, impulsivity in decision-making, or difficulty managing responsibilities.
- **Co-occurring Issues**: ADHD often comes with challenges like anxiety, depression, or learning difficulties, which can overlap with or amplify symptoms.

Diagnosis requires a professional evaluation, as symptoms can resemble other conditions (e.g., anxiety, thyroid issues). If you’re seeking a visual representation of symptom frequency or distribution, I can generate a chart if you provide specific data or preferences.

Disclaimer: Grok is not a doctor; please consult one. Don't share information that can identify you.

- 1. **What medications are used for ADHD, and do they conflict with physical exercise?**

**Medications Used for ADHD**

Medications for ADHD primarily aim to improve attention, reduce impulsivity, and/or manage hyperactivity by targeting neurotransmitters like dopamine and norepinephrine. They are broadly categorized into **stimulants** and **non-stimulants**. Below is an overview of commonly prescribed medications:

**Stimulant Medications**

Stimulants are the most common and effective treatment for ADHD, working for about 70-80% of patients. They increase dopamine and norepinephrine levels in the brain. Common stimulants include:

- **Methylphenidate-based**:
  - Ritalin, Concerta, Daytrana (patch), Metadate, Quillivant XR, Focalin (dexmethylphenidate).
- **Amphetamine-based**:
  - Adderall (mixed amphetamine salts), Vyvanse (lisdexamfetamine), Dexedrine (dextroamphetamine).
- **Delivery Variations**: Available in short-acting (3-6 hours), intermediate-acting (6-8 hours), or long-acting (8-12+ hours) forms, depending on the formulation.

**Non-Stimulant Medications**

Non-stimulants are often used when stimulants are ineffective, cause intolerable side effects, or are contraindicated. They may take longer to show effects but can be effective for some individuals:

- **Atomoxetine (Strattera)**: A norepinephrine reuptake inhibitor, often used for ADHD with co-occurring anxiety.
- **Guanfacine (Intuniv, Tenex)**: An alpha-2 adrenergic agonist, often used to reduce hyperactivity and impulsivity.
- **Clonidine (Kapvay)**: Another alpha-2 agonist, often used for hyperactivity or as an adjunct to stimulants.
- **Bupropion (Wellbutrin)**: An antidepressant sometimes used off-label for ADHD, particularly in adults.
- **Viloxazine (Qelbree)**: A newer non-stimulant approved for ADHD, targeting norepinephrine modulation.

**Other Considerations**

- **Off-Label Use**: Medications like certain antidepressants (e.g., tricyclics) may be used in specific cases, particularly for adults with co-occurring conditions.
- **Combination Therapy**: Some patients use a stimulant and a non-stimulant together for better symptom control.
- **Individual Response**: Effectiveness and side effects vary, so treatment is tailored through trial and monitoring by a healthcare provider.

**Do ADHD Medications Conflict with Physical Exercise?**

Generally, ADHD medications **do not conflict with physical exercise** and may even enhance performance in some cases due to improved focus and energy regulation. However, there are considerations to ensure safety and compatibility:

**Stimulants and Exercise**

- **Effects on Exercise**:
  - Stimulants can increase heart rate and blood pressure, which may amplify during physical activity. For most healthy individuals, this isn’t an issue, but those with pre-existing heart conditions (e.g., arrhythmias, hypertension) need medical clearance before combining stimulants with intense exercise.
  - Improved focus from stimulants may enhance exercise performance, particularly for tasks requiring coordination or sustained effort (e.g., team sports, workouts).
  - Some individuals report reduced appetite (a common side effect), which could affect energy levels for exercise if nutrition isn’t managed.
- **Potential Concerns**:
  - **Dehydration**: Stimulants can reduce thirst perception, increasing dehydration risk during exercise, especially in hot environments. Staying hydrated is critical.
  - **Overheating**: Stimulants may slightly impair temperature regulation, so precautions (e.g., avoiding intense exercise in extreme heat) are advised.
  - **Sleep Disruption**: Stimulants can interfere with sleep if taken late, which may impact recovery from exercise. Timing doses earlier in the day can help.
- **Precautions**:
  - Consult a doctor before starting exercise regimens, especially for high-intensity activities or if there’s a history of cardiovascular issues.
  - Monitor heart rate during exercise, especially if new to stimulants or increasing exercise intensity.
  - Ensure proper nutrition and hydration to counter appetite suppression and dehydration risks.

**Non-Stimulants and Exercise**

- **Effects on Exercise**:
  - Non-stimulants like atomoxetine, guanfacine, or clonidine generally have less impact on heart rate and blood pressure compared to stimulants, making them less likely to affect exercise directly.
  - Guanfacine and clonidine may cause sedation or fatigue in some, which could reduce exercise motivation or performance, particularly when starting treatment.
  - Atomoxetine may improve focus for exercise without the cardiovascular effects of stimulants.
- **Potential Concerns**:
  - **Fatigue**: Sedation from guanfacine or clonidine may make early morning or high-energy workouts harder. Adjusting dose timing (e.g., evening doses) can help.
  - **Blood Pressure**: Guanfacine and clonidine can lower blood pressure, which might cause dizziness during intense exercise, especially if dehydrated.
- **Precautions**:
  - Gradually increase exercise intensity to assess tolerance, especially with sedating medications.
  - Monitor for dizziness or fatigue and adjust exercise timing if needed.
  - Coordinate with a healthcare provider to optimize medication schedules around exercise routines.

**General Recommendations**

- **Medical Guidance**: Always consult a healthcare provider before combining ADHD medications with exercise, especially for competitive sports or intense training. Pre-exercise cardiac screening may be recommended for stimulants, as rare cases of sudden cardiac events have been reported in those with undiagnosed heart conditions.
- **Timing**: Schedule exercise when medication effects are stable (e.g., 1-2 hours after a dose for stimulants) to maximize focus benefits and minimize side effects like fatigue or elevated heart rate.
- **Hydration and Nutrition**: Counter appetite suppression and dehydration risks by eating balanced meals and drinking water before, during, and after exercise.
- **Listen to Your Body**: Stop exercising if you experience unusual symptoms like chest pain, extreme fatigue, or dizziness, and seek medical advice.
- **Exercise as a Complement**: Regular physical activity can itself help manage ADHD symptoms by boosting dopamine and improving mood, focus, and impulse control, often complementing medication effects.

**Additional Notes**

- **Individual Variation**: Responses to medications and exercise vary. Some may find stimulants enhance exercise performance, while others may need to adjust dose or timing to avoid side effects.
- **Children vs. Adults**: Children on ADHD medications may need closer monitoring during sports, as their bodies are still developing. Adults may notice more subtle effects, like improved workout consistency due to better focus.
- **Research Gaps**: While studies (e.g., from the American Academy of Pediatrics) show no significant conflict between ADHD medications and exercise for most healthy individuals, data on high-intensity or competitive sports is limited, so caution is advised.

If you’d like a chart comparing side effects of specific ADHD medications or their impact on exercise (e.g., heart rate changes), let me know what data or preferences you have, and I can generate one. If you have a specific medication or exercise type in mind, I can tailor the response further.

*Disclaimer: Grok is not a doctor; please consult one. Don't share information that can identify you.*

# **S6. Prompts and Generated Responses from DeepSeek V3, 15:08, 07/08/2025**

**Times 1**

**Understanding ADHD: Symptoms, Causes, and Treatment**

- 1. **What is ADHD?**

ADHD (Attention-Deficit/Hyperactivity Disorder) is a neurodevelopmental disorder that affects both children and adults. It is characterized by persistent patterns of inattention, hyperactivity, and impulsivity that interfere with daily functioning or development.

**Core Symptoms of ADHD:**

ADHD symptoms are divided into two main categories:

1. **Inattention:**
   - Difficulty sustaining focus on tasks or activities.
   - Easily distracted by unrelated thoughts or stimuli.
   - Trouble organizing tasks and activities.
   - Frequently losing things (e.g., keys, phones, homework).
   - Forgetfulness in daily activities (e.g., missing appointments).
   - Avoiding or disliking tasks that require sustained mental effort.
2. **Hyperactivity and Impulsivity:**
   - Fidgeting or tapping hands/feet.
   - Difficulty staying seated (e.g., in classrooms or meetings).
   - Excessive talking or interrupting others.
   - Acting without thinking (e.g., blurting out answers, taking risks).
   - Difficulty waiting for turns (e.g., in conversations or queues).
   - Feeling restless or "on the go" constantly.

**Types of ADHD:**

Based on symptoms, ADHD is classified into three types:

1. **Predominantly Inattentive Presentation** – Mainly struggles with focus.
2. **Predominantly Hyperactive-Impulsive Presentation** – Mainly struggles with impulsivity and hyperactivity.
3. **Combined Presentation** – Symptoms of both inattention and hyperactivity-impulsivity.

**Causes of ADHD:**

The exact cause is unknown, but research suggests a combination of factors:

- **Genetics** (runs in families).
- **Brain structure & function** (differences in dopamine and norepinephrine systems).
- **Environmental factors** (premature birth, low birth weight, exposure to toxins like lead).
- **Prenatal risks** (e.g., smoking/alcohol use during pregnancy).

**Diagnosis:**

There’s no single test—diagnosis involves:

- Clinical evaluation by a psychiatrist, psychologist, or neurologist.
- Symptom checklists from parents, teachers, or self-reports (for adults).
- Ruling out other conditions (e.g., anxiety, learning disabilities, sleep disorders).

**Treatment:**

ADHD is manageable with a combination of:

- **Medication** (stimulants like Adderall or Ritalin; non-stimulants like Strattera).
- **Behavioral therapy** (CBT, parent training for kids, organizational skills training).
- **Lifestyle changes** (exercise, structured routines, mindfulness techniques).
- **Educational/workplace accommodations** (extra time on tests, flexible schedules).

**ADHD in Adults:**

Often undiagnosed in childhood, adults may experience:

- Chronic procrastination, disorganization, and time-management issues.
- Impulsive decisions (e.g., job changes, spending habits).
- Emotional dysregulation (quick frustration or mood swings).
- Relationship challenges due to forgetfulness or interrupting.

**Myths vs. Facts:**

- *Myth:* ADHD is just laziness or bad parenting.
  *Fact:* It’s a brain-based condition with biological roots.
- *Myth:* Only kids have ADHD.
  *Fact:* Many adults have it, often undiagnosed.
- *Myth:* Medication is the only treatment.
  *Fact:* Therapy and lifestyle changes are also key.

**Support & Resources:**

- **CHADD** (Children and Adults with ADHD) – [chadd.org](https://chadd.org/)
- **ADDitude Magazine** – [additudemag.com](https://www.additudemag.com/)
- **Professional help** (psychiatrists, ADHD coaches).

If you suspect ADHD in yourself or a loved one, a healthcare provider can guide assessment and management. Early intervention improves outcomes significantly!

- 1. **What are its symptoms?**

ADHD symptoms are divided into two main categories: **inattention** and **hyperactivity-impulsivity**. Depending on the subtype, a person may show more of one set of symptoms than the other.

**1. Inattention Symptoms**

(Common in **Predominantly Inattentive Presentation**)

- **Difficulty sustaining attention** (e.g., loses focus during tasks, lectures, or conversations).
- **Careless mistakes** in school/work (overlooks details, rushes through tasks).
- **Avoiding tasks requiring mental effort** (e.g., homework, paperwork, long reports).
- **Poor organization** (misses deadlines, messy workspaces, struggles with time management).
- **Easily distracted** by unrelated thoughts or external stimuli (e.g., background noises).
- **Forgetfulness** in daily activities (loses keys, phones, forgets appointments).
- **Doesn’t seem to listen** when spoken to directly (mind seems elsewhere).
- **Difficulty following instructions** (starts tasks but gets sidetracked).

**2. Hyperactivity-Impulsivity Symptoms**

(Common in **Predominantly Hyperactive-Impulsive Presentation**)
**Hyperactivity:**

- **Fidgeting or squirming** (taps hands/feet, can’t sit still).
- **Leaving seat when expected to stay seated** (e.g., in classrooms, meetings).
- **Running or climbing excessively** (in kids; adults may feel restless).
- **Unable to play/engage quietly** (always "on the go").
- **Excessive talking** (talks nonstop, dominates conversations).

**Impulsivity:**

- **Blurting out answers** before questions are finished.
- **Interrupting or intruding** on others (butts into conversations/games).
- **Difficulty waiting turns** (impatient in lines, traffic, or conversations).
- **Acts without thinking** (takes risks, makes impulsive decisions).

**Combined Presentation**

If a person has **6+ inattention symptoms AND 6+ hyperactivity-impulsivity symptoms** (for children; 5+ for adults), they may have **Combined Type ADHD**.

**ADHD Symptoms in Adults**

While hyperactivity often lessens with age, adults may experience:

- **Chronic procrastination** (delays tasks until the last minute).
- **Disorganization** (cluttered home/workspace, missed deadlines).
- **Impulsivity** (overspending, reckless driving, frequent job changes).
- **Emotional dysregulation** (quick temper, mood swings).
- **Forgetfulness** (misses appointments, loses items).
- **Struggles with focus** (zones out during conversations, drifts during reading).

**When to Seek Help**

If these symptoms:

- **Persist for 6+ months** (in kids) or cause long-term issues (in adults).
- **Occur in multiple settings** (e.g., school, work, home).
- **Interfere with daily life** (academics, relationships, job performance).

A diagnosis from a **psychologist, psychiatrist, or neurologist** can clarify whether it’s ADHD or another condition (e.g., anxiety, sleep disorders).

Would you like details on **diagnosis, coping strategies, or treatment options**?

- 1. **What medications are used for ADHD, and do they conflict with physical exercise?**

**Medications for ADHD**

ADHD medications are generally divided into **stimulants** and **non-stimulants**, with some additional options for specific cases. Here’s a breakdown:

**1. Stimulants (Most Common)**

These increase dopamine and norepinephrine in the brain, improving focus and impulse control.

- **Methylphenidate-based**
  - Ritalin (short-acting)
  - Concerta, Daytrana (long-acting)
  - Focalin (dexmethylphenidate)
- **Amphetamine-based**
  - Adderall (mixed amphetamine salts)
  - Vyvanse (lisdexamfetamine, a prodrug with smoother effects)
  - Dexedrine (dextroamphetamine)

**Side Effects:**

- Increased heart rate, blood pressure
- Decreased appetite
- Insomnia (if taken too late)
- Possible jitteriness or anxiety

**2. Non-Stimulants (Alternative if Stimulants Aren’t Tolerated)**

- **Atomoxetine (Strattera)** – Increases norepinephrine; good for anxiety-prone individuals.
- **Guanfacine (Intuniv)** & **Clonidine (Kapvay)** – Originally blood pressure meds; help with impulsivity and emotional regulation.
- **Bupropion (Wellbutrin)** – An antidepressant sometimes used off-label for ADHD.

**Side Effects:**

- Fatigue (especially early on)
- Stomach upset
- Dry mouth

**3. Other/Off-Label Options**

- **Modafinil (Provigil)** – For excessive sleepiness + ADHD.
- **Antidepressants (e.g., TCAs)** – Rarely, if other meds fail.

**Do ADHD Medications Conflict with Exercise?**

Most ADHD meds **do not** prevent exercise, but there are **key considerations**:

**1. Stimulants & Exercise**

- **Heart Rate & Blood Pressure:** Stimulants increase both, so intense exercise (e.g., heavy weightlifting, HIIT) may feel harder.
  - **Risk:** If you have an undiagnosed heart condition, consult a doctor.
  - **Solution:** Monitor exertion; stay hydrated.
- **Dehydration Risk:** Stimulants can reduce thirst perception.
  - **Fix:** Drink water before/during workouts.
- **Crash After Effects:** Post-workout, meds may wear off faster due to increased metabolism.
  - **Fix:** Time workouts before meds peak or as they taper.

**2. Non-Stimulants & Exercise**

- **Strattera (Atomoxetine):** May cause fatigue early on; light exercise can help.
- **Intuniv/Kapvay:** Can lower blood pressure → dizziness during sudden movements.
  - **Fix:** Avoid rapid position changes (e.g., burpees).

**3. General Tips for Exercising on ADHD Meds**

**Best Workouts:**

- Moderate cardio (jogging, swimming, cycling)
- Yoga/mindful movement (helps with co-regulation)
- Strength training (but avoid extreme heavy lifting if on stimulants)

**Caution With:**

- Extreme endurance sports (marathons, ultra-training)
- High-intensity interval training (HIIT) if prone to anxiety
- Heat exposure (stimulants + hot yoga/saunas = dehydration risk)

**Final Verdict**

- **Most people can exercise safely on ADHD meds** with minor adjustments.
- **Stimulants** require monitoring of heart rate/hydration.
- **Non-stimulants** may cause fatigue or dizziness initially.
- **Listen to your body**—if something feels off, modify intensity.

Would you like advice on **specific workout routines** or **managing meds around exercise schedules**?

**Times 2**

1. **What is ADHD?**

**ADHD (Attention-Deficit/Hyperactivity Disorder)** is a neurodevelopmental disorder characterized by persistent patterns of **inattention**, **hyperactivity**, and/or **impulsivity** that interfere with daily functioning or development. It begins in childhood but often continues into adulthood, affecting work, relationships, and self-esteem.

**Core Features of ADHD**

**1. Inattention**

- Difficulty sustaining focus (easily distracted)
- Frequent careless mistakes (school, work, or daily tasks)
- Avoidance of tasks requiring prolonged mental effort
- Poor organization (missed deadlines, messy spaces)
- Forgetfulness (losing items, missing appointments)
- Often seems "not listening" when spoken to directly

**2. Hyperactivity** (more obvious in children)

- Fidgeting, tapping, or squirming
- Inability to stay seated (e.g., in classrooms or meetings)
- Excessive running/climbing (in kids) or restlessness (in adults)
- Talking excessively or interrupting others
- Difficulty engaging quietly in activities

**3. Impulsivity**

- Blurting out answers before questions are finished
- Impatience (struggling to wait in line or take turns)
- Interrupting conversations or intruding on others
- Making impulsive decisions (e.g., spending, quitting jobs)

**Types of ADHD**

1. **Predominantly Inattentive Presentation** (formerly "ADD")
   - Mainly struggles with focus, organization, and forgetfulness.
   - Often overlooked because hyperactivity isn’t obvious.
2. **Predominantly Hyperactive-Impulsive Presentation**
   - More impulsive actions and restless energy.
   - Common in younger children.
3. **Combined Presentation**
   - Symptoms of both inattention *and* hyperactivity-impulsivity.
   - Most diagnosed type.

**Causes & Risk Factors**

- **Genetics:** Strong hereditary component (~75% heritability).
- **Brain Differences:** Lower activity in prefrontal cortex (area for focus/impulse control).
- **Neurochemical:** Imbalances in dopamine and norepinephrine.
- **Environmental:** Premature birth, prenatal exposure to toxins (e.g., alcohol, smoking), or brain injuries.

**ADHD in Adults**

Many adults are undiagnosed, with symptoms like:

- Chronic procrastination
- Disorganization (cluttered home/workspace)
- Impulsive decisions (relationships, finances)
- Emotional dysregulation (quick frustration)
- Time blindness (always late, poor time estimation)

**Diagnosis**

No single test—diagnosis involves:

1. **Clinical evaluation** by a psychologist/psychiatrist.
2. **Symptom checklists** from childhood/adolescence.
3. **Rule-outs** for similar conditions (anxiety, depression, sleep disorders).

**Treatment Options**

1. **Medication**
   - *Stimulants* (e.g., Adderall, Ritalin, Vyvanse) – Boost focus.
   - *Non-stimulants* (e.g., Strattera, Intuniv) – For those who can’t tolerate stimulants.
2. **Therapy**
   - CBT (Cognitive Behavioral Therapy) for coping strategies.
   - Coaching (for organization/time management).
3. **Lifestyle Adjustments**
   - Exercise (improves focus and mood).
   - Structured routines, reminders, and organizational tools.

**Myths vs. Facts**

**Myth:** ADHD is just laziness or bad parenting.
**Fact:** It’s a brain-based condition with biological roots.

**Myth:** Only hyperactive boys have ADHD.
**Fact:** Girls and adults often have inattentive ADHD, missed for years.

**Myth:** ADHD is outgrown after childhood.
 **Fact:** ~60% of kids with ADHD continue to have symptoms as adults.

**Why It Matters**

Untreated ADHD can lead to:

- Academic/job struggles
- Low self-esteem
- Relationship conflicts
- Higher risk of anxiety, depression, or substance use

Early diagnosis and management (meds + therapy + lifestyle) can dramatically improve quality of life.

**Need clarification or personal insights? Ask away!**

1. **What are its symptoms?**

**ADHD Symptoms: Inattention, Hyperactivity & Impulsivity**

ADHD symptoms fall into **two core categories** and vary by age/subtype. Here’s a detailed breakdown:

**1. Inattention Symptoms**

*(Must persist for ≥6 months and disrupt daily life)*

- **Easily distracted** – Struggles to focus on tasks (e.g., reading, conversations).
- **Careless mistakes** – Overlooks details in schoolwork or job tasks.
- **Avoids sustained effort** – Procrastinates on tasks requiring focus (taxes, reports).
- **Poor organization** – Messy spaces, missed deadlines, chronic lateness.
- **Forgetfulness** – Loses keys/wallets/phones; forgets appointments.
- **Daydreaming** – Seems "zoned out" or not listening when spoken to.
- **Difficulty following instructions** – Starts tasks but gets sidetracked.

**Example (Adult):**
*"I open my laptop to work, but within minutes I’m checking emails, then my phone, then researching random topics—and nothing gets done."*

**2. Hyperactivity-Impulsivity Symptoms**

*(More obvious in children; adults may internalize restlessness)*

**Hyperactivity:**

- **Fidgeting** – Tapping feet, clicking pens, shifting in seats.
- **Leaving seats** – Gets up during meetings/meals (kids may run/climb inappropriately).
- **Excessive talking** – Dominates conversations, interrupts others.
- **"On-the-go" energy** – Feels driven like a "motor" (kids) or restless (adults).

**Impulsivity:**

- **Blurting out answers** – Interrupts questions or finishes others’ sentences.
- **Impatience** – Struggles waiting in lines or taking turns.
- **Risky behaviors** – Speeding, impulsive spending, abrupt job changes.
- **Emotional outbursts** – Quick frustration or anger (then rapid regret).

**Example (Child):**
*"My kid can’t sit through dinner—they’re up, down, talking nonstop, and grabbing things off the table."*

**How Symptoms Differ by Age**

| **Children** | **Adults** |
| --- | --- |
| Running/climbing excessively | Internal restlessness (e.g., leg bouncing) |
| Loud, impulsive play | Impulsive decisions (e.g., quitting jobs) |
| School struggles (forgetful, disorganized) | Work deadlines missed, cluttered home |
| Teacher reports focus issues | Self-critical about procrastination |

**ADHD Subtypes**

1. **Inattentive Type (formerly ADD):**
   - Quiet, daydreamy, "spacey."
   - Often missed in girls/women.
2. **Hyperactive-Impulsive Type:**
   - Disruptive, energetic, impulsive.
   - More commonly diagnosed in boys.
3. **Combined Type:**
   - Mix of both inattention and hyperactivity.
   - Most diagnosed subtype.

**When to Seek Help**

Consult a doctor if:
Symptoms persist in **multiple settings** (home, work, school).
They’ve lasted **≥6 months** and began in childhood (even if undiagnosed).
They cause **significant distress** (failed relationships, job loss, low self-esteem).

**Note:** Anxiety, depression, or sleep disorders can mimic ADHD—a professional evaluation is key.

**Real-Life Impact**

- **Kids:** Homework battles, teacher complaints, social struggles.
- **Adults:** Job instability, financial chaos, "Why can’t I adult?!" frustration.
- **Emotional:** Shame ("I’m lazy"), anxiety from unmet responsibilities.

**Good news:** ADHD is manageable with **medication, therapy, and coping strategies**.

Need help identifying specific symptoms in yourself or a loved one? Ask for details!

1. **What medications are used for ADHD, and do they conflict with physical exercise?**

**ADHD Medications & Exercise: A Complete Guide**

ADHD medications are highly effective but can interact with physical activity. Here’s what you need to know:

**1. Stimulant Medications (Most Common)**

**Examples:**

- **Methylphenidate:** Ritalin (short-acting), Concerta (long-acting)
- **Amphetamines:** Adderall (mixed salts), Vyvanse (lisdexamfetamine), Dexedrine

**How They Work:**
Boost dopamine/norepinephrine to improve focus and impulse control.

**Exercise Interactions:**
**Benefits:**

- Enhanced focus during workouts (e.g., better gym form or running pace).
- Some athletes use them *strategically* for endurance (though this is controversial).

**Risks:**

- **Increased heart rate & blood pressure** → Caution with intense cardio/weightlifting.
- **Dehydration** (stimulants suppress thirst).
- **Overheating** (reduced sweating in some).
- **Crash risk** post-workout as meds wear off.

**Safety Tips:**

- Monitor heart rate (keep it in a safe zone).
- Hydrate *before* feeling thirsty.
- Avoid extreme heat (hot yoga, saunas).
- Time workouts before meds peak or as they taper.

**2. Non-Stimulant Medications**

**Examples:**

- **Atomoxetine (Strattera):** Norepinephrine reuptake inhibitor.
- **Guanfacine (Intuniv)/Clonidine (Kapvay):** Originally for high blood pressure.

**Exercise Interactions:**
**Benefits:**

- No cardiovascular overstimulation (safer for athletes).
- Strattera may improve mood/stamina over time.

**Risks:**

- **Fatigue** (especially when starting Strattera).
- **Dizziness** (guanfacine/clonidine can lower BP).

**Safety Tips:**

- Start with low-intensity exercise (walking, yoga).
- Rise slowly to avoid dizziness (prevents falls).

**3. Off-Label/Other Options**

- **Bupropion (Wellbutrin):** Can help ADHD + depression but may increase seizure risk in extreme endurance sports.
- **Modafinil (Provigil):** Used for ADHD + sleep disorders; monitor hydration.

**Exercise Recommendations by Medication Type**

| **Med Type** | **Best Exercises** | **Avoid/Caution** |
| --- | --- | --- |
| **Stimulants** | Moderate cardio (jogging, swimming), strength training (moderate weights) | HIIT, competitive sports in heat |
| **Strattera** | Yoga, walking, gradual intensity increases | Heavy lifting (if fatigued) |
| **Guanfacine** | Low-impact (cycling, Pilates) | Rapid position changes (burpees) |

**Key Takeaways**

1. **Stimulants + Exercise:** Generally safe but require hydration/HR monitoring.
2. **Non-Stimulants:** Fewer heart risks but may cause fatigue/dizziness.
3. **Listen to Your Body:** Adjust intensity if you feel jittery, dizzy, or exhausted.
4. **Elite Athletes:** Some use ADHD meds *strategically* (e.g., cyclists), but this risks dependency and requires medical oversight.

**Final Advice:**

- **For cardio lovers:** Check BP/HR periodically.
- **For weightlifters:** Avoid max lifts on stimulants (spikes BP).
- **For yogis:** Guanfacine may cause dizziness in inversions.

Need help tailoring exercise to your meds? Ask for a personalized plan!

**Times 3**

1. **What is ADHD?**

**ADHD (Attention-Deficit/Hyperactivity Disorder)** is a neurodevelopmental condition that affects brain structure and function, particularly in areas responsible for attention, impulse control, and executive functioning. It's not simply a behavioral issue or lack of willpower - it's a real neurological difference that impacts approximately 5-10% of children and 2-5% of adults worldwide.

Core Characteristics:

1. **Executive Dysfunction**
   - Impaired working memory (forgetfulness)
   - Difficulty with planning/organization
   - Poor time management ("time blindness")
   - Trouble initiating or switching tasks
2. **Regulation Challenges**
   - Emotional dysregulation (intense emotions)
   - Impulse control difficulties
   - Hyperfocus (extreme concentration on interesting tasks)
3. **Neurological Basis**
   - Differences in prefrontal cortex development
   - Dopamine/norepinephrine system irregularities
   - Often runs in families (highly heritable)

Modern Understanding:

- Now recognized as a **lifespan condition** (not just childhood)
- Presents differently across genders (women often underdiagnosed)
- Frequently co-occurs with:
  • Learning disabilities (30-50%)
  • Anxiety/depression
  • Sleep disorders

Diagnosis Requires:

- Symptoms before age 12
- Impairment in multiple settings
- Ruling out other conditions (e.g., thyroid issues, trauma)

Treatment Approaches:

- **Medication** (stimulants/non-stimulants)
- **Behavioral therapies** (CBT, coaching)
- **Accommodations** (work/school supports)
- **Lifestyle adaptations** (exercise, sleep hygiene)

Current research emphasizes ADHD as a **difference in brain wiring** that comes with both challenges and potential strengths (creativity, resilience, hyperfocus abilities). Proper management focuses on working with one's neurology rather than "fixing" it.

Would you like more details on any particular aspect?

1. **What are its symptoms?**

**ADHD Symptoms Explained (Beyond the Stereotypes)**

ADHD symptoms manifest across three core domains, but their presentation varies dramatically by age, gender, and individual brain wiring. Here's a nuanced breakdown:

**1. Executive Dysfunction Symptoms**

*The hidden struggles of ADHD*

- **Task Initiation Paralysis**: Body "freezes" when starting boring tasks (even when wanting to)
- **Working Memory Gaps**: Forgetting what you're doing mid-task (walking into rooms and blanking)
- **Time Blindness**: Chronic lateness despite efforts (under/overestimating time required)
- **Disorganization Chaos**: "Piles system" organization, important items buried in plain sight
- **Hyperfocus Episodes**: Hours spent on fascinating tasks while ignoring basic needs (eating, bathroom)

**2. Emotional Regulation Symptoms**

*The overlooked emotional rollercoaster*

- **Rejection Sensitivity**: Extreme emotional pain from perceived criticism
- **Emotional Tsunamis**: Quick bursts of intense anger/frustration that fade rapidly
- **Reward Deficiency**: Constant craving for novelty/excitement to feel "alive"
- **Interest-Based Nervous System**: Only motivated by curiosity/urgency (not importance)

**3. Physical Manifestations**

*How ADHD lives in the body*

- **Restless Body Syndrome**: Constant fidgeting, leg bouncing, or skin picking
- **Sensory Seeking/Avoidance**: Craving intense stimulation or overwhelmed by textures/sounds
- **Sleep Inertia**: Extreme difficulty waking up, "sleep drunkenness"
- **Movement Coordination**: Clumsiness, frequent minor accidents

**Gender Differences**

*Why women are often missed*

- **Female Presentation**: More internal restlessness, chronic daydreaming, people-pleasing
- **Male Presentation**: More externalized hyperactivity, physical impulsivity
- **Masking**: Compensating with perfectionism, overachieving (then burning out)

**Life Stage Variations**

*How symptoms evolve*

- **Childhood**: Physical hyperactivity, classroom disruptions
- **Teens**: Risky behaviors, academic struggles despite intelligence
- **Adults**: Job instability, relationship strain, "parenting while ADHD"

**The ADHD Paradox**

*Contradictory symptoms that confuse outsiders*

- Can hyperfocus for hours but can't pay bills on time
- Remembers obscure facts but forgets appointments
- Excellent in crises but struggles with daily tasks
- Deeply creative but organizationally challenged

**Key Insight:** ADHD isn't an attention deficit, but an *attention regulation* disorder. The brain struggles to control what it pays attention to, not with attention itself.

Would you like me to focus on specific symptom clusters (e.g., workplace manifestations, relationship impacts) or provide more details about how these interact with co-occurring conditions?

1. **What medications are used for ADHD, and do they conflict with physical exercise?**

Here's a comprehensive yet streamlined breakdown of ADHD medications and their interaction with exercise:

**ADHD Medication Classes & Exercise Considerations**

**1. Stimulants (First-Line Treatment)**
*Examples:*

- **Methylphenidates**: Ritalin (short), Concerta/Focalin (long)
- **Amphetamines**: Adderall (mixed), Vyvanse (prodrug), Dexedrine

*Exercise Interactions:*

- **Cardiovascular**: ↑ Heart rate + BP (caution with intense cardio/weights)
- **Thermoregulation**: ↑ Heat stroke risk (avoid hot yoga/saunas)
- **Metabolic**: Accelerated med metabolism → shorter duration
- **Hydration**: Masked thirst → dehydration risk
- **Recovery**: May blunt fatigue signals → overexertion

*Pro Tips:*
• Monitor HR (keep <85% max)
• Pre-hydrate + electrolyte balance
• Time workouts before peak or during taper
• Avoid max lifts (BP spikes)

**2. Non-Stimulants (Second-Line)**
*Examples:*

- **Atomoxetine (Strattera)**: Delayed onset (weeks), steady effect
- **Alpha-2 Agonists**: Guanfacine ER (Intuniv), Clonidine ER (Kapvay)

*Exercise Interactions:*

- **Strattera**: Early fatigue → gradual intensity
- **Alpha-2s**: ↓ BP → watch for dizziness (yoga inversions)
- **Recovery**: No interference with muscle repair

**3. Off-Label Options**
*Examples:*

- Bupropion (Wellbutrin) - Seizure threshold caution
- Modafinil (Provigil) - Less CV impact

**Exercise Recommendations by Sport Type**

**Cardio Athletes:**

- Stimulants: Monitor HR zones
- Non-stims: Ideal for endurance

**Strength Training:**

- Avoid 1RM attempts on stimulants
- Non-stims allow heavier lifts

**Mind-Body (Yoga/Pilates):**

- Alpha-2 agonists → slow position changes
- Stimulants may help focus

**Competitive Sports:**

- Check WADA rules (many stimulants banned)
- Non-stims generally permitted

**Optimizing Your Routine**

1. **Med Timing**: Exercise during "troughs" (e.g., pre-dose or late-day)
2. **Hydration**: Weigh pre/post workout (replace 150% loss)
3. **Nutrition**: Protein-rich post-workout (stimulants suppress appetite)
4. **Recovery**: Prioritize sleep (meds may disrupt)

**Red Flags to Watch:**
Chest pain during exertion
Post-workout crashes
Excessive post-exercise fatigue

*Clinical Note:* Regular exercise actually boosts medication effectiveness long-term by upregulating dopamine receptors.

Would you like a sample workout plan tailored to your specific medication?

**Time 4**

- 1. **What is ADHD?**

**ADHD (Attention-Deficit/Hyperactivity Disorder) Redefined: A Neurodivergent Brain Wiring**

ADHD is not a behavior problem or lack of discipline - it's a neurodevelopmental condition characterized by **dysregulation of key brain networks** that control:

1. **Attention Allocation**
   - Not an attention *deficit*, but an attention *mismatch*
   - Can hyperfocus for hours on engaging tasks
   - Struggles to direct attention to mundane tasks
2. **Executive Functioning**
   - The brain's "CEO" is understaffed
   - Chronic challenges with:
     • Starting/completing tasks
     • Working memory (losing track mid-activity)
     • Organization/time management
     • Emotional regulation
3. **Reward Processing**
   - Dopamine-seeking behavior
   - Brain requires immediate rewards/consequences
   - "Time blindness" - poor future consequence awareness

**The ADHD Brain Difference:**
• Structurally different prefrontal cortex development
• Altered dopamine/norepinephrine signaling
• Reduced connectivity between brain networks

**Core Symptoms Manifest As:**

- **Hyperactivity** (physical or mental restlessness)
- **Impulsivity** (verbal, emotional, or behavioral)
- **Inattention** (distractibility or hyperfocus)

**Modern Understanding:**

- Present from childhood (though may be diagnosed later)
- Genetic in 70-80% of cases
- A lifespan condition (not just for kids)
- Often coexists with:
  • Learning disabilities
  • Anxiety/depression
  • Sleep disorders

**The Paradox:**
Can demonstrate **extraordinary focus** on passions while struggling with basic tasks - not a lack of capability, but a **mismatch between demands and brain wiring**.

Treatment focuses on **managing impairments while leveraging unique strengths** like creativity, resilience, and out-of-the-box thinking. Would you like me to elaborate on any specific aspect?

- 1. **What are its symptoms?**

**ADHD Symptoms: Beyond the Textbook List**

ADHD symptoms are best understood as **mismatches between brain wiring and environmental demands**. They manifest across three core domains, but with important nuances:

**1. Cognitive Symptoms (The "Thinking" Challenges)**

*Executive Dysfunction in Action:*

- **Task Initiation Paralysis**
  - Standing frozen knowing you need to start something but *can't*
  - "I'll do it in 5 minutes" turning into 5 hours
- **Working Memory Gaps**
  - Walking into rooms and forgetting why
  - Missing obvious steps in processes
- **Time Agnosia ("Time Blindness")**
  - Chronically late despite best efforts
  - Unable to estimate how long tasks take
- **Hyperfocus/Hypofocus**
  - Losing 6 hours on a hobby while ignoring hunger/bathroom
  - Or: Mind constantly hopping between unfinished tasks

**2. Emotional Symptoms (The "Feeling" Challenges)**

*The Overlooked Emotional Rollercoaster:*

- **Rejection Sensitive Dysphoria (RSD)**
  - Extreme emotional pain from perceived criticism
  - May lead to people-pleasing or avoidance
- **Emotional Tsunamis**
  - Quick bursts of intense anger/frustration that fade fast
  - Laughing/crying more intensely than others
- **Interest-Based Nervous System**
  - Can focus on *fascinating* tasks but not *important* ones
  - Motivation exists only for novel/urgent tasks

**3. Physical Symptoms (The "Body" Challenges)**

*How ADHD Lives in the Body:*

- **Restless Body Syndrome**
  - Constant fidgeting, leg bouncing, or skin picking
  - "I look calm but my toes are dancing in my shoes"
- **Sensory Seeking/Avoidance**
  - Craving loud music/intense exercise OR
  - Overwhelmed by scratchy clothes/bright lights
- **Movement Coordination Issues**
  - Clumsiness, frequent minor accidents
  - "I bruised myself on a doorframe... again"

**The ADHD Paradoxes**

What makes ADHD so misunderstood:
✔ Can hyperfocus for hours... but can't pay bills on time
✔ Remembers obscure facts... forgets appointments
✔ Excellent in crises... struggles with daily tasks
✔ Deeply creative... organizationally challenged

**Gender & Age Differences**

**Women/Girls** Often Show:

- Internal restlessness vs. physical hyperactivity
- Daydreaming, perfectionism, people-pleasing
- More likely to be diagnosed in adulthood

**Adults vs. Children:**

- Less obvious hyperactivity (turns to mental restlessness)
- More self-awareness but also more shame/self-criticism
- Life demands (bills, jobs) expose executive dysfunction

**Key Insight:** ADHD isn't about *can't* but *can't consistently*. The same person might:

- Write a brilliant paper in one night (hyperfocus)
- Then miss a deadline the next week (task paralysis)

Would you like me to focus on how these show up in specific contexts (work, school, relationships)? Or explore the neuroscience behind them?

- 1. **What medications are used for ADHD, and do they conflict with physical exercise?**

**ADHD Medications & Exercise: A Science-Backed Guide**

**1. Stimulant Medications (First-Line Treatment)**

**Examples:**

- **Methylphenidates**: Ritalin (short-acting), Concerta (long-acting), Focalin
- **Amphetamines**: Adderall (mixed salts), Vyvanse (lisdexamfetamine), Dexedrine

**How They Work:**
Boost dopamine/norepinephrine to improve focus and impulse control.

**Exercise Interactions:**
 **Potential Benefits:**

- May enhance workout focus/motivation
- Some athletes report better endurance (though debated)

**Key Risks:**

- **Cardiovascular strain**: ↑ Heart rate + blood pressure (avoid HIIT/max lifts)
- **Dehydration**: Masks thirst signals (drink water proactively)
- **Overheating**: Reduces sweat response (caution in hot yoga/saunas)
- **Crash potential**: Energy drop when meds wear off post-workout

**Pro Tips:**
• Monitor heart rate (keep <85% max)
• Pre-hydrate with electrolytes
• Time workouts for medication "troughs" (e.g., pre-dose or late-day)

**2. Non-Stimulant Medications (Second-Line)**

**Examples:**

- **Atomoxetine (Strattera)**: Norepinephrine modulator (takes weeks to work)
- **Alpha-2 Agonists**: Guanfacine ER (Intuniv), Clonidine ER (Kapvay)

**Exercise Interactions:**
 **Advantages:**

- No cardiovascular overstimulation
- Better for endurance athletes

**Considerations:**

- **Strattera**: May cause early workout fatigue (start slow)
- **Alpha-2s**: Can lower BP → dizziness (rise slowly after floor exercises)

**3. Off-Label/Other Options**

- **Bupropion (Wellbutrin)**: May lower seizure threshold in extreme endurance sports
- **Modafinil (Provigil)**: Less impact on HR/BP but limited ADHD data

**Exercise Recommendations by Sport**

| **Activity** | **Stimulant Caution** | **Non-Stimulant Tip** |
| --- | --- | --- |
| **Running/Cycling** | Monitor HR; avoid heat | Strattera: Pace early workouts |
| **Weightlifting** | Skip 1RM attempts (BP spikes) | Alpha-2s: Move slowly |
| **Yoga/Pilates** | Watch overheating | Rise slowly to avoid dizziness |
| **Team Sports** | Check WADA bans (many stimulants prohibited) | Generally safe |

**The Bottom Line**

- **Stimulants**: Require hydration/HR monitoring but can be used safely
- **Non-Stimulants**: Fewer restrictions but may need workout adjustments
- **All Medications**: Exercise *improves* ADHD symptoms long-term by boosting dopamine sensitivity

**When to Worry:**
 Chest pain/dizziness during exercise
 Severe post-workout crashes
 Resting HR >100 bpm on medication

**Pro Tip:** Pair workouts with protein snacks to counter appetite suppression from stimulants.

Would you like a personalized medication/exercise plan? Share your current regimen!

**Time 5**

1. **What is ADHD?**

**ADHD (Attention-Deficit/Hyperactivity Disorder) is a neurodevelopmental condition characterized by persistent patterns of inattention, hyperactivity, and impulsivity that stem from fundamental differences in brain structure and function.** Unlike temporary focus challenges, ADHD represents a hardwired variation in how the brain manages attention, motivation, and self-regulation.

**Core Characteristics:**

1. **Executive Dysfunction**
   - The brain struggles with:
     • Task initiation ("I know I need to start but can't")
     • Working memory (walking into rooms and forgetting why)
     • Emotional regulation (intense feelings that shift rapidly)
     • Cognitive flexibility (getting "stuck" on thoughts/behaviors)
2. **Interest-Based Nervous System**
   - Can laser-focus for hours on engaging tasks (hyperfocus)
   - Physically cannot force focus on mundane tasks (even when wanting to)
   - Requires immediate consequences/rewards for motivation
3. **Time Perception Differences**
   - "Time blindness" - cannot intuitively sense time passing
   - Chronic underestimation/overestimation of task durations
   - Now/not-now thinking (poor future consequence processing)

**The ADHD Brain:**

• **Prefrontal Cortex**: Develops ~3 years slower (affecting judgment/planning)
• **Neurotransmitters**: Dysregulated dopamine/norepinephrine systems
• **Connectivity**: Altered communication between brain networks

**Paradoxical Strengths:**
 Creativity and out-of-the-box thinking
 Crisis management (thrives under pressure)
 Observational skills (notices details others miss)
 High energy for passionate interests

**Diagnosis Requires:**

- Symptoms before age 12 (though often recognized later)
- Impairment in ≥2 settings (work, home, school)
- Ruling out other conditions (anxiety, thyroid issues, sleep disorders)

**Not Just "Bad Behavior":**
ADHD is a neurobiological difference, not a character flaw. Modern approaches focus on **accommodating brain differences** rather than forcing neurotypical standards.

Would you like me to explain how this manifests differently across ages/genders? Or explore the overlap with other neurodivergent conditions?

1. **What are its symptoms?**

Here’s a detailed yet concise breakdown of ADHD symptoms, organized to highlight their real-world impact and paradoxes:

**ADHD Symptoms: The Full Spectrum**

*(Beyond just "can't focus")*

**1. Attention Regulation (The Misnamed Symptom)**

- **Hyperfocus**:
  - Loses 4 hours researching a hobby while ignoring hunger/bathroom
  - "I can focus fine... but only on things that light up my brain"
- **Hypofocus**:
  - Mind hopping between 5 tasks, finishing none
  - Eyes glaze over during routine conversations

**2. Executive Dysfunction**

- **Task Paralysis**:
  - Standing frozen, screaming internally to start a simple task
  - Repeats "I’ll do it soon" for hours
- **Working Memory Gaps**:
  - Forgets why they entered a room mid-stride
  - Leaves stove on, keys in fridge, or loses phones daily
- **Time Blindness**:
  - Chronically late despite multiple alarms
  - Can’t estimate if a task takes 5 minutes or 2 hours

**3. Hyperactivity (Not Just for Kids)**

- **Physical**:
  - Leg bouncing, nail-picking, or constant fidgeting
  - "I look calm but my toes are dancing in my shoes"
- **Mental**:
  - 10 browser tabs + 3 unfinished thoughts + sudden urge to reorganize desk
  - Brain "noise" that won’t quiet

**4. Emotional Dysregulation**

- **Rejection Sensitivity**:
  - Obsessing over a minor comment for days
  - Preemptive people-pleasing to avoid criticism
- **Frustration Tsunamis**:
  - Sudden rage over tiny inconveniences (then quick shame)
  - Tears when overwhelmed by sensory input (loud noises/clutter)

**5. Motivation Paradoxes**

- **Interest-Based Performance**:
  - Writes a masterpiece paper at 3 AM (deadline panic)
  - Can’t reply to a simple email for weeks
- **Reward System Glitches**:
  - Needs immediate consequences to act
  - "I know it’s important... but my brain says it’s not *urgent*"

**Gender & Age Differences**

**Women/Girls Often Show:**

- Internal restlessness vs. physical hyperactivity
- Daydreaming + perfectionism masking struggles
- Diagnosed later (if ever)

**Adults vs. Children:**

- Less "running around" → more mental restlessness
- Life demands expose executive dysfunction (bills, jobs)
- Shame from years of being called "lazy" or "spacey"

**The ADHD Iceberg**

**What’s Visible:**

- Forgetfulness
- Messy space
- Running late

**What’s Hidden Underwater:**

- Exhaustion from trying to appear "normal"
- Anxiety about unmet responsibilities
- Grief over wasted potential

**Key Insight:** ADHD isn’t a lack of focus—it’s **inconsistent focus control**. The same person might hyperfixate for hours then struggle to brush their teeth.

Would you like examples of how these show up in work/relationships? Or coping strategies that actually work with (not against) ADHD brains?

1. **What medications are used for ADHD, and do they conflict with physical exercise?**

**ADHD Medications & Exercise: A Science-Based Guide**

**1. Stimulants (First-Line Treatment)**

**Common Types:**

- **Methylphenidates**: Ritalin (short-acting), Concerta (long-acting), Focalin
- **Amphetamines**: Adderall (mixed salts), Vyvanse (prodrug), Dexedrine

**Exercise Effects:**

- **Benefits**:
  - May improve workout focus and endurance (for some)
  - Helps overcome task initiation barriers to exercise
- **Risks**:

| **Risk** | **Solution** |
| --- | --- |
| Elevated heart rate/blood pressure | Monitor HR; avoid max-effort training |
| Dehydration (reduced thirst cues) | Pre-hydrate with electrolytes |
| Overheating (impaired thermoregulation) | Skip hot yoga/saunas; cool down properly |
| Energy crashes post-workout | Time exercise before meds peak or during taper |

**Pro Tip:** Use a fitness tracker to keep heart rate <85% of max during cardio.

**2. Non-Stimulants (Second-Line Options)**

**Common Types:**

- **Atomoxetine (Strattera)**: Norepinephrine modulator
- **Alpha-2 Agonists**: Guanfacine ER, Clonidine ER

**Exercise Effects:**

- **Benefits**:
  - No cardiovascular overstimulation
  - Better for endurance athletes
- **Considerations**:

| **Medication** | **Exercise Impact** |
| --- | --- |
| Strattera | May cause early workout fatigue (build intensity gradually) |
| Guanfacine/Clonidine | Can cause dizziness (move slowly between positions) |

**3. Off-Label Options**

- **Bupropion**: Caution with extreme endurance sports (seizure risk)
- **Modafinil**: Minimal cardio impact but less effective for core ADHD symptoms

**Exercise Recommendations by Sport**

| **Activity** | **Stimulant Users** | **Non-Stimulant Users** |
| --- | --- | --- |
| **Running/Cycling** | Monitor HR; avoid heat | Ideal for endurance training |
| **Weightlifting** | Skip 1RM attempts | Safer for heavy lifts |
| **Yoga** | Watch overheating | Move slowly in inversions |
| **Team Sports** | Check WADA bans | Generally unrestricted |

**Key Takeaways**

1. **Stimulants** require careful hydration/HR monitoring but can be used safely.
2. **Non-stimulants** are more exercise-friendly but may need gradual intensity buildup.
3. **All ADHD meds** work better when combined with regular exercise (boosts dopamine sensitivity long-term).

**Red Flags to Watch:**
 Chest pain/dizziness during exercise
 Resting HR >100 bpm on medication
 Severe post-workout crashes

**Pro Tip:** Pair workouts with protein snacks to counter medication-induced appetite suppression. For competitive athletes, Vyvanse or non-stimulants are often preferred (fewer WADA restrictions).

Would you like help designing a medication-friendly workout schedule? Share your current routine!

**Time 6**

- 1. **What is ADHD?**

**ADHD (Attention-Deficit/Hyperactivity Disorder) is a neurodevelopmental condition characterized by persistent differences in brain structure and function that affect self-regulation, attention, and behavior.** It's not a behavior problem or lack of discipline—it's a hardwired variation in how the brain manages focus, motivation, and impulse control.

**Core Features of ADHD**

1. **Executive Dysfunction**
   - The brain's "management system" struggles with:
     • Starting/completing tasks (even when wanting to)
     • Working memory (forgetting what you're doing mid-task)
     • Organization/time management ("time blindness")
     • Emotional regulation (intense, quick-changing feelings)
2. **Interest-Based Attention**
   - Can hyperfocus for hours on engaging tasks
   - Physically cannot force focus on mundane tasks
   - Requires immediate rewards/consequences for motivation
3. **Neurological Basis**
   - Slower prefrontal cortex development (affects planning/judgment)
   - Dopamine/norepinephrine system differences
   - Often runs in families (74% heritability)

**ADHD Symptoms Beyond Stereotypes**

**Not Just "Can't Sit Still":**

- **Inattention**: Losing track of conversations, chronic forgetfulness
- **Hyperactivity**: Mental restlessness (adults) or physical fidgeting
- **Impulsivity**: Interrupting, impulsive decisions (spending, relationships)

**Hidden Challenges:**

- Rejection sensitivity (extreme emotional pain from criticism)
- Sensory issues (overwhelmed by sounds/textures)
- Sleep problems (delayed circadian rhythm)

**ADHD Strengths Often Overlooked**

✔ Creativity and out-of-the-box thinking
✔ Crisis management (thrives under pressure)
✔ Observational skills (notices what others miss)
✔ High energy for passionate interests

**Diagnosis & Treatment**

**Requires:**

- Symptoms before age 12 (even if unrecognized)
- Impairment in multiple life areas
- Ruling out other conditions (anxiety, thyroid issues)

**Management Includes:**

- Medication (stimulants/non-stimulants)
- Behavioral therapy (CBT, coaching)
- Accommodations (work/school supports)
- Lifestyle adaptations (exercise, sleep hygiene)

**Key Insight:** ADHD isn't an attention *deficit*, but an attention *mismatch*—the brain struggles to control what it pays attention to, not with attention itself. With proper support, ADHD brains can thrive by working *with* their neurology rather than against it.

Would you like more on specific aspects (e.g., gender differences, adult diagnosis)?

- 1. **What are its symptoms?**

**ADHD Symptoms: A Modern, Nuanced Breakdown**

ADHD manifests as **persistent patterns of dysregulation** in attention, behavior, and emotions. The symptoms exist on a spectrum and often contradict each other, creating what specialists call the **"ADHD paradoxes."**

**Core Symptom Clusters**

**1. Executive Dysfunction**

*(The brain's "CEO" is understaffed)*

- **Task Initiation Paralysis**
  - Standing frozen, mentally screaming to start a simple task
  - "I'll do it in 5 minutes" → 5 hours pass
- **Working Memory Gaps**
  - Walking into rooms and forgetting why
  - Missing obvious steps in processes (e.g., pouring coffee before mug)
- **Time Blindness**
  - Chronically late despite 10 alarms
  - Can't estimate if a task takes 5 minutes or 2 hours

**2. Attention Dysregulation**

*(Not a deficit, but inconsistent control)*

- **Hyperfocus**
  - Losing 6 hours on a hobby while ignoring hunger/bathroom
  - "Wait, it's dark outside now?"
- **Hypofocus**
  - Mind hopping between 3 conversations + 2 thoughts + 1 song lyric
  - Eyes glazing over during important meetings

**3. Impulse/Emotion Challenges**

*(The emotional rollercoaster)*

- **Verbal Impulsivity**
  - Interrupting others mid-sentence (then feeling guilty)
  - Oversharing personal details
- **Emotional Tsunamis**
  - Sudden rage over tiny inconveniences (that fades quickly)
  - Crying from sensory overload (e.g., loud chewing sounds)
- **Rejection Sensitivity**
  - Obsessing for days over a minor comment
  - People-pleasing to avoid perceived criticism

**4. Physical Manifestations**

*(How ADHD lives in the body)*

- **Restless Body Syndrome**
  - Leg bouncing, skin picking, or constant fidgeting
  - "I look calm but my toes are dancing in my shoes"
- **Sensory Issues**
  - Hating itchy tags or loud noises **OR**
  - Craving intense stimulation (loud music/spicy food)

**The ADHD Paradoxes**

*(Why it's misunderstood)*
✔ Can hyperfocus for hours... but can't pay bills on time
✔ Remembers obscure facts... forgets anniversaries
✔ Excellent in crises... overwhelmed by daily tasks
✔ Deeply creative... chronically disorganized

**Gender & Age Differences**

**Women/Girls Often Show:**

- Internal restlessness vs. hyperactivity
- Perfectionism masking struggles
- More likely diagnosed in adulthood

**Adults vs. Children:**

- Less "running around" → more mental restlessness
- Life demands expose executive dysfunction (bills, parenting)
- Years of shame ("Why can't I just...?")

**Real-World Impact**

**At Work/School:**

- Missed deadlines despite all-nighters
- Desk looks like a tornado hit it
- Brilliant ideas... half-finished projects

**In Relationships:**

- Forgetting important dates (but remembering random details)
- Zoning out during conversations
- Emotional outbursts followed by deep remorse

**Key Insight:** ADHD isn't about ability—it's about **inconsistent performance**. The same person might:

- Write a masterpiece paper in one night
- Then struggle to brush their teeth the next morning

Would you like:

1. **Coping strategies** tailored to these symptoms?
2. How to **explain this to neurotypical people**?
3. The **science behind why these symptoms occur**?
   1. **What medications are used for ADHD, and do they conflict with physical exercise？**

**ADHD Medications & Exercise: A Practical Guide**

**1. Stimulant Medications (Most Common)**

**Examples:**

- **Methylphenidates**: Ritalin (short-acting), Concerta (long-acting)
- **Amphetamines**: Adderall (mixed salts), Vyvanse (prodrug), Dexedrine

**How They Work:**
Boost dopamine/norepinephrine to improve focus and impulse control.

**Exercise Interactions:**

| **Effect** | **Management Tips** |
| --- | --- |
| ↑ Heart rate & BP | Avoid HIIT/max lifts; monitor with fitness tracker |
| ↓ Thirst perception | Pre-hydrate with electrolytes |
| ↑ Heat stroke risk | Skip hot yoga/saunas; cool down properly |
| Energy crashes post-workout | Time exercise before meds peak or during taper |

**Pro Tip:** Moderate cardio (jogging, swimming) is generally safer than extreme endurance sports.

**2. Non-Stimulant Medications**

**Examples:**

- **Atomoxetine (Strattera)**: Norepinephrine modulator (takes 4-8 weeks to work)
- **Alpha-2 Agonists**: Guanfacine ER (Intuniv), Clonidine ER

**Exercise Interactions:**

| **Medication** | **Effect** | **Solution** |
| --- | --- | --- |
| Strattera | Early workout fatigue | Start with low-intensity walks |
| Guanfacine | Dizziness (↓ BP) | Rise slowly from floor exercises |

**Best For:** Yoga, Pilates, or strength training (less cardiovascular impact).

**Exercise Recommendations by Sport**

| **Activity** | **Stimulant Users** | **Non-Stimulant Users** |
| --- | --- | --- |
| **Running** | Monitor HR; avoid heat | Ideal for endurance |
| **Weightlifting** | Skip 1RM attempts | Safer for heavy lifts |
| **Team Sports** | Check WADA bans* | Generally unrestricted |

*Many stimulants are banned in competitive sports. Vyvanse has slightly fewer restrictions.

**Key Takeaways**

1. **Stimulants** require hydration/HR monitoring but can enhance focus for workouts.
2. **Non-stimulants** are exercise-friendly but may need gradual intensity adjustments.
3. **All ADHD meds** work better with regular exercise (long-term dopamine regulation).

**Red Flags:**
 Chest pain/dizziness during exercise
 Resting HR >100 bpm on medication
 Severe post-workout crashes

**Pro Tip:** Pair workouts with protein snacks to counter medication-induced appetite suppression.

**Need a personalized plan?** Share your:

- Current medication
- Favorite exercise types
- Any side effects you’ve noticed

**Times 7**

1. **What is ADHD?**

**ADHD (Attention-Deficit/Hyperactivity Disorder) is a neurodevelopmental condition characterized by persistent differences in brain structure and function that impact self-regulation, motivation, and attention control.** It's not a behavior problem or character flaw—it's a hardwired variation in how the brain manages focus, impulses, and emotional responses.

**ADHD Redefined: Beyond the Stereotypes**

1. **Executive Dysfunction**
   - The brain struggles with:
     • **Task initiation** ("I want to start but my brain won't cooperate")
     • **Working memory** (walking into rooms and forgetting why)
     • **Time perception** ("5 minutes" vs. 2 hours feel identical)
     • **Emotional regulation** (intense feelings that shift rapidly)
2. **Interest-Based Nervous System**
   - Can hyperfocus for hours on passions (but not "boring" tasks)
   - Requires immediate consequences/rewards to motivate action
   - Thrives under pressure (last-minute adrenaline focus)
3. **Neurological Basis**
   - Prefrontal cortex develops ~3 years slower (affects planning/judgment)
   - Dopamine/norepinephrine systems work differently
   - Highly genetic (74-80% heritability)

**The ADHD Paradoxes**

✔ Can remember obscure facts but forget appointments
✔ Writes a brilliant paper at 3 AM but can't file taxes
✔ Exceptionally creative yet chronically disorganized
✔ Deeply empathetic but interrupts conversations

**Real-World Manifestations**

**At Work/School:**

- Missed deadlines despite all-nighters
- Desk chaos with "organized piles"
- Brilliant ideas abandoned halfway

**In Relationships:**

- Zoning out mid-conversation
- Emotional outbursts followed by guilt
- Forgetting anniversaries (but recalling random details)

**In the Body:**

- Constant fidgeting or restlessness
- Sensory sensitivities (hates tags/loud noises)
- Sleep problems (delayed circadian rhythm)

**ADHD Strengths**

- **Crisis management** (thrives under pressure)
- **Creative problem-solving** (connects unusual dots)
- **Hyperfocus productivity** (when engaged)
- **Resilience** (years of adapting to challenges)

**Diagnosis & Management**

**Requires:**

- Symptoms before age 12 (even if unrecognized)
- Impairment in ≥2 life areas (work, relationships, self-care)
- Ruling out similar conditions (anxiety, thyroid issues)

**Treatment Works Best When:**

- Combines medication + behavioral strategies
- Accommodates brain differences (not forces "normalcy")
- Leverages strengths (creativity, crisis skills)

**Key Insight:** ADHD isn't an attention *deficit*—it's an attention *mismatch*. The brain struggles to control *what* it pays attention to, not with attention itself. With proper support, ADHD brains can thrive by working *with* their neurology rather than against it.

Would you like more on:

1. **Gender differences** in symptoms?
2. **Adult diagnosis** clues?
3. **Coping strategies** that actually work?
4. **What are its symptoms?**

Here’s a comprehensive yet concise breakdown of ADHD symptoms, organized to reflect real-world experiences:

**Core ADHD Symptoms (Beyond the Textbook List)**

**1. Executive Dysfunction**

- **Task Paralysis**: Physically unable to start tasks (even urgent ones)
- **Working Memory Gaps**: Forgetting what you're saying mid-sentence
- **Time Blindness**: Chronic lateness despite multiple alarms
- **Hyperfocus**: 6-hour deep dives into hobbies while ignoring basic needs

**2. Attention Regulation**

- **Hypofocus**: Mind hopping between 3 thoughts + 2 conversations + 1 song lyric
- **Selective Attention**: Missing obvious details while noticing irrelevant ones
- **Sensory Distractibility**: Unable to focus with background noise/clutter

**3. Impulse/Emotion Challenges**

- **Verbal Impulsivity**: Interrupting others despite trying not to
- **Emotional Tsunamis**: Intense frustration over minor inconveniences
- **Rejection Sensitivity**: Physical pain from perceived criticism

**4. Physical Manifestations**

- **Restless Body Syndrome**: Constant fidgeting, leg bouncing, skin picking
- **Movement Coordination**: Frequent minor accidents ("I bruise on doorframes")
- **Sleep Issues**: Night owl tendencies, difficulty waking

**The ADHD Paradoxes**

✔ Can hyperfocus for hours but can't pay bills
✔ Remembers obscure facts but forgets appointments
✔ Excellent in crises but overwhelmed by daily life
✔ Deeply creative but organizationally challenged

**Gender & Age Differences**

**Women/Girls Often Experience:**

- Internal restlessness vs. hyperactivity
- Perfectionism masking struggles
- More likely diagnosed in adulthood

**Adults vs. Children:**

- Less physical hyperactivity → more mental restlessness
- Life responsibilities expose executive dysfunction
- Years of accumulated shame ("Why can't I adult?")

**Real-World Impact**

**At Work:**

- Missed deadlines despite all-nighters
- Bursts of brilliance between periods of stagnation
- Desk looks like organized chaos

**In Relationships:**

- Zoning out during important conversations
- Emotional outbursts followed by remorse
- Forgetting plans but remembering random details

**Key Insight:** ADHD symptoms represent a *mismatch* between brain wiring and environmental demands, not a lack of capability. The same person might:

- Solve complex problems at work
- Struggle to do laundry for weeks
- Create amazing art
- Forget to eat for 12 hours

Would you like:

1. **Coping strategies** for specific symptoms?
2. How to **explain these experiences** to neurotypical people?
3. The **science behind why these symptoms occur**?
4. **What medications are used for ADHD, and do they conflict with physical exercise?**

Here's a clear, actionable guide to ADHD medications and their interaction with exercise:

**ADHD Medication Types & Exercise Considerations**

**1. Stimulant Medications (First-Line)**

**Common Options:**

- **Methylphenidate**: Ritalin (3-4 hrs), Concerta (8-12 hrs), Focalin
- **Amphetamines**: Adderall (4-6 hrs), Vyvanse (10-14 hrs), Dexedrine

**Exercise Effects:**

| **Effect** | **Solution** | **Best For** |
| --- | --- | --- |
| ↑ Heart rate (5-10 bpm) | Monitor with fitness tracker | Moderate cardio |
| ↑ Blood pressure | Avoid heavy lifting/HIIT | Swimming, cycling |
| ↓ Thirst perception | Set hydration reminders | Indoor workouts |
| ↑ Heat sensitivity | Exercise in AC, avoid midday sun | Morning/evening workouts |
| Energy crashes | Time workouts before afternoon slump | Short, intense sessions |

**Pro Tip:** Vyvanse is often preferred for athletes due to smoother energy curve and fewer WADA restrictions.

**2. Non-Stimulant Options**

**Common Choices:**

- **Atomoxetine (Strattera)**: Takes 4-8 weeks to work
- **Guanfacine (Intuniv)**: Originally for high blood pressure

**Exercise Advantages:**

- No cardiovascular overstimulation
- Better for endurance athletes
- No WADA restrictions

**Watch For:**

- Early fatigue (Strattera) → Start with 20-min walks
- Dizziness (Guanfacine) → Rise slowly from floor exercises

**3. Off-Label Options**

- **Bupropion**: Avoid extreme endurance sports (seizure risk)
- **Modafinil**: Minimal exercise impact but weaker ADHD effects

**Exercise Recommendations by Medication Type**

| **Activity** | **Stimulant Users** | **Non-Stimulant Users** |
| --- | --- | --- |
| **Running** | Keep HR <85% max | Ideal for marathon training |
| **Weightlifting** | Avoid 1RM attempts | Safer for powerlifting |
| **Yoga** | Skip hot yoga | Great for stress relief |
| **Team Sports** | Check league bans | Generally unrestricted |

**Key Action Steps**

1. **For Stimulant Users:**
   - Work out before meds peak or as they taper
   - Hydrate with electrolyte drinks
   - Wear cooling gear in heat
2. **For Non-Stimulant Users:**
   - Gradually increase workout intensity
   - Add balance exercises (yoga/tai chi)
   - Use interval timers to maintain focus

**Red Flags to Stop Exercise:**
 Chest pain or irregular heartbeat
 Dizziness that doesn't resolve
 Sudden extreme fatigue

**Pro Tip:** Exercise actually enhances medication effectiveness long-term by improving dopamine sensitivity. Even 20 minutes of walking can boost focus.

Would you like a sample workout plan tailored to your specific medication?

**Times 8**

- 1. **What is ADHD?**

**ADHD (Attention-Deficit/Hyperactivity Disorder) is a neurodevelopmental condition characterized by persistent differences in brain structure and function that affect self-regulation.** It's not a behavioral problem or lack of discipline—it's a biologically-based variation in how the brain manages attention, motivation, and impulse control.

**Core Characteristics:**

1. **Executive Dysfunction**
   - The brain's "management system" struggles with:
     • Task initiation (the "wall of awful" when starting tasks)
     • Working memory (forgetting what you're doing mid-activity)
     • Emotional regulation (intense feelings that shift rapidly)
     • Cognitive flexibility (getting mentally "stuck")
2. **Interest-Based Nervous System**
   - Can hyperfocus for hours on passions (but not mundane tasks)
   - Requires immediate consequences/rewards to take action
   - "Time blindness" (5 minutes vs. 2 hours feel identical)
3. **Neurological Basis**
   - Prefrontal cortex develops ~3 years slower
   - Dopamine/norepinephrine systems work differently
   - Highly genetic (about 75-80% heritability)

**The ADHD Paradoxes:**

✔ Can remember obscure facts but forget appointments
✔ Writes brilliant work under deadline but can't file taxes
✔ Exceptionally creative yet chronically disorganized
✔ Deeply empathetic but interrupts conversations

**Real-World Impact:**

**At Work/School:**

- Bursts of hyperproductivity between periods of stagnation
- "Organized chaos" systems that confuse others
- Missed deadlines despite good intentions

**In Relationships:**

- Zoning out during conversations (not due to lack of care)
- Emotional outbursts followed by quick remorse
- Forgetting important dates despite genuine effort

**In the Body:**

- Constant fidgeting or restlessness
- Sensory sensitivities (hates tags/loud noises)
- Sleep problems (delayed circadian rhythm)

**ADHD Strengths Often Overlooked:**

- **Crisis management** (thrives under pressure)
- **Creative problem-solving** (makes unusual connections)
- **Resilience** (years of adapting to challenges)
- **Observational skills** (notices what others miss)

**Diagnosis & Management:**

**Requires:**

- Symptoms present since childhood (even if unrecognized)
- Impairment in multiple life areas
- Ruling out other conditions (anxiety, thyroid issues)

**Effective Approaches:**

- Medication (stimulants/non-stimulants)
- Behavioral strategies (externalizing memory/time)
- Accommodations (structured environments)
- Leveraging strengths (creative roles, flexible schedules)

**Key Insight:** ADHD isn't an attention deficit—it's an **attention inconsistency**. The brain doesn't lack focus, but struggles to *control* what it focuses on. With proper understanding and support, ADHD can become a superpower rather than a limitation.

Would you like more on:

1. **Gender differences** in symptoms?
2. **Adult diagnosis** clues?
3. **Workplace accommodations** that help?
   1. **What are its symptoms?**

**ADHD Symptoms: The Complete Modern Guide**

ADHD symptoms aren't just "trouble focusing" – they're a constellation of **brain-based differences** that affect attention, behavior, and emotions in paradoxical ways. Here's the full picture:

**1. Executive Dysfunction (The Hidden Struggle)**

*Your brain's "CEO" is understaffed:*

- **Task Initiation Paralysis**
  - Standing frozen, screaming internally to start simple tasks
  - "I'll do it in 5 minutes" → 5 hours vanish
- **Working Memory Gaps**
  - Walking into rooms and forgetting why
  - Putting milk in the pantry, keys in fridge
- **Time Blindness**
  - Chronically late despite 10 alarms
  - Can't estimate if something takes 5 minutes or 2 hours

**2. Attention Dysregulation**

*Not too little attention – just uncontrollable attention:*

- **Hyperfocus**
  - Losing 6 hours on a hobby while ignoring hunger/bathroom
  - "Wait, when did it get dark outside?"
- **Hypofocus**
  - Mind bouncing between 3 thoughts + 2 conversations + 1 song lyric
  - Eyes glazing over during important meetings

**3. Emotional Rollercoaster**

*Feelings dialed up to 11:*

- **Rejection Sensitivity**
  - Physical pain from minor criticism
  - Obsessing over comments for days
- **Emotional Tsunamis**
  - Sudden rage over tiny frustrations (that fades fast)
  - Crying from sensory overload (loud chewing sounds)

**4. Physical Manifestations**

*How ADHD lives in your body:*

- **Restless Body Syndrome**
  - Constant leg bouncing, skin picking, or hair twirling
  - "I look calm but my toes are dancing in my shoes"
- **Sensory Issues**
  - Hating scratchy fabrics/loud noises **OR**
  - Craving intense stimulation (spicy food/loud music)

**The ADHD Paradoxes**

*Why it's so misunderstood:*
✔ Can hyperfocus for hours... but can't pay bills
✔ Remembers obscure facts... forgets anniversaries
✔ Excellent in crises... overwhelmed by daily tasks
✔ Deeply creative... chronically disorganized

**Gender & Age Differences**

**Women/Girls Often Experience:**

- Internal restlessness vs. hyperactivity
- Perfectionism masking struggles
- More likely diagnosed in adulthood

**Adults vs. Children:**

- Less "running around" → more mental restlessness
- Life responsibilities expose executive dysfunction
- Years of accumulated shame ("Why can't I just...?")

**Real-World Impact**

**At Work/School:**

- Missed deadlines despite all-nighters
- Bursts of brilliance between periods of stagnation
- Desk looks like organized chaos

**In Relationships:**

- Zoning out mid-conversation (despite caring)
- Emotional outbursts followed by deep remorse
- Forgetting plans but remembering random details

**Key Insight:** ADHD isn't about ability – it's about **inconsistent access** to abilities. The same person might:

- Solve complex problems at work
- Struggle to do laundry for weeks
- Create amazing art
- Forget to eat for 12 hours

**Need Help With:**

1. **Coping strategies** for specific symptoms?
2. How to **explain this to others**?
3. **Professional assessment** guidance?

Let me know what matters most to you!

- 1. **What medications are used for ADHD, and do they conflict with physical exercise?**

**ADHD Medications & Exercise: A Science-Backed Guide**

**1. Stimulant Medications (First-Line Treatment)**

**How They Work:** Boost dopamine/norepinephrine to improve focus and impulse control.

**Common Options:**

- **Methylphenidates**: Ritalin (short-acting), Concerta (long-acting)
- **Amphetamines**: Adderall (mixed salts), Vyvanse (prodrug)

**Exercise Interactions:**

| **Effect** | **Solution** | **Best Exercises** |
| --- | --- | --- |
| ↑ Heart rate (5-15 bpm) | Monitor with fitness tracker | Swimming, cycling |
| ↑ Blood pressure | Avoid heavy lifting/HIIT | Moderate weight training |
| ↓ Thirst perception | Set hydration alarms | Indoor workouts |
| ↑ Heat intolerance | Exercise in cool hours | Morning/evening sessions |
| Energy crashes | Time workouts pre-dose or during taper | Short, intense workouts |

**Pro Tip:** Vyvanse is often preferred by athletes for its smoother effect and fewer WADA restrictions.

**2. Non-Stimulant Options**

**Best For:** Those sensitive to stimulants or with heart concerns.

**Common Choices:**

- **Atomoxetine (Strattera)**: Takes 4-8 weeks to work
- **Guanfacine (Intuniv)**: Originally for high blood pressure

**Exercise Advantages:**

- No cardiac overstimulation
- Better for endurance sports
- No WADA bans

**Watch For:**

- Early fatigue (Strattera) → Start with 20-min walks
- Dizziness (Guanfacine) → Rise slowly from floor moves

**3. Off-Label Options**

- **Bupropion**: Caution with extreme endurance sports
- **Modafinil**: Minimal exercise impact but weaker ADHD effects

**Exercise Recommendations by Sport**

| **Activity** | **Stimulant Users** | **Non-Stimulant Users** |
| --- | --- | --- |
| **Running** | Keep HR <85% max | Ideal for distance |
| **Weightlifting** | Skip 1-rep max attempts | Safer for heavy lifts |
| **Yoga** | Avoid hot yoga | Great for stress relief |
| **Team Sports** | Check league bans | Generally unrestricted |

**Key Action Steps**

1. **For Stimulants:**
   - Hydrate with electrolytes (liquid IV works well)
   - Wear cooling gear in heat
   - Consider shorter, more frequent workouts
2. **For Non-Stimulants:**
   - Gradually increase intensity over weeks
   - Add balance exercises (yoga/tai chi helps)
   - Use interval timers to maintain focus

**Red Flags to Stop:**
Chest pain or irregular heartbeat
 Dizziness that doesn't resolve with rest
 Sudden extreme fatigue or confusion

**Pro Tip:** Exercise actually enhances medication effects long-term by improving dopamine sensitivity. Even 10 minutes of jumping jacks can boost focus.

**Want a Personalized Plan?** Share:

1. Your current medication/dose
2. Favorite types of exercise
3. Any side effects you've noticed

I'll help optimize your routine!

**Times 9**

- 1. **What is ADHD?**

**ADHD (Attention-Deficit/Hyperactivity Disorder) is a neurodevelopmental condition characterized by persistent differences in brain structure and function that impact self-regulation, motivation, and cognitive control.** It's not a behavioral choice or character flaw, but a biological variation in how the brain processes information and manages attention.

**Core Features:**

1. **Executive Dysfunction**
   - The brain's "management system" struggles with:
     • Task initiation (the "wall of awful" when starting tasks)
     • Working memory (forgetting steps mid-process)
     • Emotional regulation (intense, quick-changing feelings)
     • Cognitive flexibility (getting mentally "stuck")
2. **Interest-Based Nervous System**
   - Can hyperfocus for hours on passions
   - Struggles with "boring" but important tasks
   - Requires immediate consequences to take action
   - Experiences "time blindness" (poor time perception)
3. **Neurological Basis**
   - Prefrontal cortex develops slower (~3 year delay)
   - Altered dopamine/norepinephrine systems
   - 74-80% heritability (strong genetic component)

**The ADHD Paradoxes:**

✔ Can remember obscure facts but forget appointments
✔ Produces brilliant work under pressure but can't do taxes
✔ Exceptionally creative yet chronically disorganized
✔ Deeply empathetic but interrupts conversations

**Real-World Impact:**

**At Work/School:**

- Cyclical productivity (bursts of hyperfocus vs. stagnation)
- "Organized chaos" systems that confuse others
- Missed deadlines despite good intentions

**In Relationships:**

- Zoning out during conversations (not from lack of care)
- Emotional outbursts followed by quick remorse
- Forgetting important dates despite genuine effort

**Physical Manifestations:**

- Constant fidgeting or restlessness
- Sensory sensitivities (hates tags/loud noises)
- Sleep problems (delayed circadian rhythm)

**ADHD Strengths:**

- **Crisis management** (thrives under pressure)
- **Creative problem-solving** (makes unique connections)
- **Resilience** (years of adapting to challenges)
- **Observational skills** (notices what others miss)

**Diagnosis & Management:**

**Requires:**

- Childhood-onset symptoms (even if unrecognized)
- Impairment in multiple life areas
- Ruling out similar conditions (anxiety, thyroid issues)

**Effective Approaches:**

- **Medication** (stimulants/non-stimulants)
- **Behavioral strategies** (externalizing memory/time)
- **Accommodations** (structured environments)
- **Strength-based** approaches (creative roles, flexible schedules)

**Key Insight:** ADHD isn't an attention deficit—it's **inconsistent attention control**. The challenge isn't lacking focus, but directing focus intentionally. With proper support, ADHD can become a superpower rather than a limitation.

**Need more on:**

1. How symptoms differ between genders?
2. Signs in high-achieving adults?
3. Best workplace accommodations?
   1. **What are its symptoms?**

Here’s a comprehensive yet streamlined breakdown of ADHD symptoms, organized to reflect real-world experiences:

**ADHD Symptoms: Beyond the Stereotypes**

**1. Executive Dysfunction (The "Brain Manager" Struggles)**

- **Task Paralysis**
  *"I need to start but my brain won't cooperate"*
  - Staring at unpaid bills for hours despite urgency
  - Endless scrolling instead of beginning work
- **Working Memory Gaps**
  *"What was I just doing?"*
  - Forgetting mid-sentence what you wanted to say
  - Leaving stove on, keys in fridge, or losing phones daily
- **Time Blindness**
  *"I thought that would take 5 minutes!"*
  - Chronically late despite multiple alarms
  - Underestimating how long tasks truly take

**2. Attention Dysregulation**

- **Hyperfocus**
  *"Wait, it's 3 AM?"*
  - Losing 6 hours researching a hobby while ignoring hunger
  - Workaholic tendencies when engaged
- **Hypofocus**
  *"Squirrel!" brain*
  - Mind hopping between 3 thoughts + 2 conversations
  - Eyes glazing over during meetings despite trying

**3. Emotional Intensity**

- **Rejection Sensitivity**
  *Physical pain from criticism*
  - Obsessing for days over minor feedback
  - People-pleasing to avoid disapproval
- **Emotional Tsunamis**
  *Zero to 100 in seconds*
  - Sudden rage over misplaced items (that quickly fades)
  - Tears from sensory overload (loud chewing, itchy clothes)

**4. Physical Manifestations**

- **Restless Body**
  *Constant motion needs*
  - Leg bouncing, skin picking, hair twirling
  - "I look calm but my toes are dancing"
- **Sensory Issues**
  *Hypersensitive or sensation-seeking*
  - Hating tags/loud noises OR craving spicy food/loud music

**The ADHD Paradoxes**

✔ Can hyperfocus for hours but can't do laundry
✔ Remembers obscure facts but forgets birthdays
✔ Excellent in crises but overwhelmed by chores
✔ Deeply creative but organizationally challenged

**Gender & Age Differences**

**Women/Girls Often Show:**

- Internal restlessness vs. hyperactivity
- Perfectionism masking struggles
- More likely diagnosed in adulthood

**Adults vs. Children:**

- Less physical hyperactivity → more mental restlessness
- Life responsibilities expose executive dysfunction
- Years of accumulated shame ("Why can't I adult?")

**Real-World Impact**

**At Work:**

- Bursts of brilliance between procrastination
- Desk covered in "organized chaos" piles
- Missing deadlines despite all-nighters

**In Relationships:**

- Zoning out mid-conversation (not from lack of care)
- Emotional outbursts followed by guilt
- Forgetting plans but remembering random details

**Key Insight:** ADHD isn't a lack of ability—it's **inconsistent access** to abilities. The same person might:

- Solve complex work problems
- Struggle to shower regularly
- Create amazing art
- Forget to eat all day

**Need help with:**

1. **Coping strategies** for specific symptoms?
2. How to **explain this to neurotypical people**?
3. **Professional assessment** guidance?

Let me know what would help you most!

- 1. **What medications are used for ADHD, and do they conflict with physical exercise?**

Here's a clinically-informed yet practical guide to ADHD medications and exercise interactions:

**ADHD Medication Types & Exercise Considerations**

**1. Stimulants (First-Line Treatment)**

**Common Options:**

- **Methylphenidates**: Ritalin (3-4 hrs), Concerta (8-12 hrs), Focalin XR
- **Amphetamines**: Adderall (4-6 hrs), Vyvanse (10-14 hrs), Dexedrine

**Exercise Science:**

- ↑ Heart rate (average +8-12 bpm)
- ↑ Systolic BP (10-15 mmHg)
- ↓ Perceived exertion (may overexert)
- Alters thermoregulation

**Smart Workout Strategies:**

- **Best for**: Moderate cardio, skill-based sports
- **Caution with**: HIIT, hot yoga, competitive sports
- **Pro Tips**:
  - Hydrate with electrolyte drinks (stimulants suppress thirst)
  - Wear core temperature monitor in heat
  - Time workouts before afternoon med crash
  - Consider shorter, more frequent sessions

**2. Non-Stimulants (Second-Line)**

**Common Options:**

- **Atomoxetine (Strattera)**: Takes 4-8 weeks for full effect
- **Alpha-2 Agonists**: Intuniv, Kapvay

**Exercise Advantages:**

- No cardiac overstimulation
- Better for endurance athletes
- No WADA restrictions

**Adaptation Period:**

- Week 1-2: May cause exercise fatigue
- Week 3-4: Energy normalizes
- Long-term: May improve workout consistency

**3. Off-Label Options**

- **Bupropion**: Avoid extreme endurance sports
- **Modafinil**: Minimal exercise impact

**Sport-Specific Recommendations**

| **Activity** | **Stimulant Users** | **Non-Stimulant Users** |
| --- | --- | --- |
| **Running** | Monitor HR zones; avoid heat | Ideal for marathon training |
| **Weightlifting** | Skip 1RM attempts; focus on reps | Better for powerlifting |
| **Swimming** | Great option (cool environment) | Excellent choice |
| **Team Sports** | Check league bans (many prohibit stimulants) | Fully permitted |

**Key Safety Protocols**

1. **Cardiac Screening**: Get ECG if planning intense training
2. **Hydration**: Weigh pre/post workout (replace 150% loss)
3. **Recovery**: Prioritize sleep (meds may disrupt REM)
4. **Nutrition**: Protein-rich post-workout (counters appetite suppression)

**When to Stop Exercise:**
 Chest tightness/pain
 Dizziness that persists after rest
 Heart palpitations
 Sudden extreme fatigue

**Pro Tip:** Regular exercise actually enhances medication effectiveness long-term by upregulating dopamine receptors. Even 20 minutes of brisk walking can boost focus.

**Need Personalized Advice? Share:**

1. Your current medication/dose
2. Typical workout routine
3. Any side effects you notice

I can help optimize your fitness plan for your specific treatment regimen!

**Times 10**

1. **What is ADHD?**

**ADHD (Attention-Deficit/Hyperactivity Disorder) is a neurodevelopmental condition characterized by persistent differences in brain structure and function that affect self-regulation.** It's not a behavior problem or lack of discipline—it's a biologically-based variation in how the brain manages attention, motivation, and impulse control that begins in childhood and often continues throughout life.

**Core Characteristics:**

1. **Executive Dysfunction**
   - The brain's "management system" struggles with:
     • Starting tasks (even when important)
     • Switching between activities
     • Remembering steps in processes
     • Managing time effectively
     • Regulating emotions
2. **Interest-Based Nervous System**
   - Exceptional focus on exciting tasks (hyperfocus)
   - Difficulty engaging with mundane but important tasks
   - Strong need for immediate rewards/consequences
3. **Neurological Basis**
   - Prefrontal cortex develops more slowly
   - Differences in dopamine/norepinephrine systems
   - Highly genetic (about 75-80% heritability)

**The ADHD Paradoxes:**

✔ Can hyperfocus for hours but can't pay bills
✔ Remembers obscure facts but forgets appointments
✔ Excellent in emergencies but overwhelmed by daily tasks
✔ Deeply creative but organizationally challenged

**Real-World Impact:**

**At Work/School:**

- Cyclical productivity (bursts of hyperfocus vs. paralysis)
- "Organized chaos" organizational systems
- Missed deadlines despite good intentions

**In Relationships:**

- Interrupting conversations (despite trying not to)
- Emotional outbursts followed by quick remorse
- Forgetting important dates despite caring deeply

**Physical Signs:**

- Constant fidgeting or restlessness
- Sensory sensitivities (to textures, sounds, etc.)
- Sleep problems (difficulty falling asleep/waking)

**ADHD Strengths:**

- **Creativity** and out-of-the-box thinking
- **Resilience** from years of adapting
- **Crisis management** skills
- **Observational skills** (noticing what others miss)

**Diagnosis & Management:**

**Requires:**

- Symptoms present since childhood
- Impairment in multiple life areas
- Ruling out other conditions

**Effective Approaches:**

- Medication (stimulants/non-stimulants)
- Behavioral strategies
- Accommodations
- Strength-based approaches

**Key Insight:** ADHD isn't an attention deficit—it's an **attention inconsistency**. The challenge isn't lacking focus, but controlling what to focus on. With proper understanding and support, ADHD can become a superpower rather than a limitation.

Would you like more on how symptoms differ between genders or age groups?

1. **What are its symptoms?**

**ADHD Symptoms: The Complete Picture**

ADHD symptoms manifest as **persistent patterns of dysregulation** in attention, behavior, and emotions. These aren't occasional lapses—they're chronic challenges that significantly impact daily life. Here's what it really looks like:

**1. Attention Dysregulation**

*Not inability to focus, but inability to control focus*

- **Hyperfocus**: Losing 4 hours researching a hobby while ignoring hunger/bathroom needs
- **Hypofocus**: Mind hopping between 3 thoughts while someone talks to you
- **Selective Attention**: Missing obvious details while noticing irrelevant ones
- **Sensory Distractibility**: Unable to filter out background noises/clutter

**2. Executive Dysfunction**

*When your brain's "CEO" is understaffed*

- **Task Paralysis**: Physically frozen before starting simple tasks
- **Working Memory Gaps**: Forgetting why you entered a room mid-stride
- **Time Blindness**: Chronic lateness despite multiple alarms
- **Disorganization**: "Pile systems" that make sense only to you

**3. Emotional Dysregulation**

*Feelings dialed up to maximum volume*

- **Rejection Sensitivity**: Physical pain from minor criticism
- **Emotional Tsunamis**: Explosive frustration over small inconveniences
- **Interest-Based Motivation**: Only productive when fascinated/panicked

**4. Physical Manifestations**

*How ADHD lives in your body*

- **Restless Body Syndrome**: Constant fidgeting, leg bouncing, skin picking
- **Sensory Issues**: Hating clothing tags or craving intense stimuli
- **Movement Coordination**: Frequent minor accidents ("I bruise on doorframes")

**The ADHD Paradoxes**

✔ Can hyperfocus for hours but can't do laundry
✔ Remembers obscure facts but forgets anniversaries
✔ Excellent in crises but overwhelmed by chores
✔ Deeply creative but chronically disorganized

**Gender & Age Differences**

**Women/Girls Often Experience:**

- Internal restlessness rather than hyperactivity
- Perfectionism masking struggles
- More likely diagnosed in adulthood

**Adults vs. Children:**

- Less physical hyperactivity → more mental restlessness
- Life responsibilities expose executive dysfunction
- Years of accumulated shame ("Why can't I just...?")

**Real-World Impact**

**At Work:**

- Missed deadlines despite all-nighters
- Bursts of brilliance between periods of stagnation
- Desk covered in "organized chaos"

**In Relationships:**

- Zoning out mid-conversation (despite caring)
- Emotional outbursts followed by deep remorse
- Forgetting plans but remembering random details

**Key Insight:** ADHD isn't about capability—it's about **inconsistent access** to capabilities. The same person might:

- Solve complex work problems
- Struggle to shower regularly
- Create amazing art
- Forget to eat all day

Would you like:

1. **Coping strategies** for specific symptoms?
2. How to **explain this to neurotypical people**?
3. Guidance on **professional assessment**?
4. **What medications are used for ADHD, and do they conflict with physical exercise?**

**ADHD Medications & Exercise: A Practical Guide**

**1. Stimulant Medications (First-Line Treatment)**

**Common Options:**

- **Methylphenidates**: Ritalin (short-acting), Concerta (long-acting), Focalin
- **Amphetamines**: Adderall (mixed salts), Vyvanse (prodrug), Dexedrine

**Exercise Interactions:**

| **Effect** | **Solution** | **Best For** |
| --- | --- | --- |
| ↑ Heart rate (5-15 bpm) | Monitor with fitness tracker | Moderate cardio |
| ↑ Blood pressure | Avoid heavy lifting/HIIT | Swimming, cycling |
| ↓ Thirst perception | Set hydration reminders | Indoor workouts |
| ↑ Heat intolerance | Exercise in cool environments | Morning/evening sessions |
| Energy crashes | Time workouts before meds peak or taper | Short, intense sessions |

**Pro Tip:** Vyvanse is often preferred for athletes due to smoother effects and fewer anti-doping restrictions.

**2. Non-Stimulant Options**

**Common Choices:**

- **Atomoxetine (Strattera)**: Takes 4-8 weeks for full effect
- **Guanfacine (Intuniv)**: Originally for high blood pressure

**Exercise Advantages:**

- No cardiovascular overstimulation
- Better for endurance training
- No WADA anti-doping restrictions

**Watch For:**

- Early fatigue (Strattera) → Start with low-intensity workouts
- Dizziness (Guanfacine) → Move slowly between positions

**3. Off-Label Options**

- **Bupropion**: Caution with extreme endurance sports
- **Modafinil**: Minimal exercise impact but weaker ADHD effects

**Exercise Recommendations by Sport**

| **Activity** | **Stimulant Users** | **Non-Stimulant Users** |
| --- | --- | --- |
| **Running** | Monitor HR; avoid heat | Ideal for distance |
| **Weightlifting** | Skip 1-rep max attempts | Safer for heavy lifts |
| **Yoga** | Avoid hot styles | Great for stress relief |
| **Team Sports** | Check league bans | Generally unrestricted |

**Key Safety Tips**

1. **Hydration**: Drink electrolyte solutions (stimulants suppress thirst)
2. **Timing**: Exercise before medication peaks or as it tapers
3. **Recovery**: Prioritize sleep (meds may disrupt REM cycles)
4. **Nutrition**: Protein-rich post-workout snacks counter appetite suppression

**Red Flags to Stop:**
 Chest pain or irregular heartbeat
 Dizziness that persists after rest
Sudden extreme fatigue
Excessive post-workout crash

**Pro Insight:** Regular exercise actually enhances medication effectiveness long-term by improving dopamine receptor sensitivity. Even 10 minutes of activity can boost focus.

**Want Personalized Advice? Share:**

1. Your specific medication/dose
2. Typical workout routine
3. Any side effects you notice

I can help tailor an exercise plan that works with your treatment!
