# Supplementary material for: Evaluating large language models for ADHD education: A comparative study of ChatGPT 5, DeepSeek V3, and Grok 4
Source: PLoS One. 2026 Jun 1;21(6):e0335335. doi: 10.1371/journal.pone.0335335 (PMC13225419; doi:10.1371/journal.pone.0335335)
Supplement: S2 File — This file contains the structured quantitative data used for statistical analysis, including individual scores for readability indices (FKRE, FKGL, SMOG, etc.), lexical complexity metrics, and qualitative error coding for each model adherence to DSM-5 guidelines and additional content reporting across all iterations. (DOCX) [file pone.0335335.s002.docx]

**Table S1. Minimal dataset for readability, lexical complexity, and content-coding analyses.**

| **Question** | **Model** | **Times** | **Flesch Kincaid Reading Ease** | **Flesch Kincaid Grade Level** | **Gunning Fog Score** | **Simple Measure of Gobbledygook Index** | **Coleman Liau Index** | **Sentences** | **Words** | **Complex words** | **Percent of complex words** | **Average words per sentence** | **Citations Included (Yes/ No)** | **Factual Errors** | **Error comments** | **Comments** | **Omission (e.g., Differential Diagnosis/ Comorbidities)** | **Adherence to Latest Guidelines (e.g.,DSM-5-TR)** | **Comments** | **Additional Content reported** |
| --- | --- | --- | --- | --- | --- | --- | --- | --- | --- | --- | --- | --- | --- | --- | --- | --- | --- | --- | --- | --- |
| Q1 | ChatGPT 5 | 1 | 31.7 | 12.3 | 13.3 | 10.7 | 16.8 | 16 | 221 | 54 | 24.43% | 13.81 | No | 0 | 0 | 0 | 0 | No | Partially followed DSM-4 and DSM-5 | Reported ADHD onset and causal factors. |
| Q1 | ChatGPT 5 | 2 | 21.5 | 14.5 | 16.6 | 12.6 | 18.2 | 12 | 218 | 57 | 26.15% | 18.17 | No | 0 | 0 | 0 | 0 | No | Followed DSM-4 | How It Works in the Brain |
| Q1 | ChatGPT 5 | 3 | 24.5 | 15.4 | 17.5 | 13.5 | 17.2 | 9 | 202 | 49 | 24.26% | 22.44 | No | 0 | 0 | 0 | 0 | No | Followed DSM-4 | 0 |
| Q1 | ChatGPT 5 | 4 | 14.9 | 16.6 | 18.1 | 13.9 | 18.7 | 9 | 198 | 52 | 26.26% | 22 | No | 0 | 0 | 0 | 0 | No | Followed DSM-4 | Across the lifespan |
| Q1 | ChatGPT 5 | 5 | 19.7 | 14.5 | 15.4 | 12 | 19.4 | 10 | 161 | 43 | 26.71% | 16.1 | No | 0 | 0 | 0 | 0 | No | Followed DSM-4 | 0 |
| Q1 | ChatGPT 5 | 6 | 18.6 | 14.8 | 16 | 12.4 | 19.2 | 10 | 168 | 46 | 27.38% | 16.8 | No | 0 | 0 | 0 | 0 | No | Followed DSM-4 | 0 |
| Q1 | ChatGPT 5 | 7 | 18.4 | 14.9 | 16.2 | 12.4 | 18.7 | 10 | 170 | 46 | 27.06% | 17 | No | 0 | 0 | 0 | 0 | No | Followed DSM-4 | 0 |
| Q1 | ChatGPT 5 | 8 | 6.9 | 18.6 | 20.1 | 15.6 | 18.1 | 6 | 152 | 44 | 28.95% | 25.33 | No | 0 | 0 | 0 | 0 | No | 0 | 0 |
| Q1 | ChatGPT 5 | 9 | -12.6 | 25.3 | 27 | 20.5 | 19.5 | 4 | 166 | 51 | 30.72% | 41.5 | No | 0 | 0 | 0 | 0 | No | 0 | 0 |
| Q1 | ChatGPT 5 | 10 | -18 | 30.1 | 32.5 | 23.4 | 17.3 | 3 | 173 | 50 | 28.90% | 57.67 | No | 0 | 0 | 0 | 0 | No | 0 | 0 |
| Q2 | ChatGPT 5 | 1 | 20.8 | 18 | 19.3 | 14.8 | 16 | 10 | 307 | 66 | 21.43% | 30.8 | No | 0 | 0 | 0 | 0 | Yes | 0 | Emotional & Executive Function Challenges |
| Q2 | ChatGPT 5 | 2 | 17.8 | 18.8 | 18.8 | 14.5 | 16.2 | 8 | 259 | 66 | 21.50% | 30.7 | No | 0 | 0 | 0 | 0 | Yes | 0 | Common but Less-Official Challenges |
| Q2 | ChatGPT 5 | 3 | 23.7 | 16.7 | 16.7 | 13.3 | 16.1 | 10 | 270 | 51 | 19.69% | 32.38 | No | 0 | 0 | 0 | 0 | Yes | 0 | Common “Unofficial” Challenges |
| Q2 | ChatGPT 5 | 4 | 27.2 | 16.4 | 16.6 | 13 | 15.3 | 10 | 281 | 53 | 19.63% | 27 | No | 0 | 0 | 0 | 0 | Yes | 0 | 0 |
| Q2 | ChatGPT 5 | 5 | 26.9 | 16.5 | 16.5 | 13.2 | 15.3 | 10 | 281 | 75 | 24.19% | 28.18 | No | 0 | 0 | 0 | 0 | Yes | 0 | 0 |
| Q2 | ChatGPT 5 | 6 | 11.6 | 21.1 | 28.5 | 15.5 | 16.3 | 7 | 267 | 51 | 19.10% | 38.14 | No | 0 | 0 | 0 | 0 | Yes | 0 | 0 |
| Q2 | ChatGPT 5 | 7 | 12.9 | 21.2 | 21 | 15.7 | 15.6 | 7 | 276 | 52 | 18.84% | 39.43 | No | 0 | 0 | 0 | 0 | Yes | 0 | 0 |
| Q2 | ChatGPT 5 | 8 | 10.4 | 23.1 | 23.4 | 16.1 | 15.3 | 6 | 274 | 47 | 17.15% | 45.67 | No | 0 | 0 | 0 | 0 | Yes | 0 | 0 |
| Q2 | ChatGPT 5 | 9 | 11.5 | 22.7 | 23.3 | 15.9 | 14.8 | 6 | 267 | 46 | 17.23% | 44.5 | No | 0 | 0 | 0 | 0 | Yes | 0 | 0 |
| Q2 | ChatGPT 5 | 10 | 12.1 | 22.5 | 23.2 | 16.3 | 14.7 | 6 | 263 | 48 | 18.25% | 43.83 | No | 0 | 0 | 0 | 0 | Yes | 0 | 0 |
| Q3 | ChatGPT 5 | 1 | 34.4 | 13.1 | 15.2 | 12.5 | 15.8 | 20 | 372 | 94 | 25.27% | 18.6 | No | 0 | 0 | 0 | 0 | Yes | 0 | 0 |
| Q3 | ChatGPT 5 | 2 | 29.3 | 16.5 | 17.8 | 14.6 | 14 | 11 | 323 | 71 | 21.98% | 29.36 | No | 0 | 0 | 0 | 0 | Yes | 0 | Key safety points |
| Q3 | ChatGPT 5 | 3 | 28.7 | 16.4 | 17.5 | 14.6 | 14 | 11 | 317 | 71 | 22.40% | 28.82 | No | 0 | 0 | 0 | 0 | Yes | 0 | 0 |
| Q3 | ChatGPT 5 | 4 | 26.8 | 16.5 | 18 | 15 | 14.4 | 11 | 310 | 69 | 23.00% | 27.27 | No | 0 | 0 | 0 | 0 | Yes | 0 | 0 |
| Q3 | ChatGPT 5 | 5 | 28.6 | 16.1 | 17 | 14.4 | 14.4 | 11 | 300 | 81 | 25.47% | 28.91 | No | 0 | 0 | 0 | 0 | Yes | 0 | 0 |
| Q3 | ChatGPT 5 | 6 | 22.4 | 17.3 | 19 | 15.6 | 15.2 | 11 | 318 | 78 | 23.64% | 30 | No | 0 | 0 | 0 | 0 | Yes | 0 | 0 |
| Q3 | ChatGPT 5 | 7 | 24.4 | 17.3 | 18.8 | 15.3 | 15 | 11 | 330 | 73 | 23.62% | 28.09 | No | 0 | 0 | 0 | 0 | Yes | 0 | 0 |
| Q3 | ChatGPT 5 | 8 | 23.9 | 16.9 | 17.7 | 14.8 | 15.6 | 11 | 309 | 76 | 24.52% | 34.44 | No | 0 | 0 | 0 | 0 | Yes | 0 | 0 |
| Q3 | ChatGPT 5 | 9 | 20.1 | 19 | 20.5 | 16.7 | 14.9 | 9 | 310 | 76 | 24.36% | 34.67 | No | 0 | 0 | 0 | 0 | Yes | 0 | 0 |
| Q3 | ChatGPT 5 | 10 | 15.9 | 20 | 21.4 | 17.1 | 15.3 | 9 | 326 | 80 | 24.54% | 36.22 | No | 0 | 0 | 0 | 0 | Yes | 0 | 0 |
| Q1 | DeepSeek V3 | 1 | 32.5 | 10.6 | 10.3 | 8.2 | 17.3 | 65 | 479 | 128 | 26.72% | 7.37 | Yes | 0 | 0 | 0 | 0 | Yes | 0 | Myths vs. Facts; Support & Resources: |
| Q1 | DeepSeek V3 | 2 | 23.8 | 12.3 | 12.4 | 9.9 | 18 | 53 | 500 | 153 | 30.60% | 9.43 | No | 0 | 0 | 0 | 0 | Yes | 0 | Why It Matters |
| Q1 | DeepSeek V3 | 3 | -4 | 19.8 | 18.9 | 15.9 | 20.4 | 10 | 239 | 76 | 31.80% | 23.9 | No | 1 | No Diagnostic Criteria | 0 | 0 | No | No Presentations | 0 |
| Q1 | DeepSeek V3 | 4 | -10.9 | 24.8 | 24.9 | 20.3 | 18.7 | 6 | 241 | 75 | 31.12% | 40.17 | No | 1 | No Diagnostic Criteria | 0 | 0 | Yes | 0 | The Paradox: |
| Q1 | DeepSeek V3 | 5 | -2.8 | 21.3 | 20.8 | 17.1 | 20.3 | 9 | 277 | 80 | 28.88% | 30.78 | No | 1 | No Diagnostic Criteria | 0 | 0 | No | No Presentations | 0 |
| Q1 | DeepSeek V3 | 6 | -0.9 | 21.3 | 21.4 | 17.9 | 19.7 | 10 | 320 | 97 | 30.31% | 32 | No | 1 | No Diagnostic Criteria | 0 | 0 | No | No Presentations | ADHD Symptoms Beyond Stereotypes |
| Q1 | DeepSeek V3 | 7 | 17.4 | 17.3 | 18.2 | 14.9 | 17.4 | 15 | 389 | 101 | 25.96% | 25.93 | No | 1 | No Diagnostic Criteria | 0 | 0 | No | No Presentations | The ADHD Paradoxes |
| Q1 | DeepSeek V3 | 8 | 10.9 | 18.1 | 18.2 | 15.2 | 18.7 | 15 | 385 | 104 | 27.01% | 25.67 | No | 1 | No Diagnostic Criteria | 0 | 0 | No | 0 | 0 |
| Q1 | DeepSeek V3 | 9 | 10.1 | 18 | 18.4 | 15.4 | 19.6 | 15 | 374 | 107 | 28.61% | 24.93 | No | 1 | No Diagnostic Criteria | 0 | 0 | No | 0 | 0 |
| Q1 | DeepSeek V3 | 10 | 4.1 | 20.5 | 20.1 | 17.2 | 18.9 | 11 | 345 | 99 | 28.07% | 31.36 | No | 1 | No Diagnostic Criteria | 0 | 0 | No | 0 | 0 |
| Q2 | DeepSeek V3 | 1 | 36.1 | 10.3 | 10.1 | 8.3 | 17.9 | 45 | 381 | 91 | 23.88% | 8.47 | No | 0 | 0 | 0 | 0 | Yes | 0 | ADHD Symptoms in Adults; When to Seek Help |
| Q2 | DeepSeek V3 | 2 | 42.4 | 9.8 | 10.2 | 8.5 | 16 | 46 | 450 | 97 | 21.56% | 9.78 | No | 0 | 0 | 0 | 0 | No | ADHD Subtypes | How Symptoms Differ by Age(Table);Real-Life Impact |
| Q2 | DeepSeek V3 | 3 | -6.6 | 24 | 23.4 | 19.2 | 20.2 | 9 | 356 | 101 | 28.37% | 39.56 | No | 0 | 0 | 0 | 0 | Yes | 0 | Gender Differences; Life Stage Variations |
| Q2 | DeepSeek V3 | 4 | 27 | 16.7 | 17 | 14.4 | 15.3 | 14 | 406 | 88 | 21.67% | 29 | No | 0 | 0 | 0 | 0 | Yes | 0 | Gender & Age Differences; Key Insight: ADHD isn't about can't but can't consistently. |
| Q2 | DeepSeek V3 | 5 | 34.5 | 16.4 | 16.9 | 13.4 | 12.7 | 13 | 417 | 70 | 16.79% | 32.08 | No | 0 | 0 | 0 | 0 | No | No Presentations | The ADHD Iceberg |
| Q2 | DeepSeek V3 | 6 | 41.7 | 11.9 | 12.2 | 10.7 | 13.8 | 26 | 469 | 88 | 18.76% | 18.04 | No | 0 | 0 | 0 | 0 | No | No Presentations | Real-World Impact |
| Q2 | DeepSeek V3 | 7 | 20.7 | 16.8 | 16.2 | 14.4 | 16.7 | 14 | 362 | 88 | 24.31% | 25.86 | No | 0 | 0 | 0 | 0 | No | No Presentations | Key Insight: ADHD symptoms represent a mismatch between brain wiring and environmental demands, not a lack of capability. |
| Q2 | DeepSeek V3 | 8 | 38.4 | 13.3 | 13.8 | 11.8 | 13.5 | 21 | 456 | 87 | 19.08% | 21.71 | No | 0 | 0 | 0 | 0 | No | 0 | 0 |
| Q2 | DeepSeek V3 | 9 | 36.8 | 13.9 | 13.6 | 11.9 | 14.1 | 19 | 442 | 80 | 18.10% | 23.26 | No | 0 | 0 | 0 | 0 | No | 0 | 0 |
| Q2 | DeepSeek V3 | 10 | 14.1 | 17.9 | 17.2 | 15.2 | 17.5 | 15 | 398 | 105 | 26.38% | 26.53 | No | 0 | 0 | 0 | 0 | No | 0 | 0 |
| Q3 | DeepSeek V3 | 1 | 33.9 | 11.6 | 12 | 10.8 | 15.5 | 35 | 437 | 122 | 27.92% | 12.49 | No | 0 | 0 | 0 | 0 | Yes | 0 | General Tips for Exercising on ADHD Meds |
| Q3 | DeepSeek V3 | 2 | 34.1 | 10.8 | 9.9 | 9.5 | 16.8 | 42 | 395 | 111 | 28.10% | 9.4 | No | 0 | 0 | 0 | 0 | Yes | 0 | Exercise Recommendations by Medication Type(Table);Final Advice: |
| Q3 | DeepSeek V3 | 3 | -22.1 | 27.5 | 23.8 | 23.3 | 19.1 | 2 | 90 | 33 | 36.67% | 45 | No | 0 | 0 | 0 | 0 | Yes | 0 | Optimizing Your Routine |
| Q3 | DeepSeek V3 | 4 | 14.3 | 18.2 | 16.6 | 16.3 | 18.2 | 5 | 139 | 40 | 28.78% | 27.8 | No | 0 | 0 | 0 | 0 | Yes | 0 | Exercise Recommendations by Sport(Table) |
| Q3 | DeepSeek V3 | 5 | 17.6 | 18.6 | 17.9 | 16.3 | 17.5 | 5 | 157 | 40 | 25.48% | 31.4 | No | 0 | 0 | 0 | 0 | Yes | 0 | ⚠ Risks: Exercise Recommendations by Sport(Table) |
| Q3 | DeepSeek V3 | 6 | 22.7 | 14.6 | 13.6 | 13.6 | 17.9 | 16 | 291 | 89 | 30.58% | 18.19 | No | 0 | 0 | 0 | 0 | Yes | 0 | Pro Tip: Moderate cardio (jogging, swimming) is generally safer than extreme endurance sports. |
| Q3 | DeepSeek V3 | 7 | -0.3 | 23.9 | 21.4 | 20.2 | 15.8 | 5 | 214 | 62 | 28.97% | 42.8 | No | 0 | 0 | 0 | 0 | Yes | 0 | 0 |
| Q3 | DeepSeek V3 | 8 | 17.3 | 18.5 | 15.5 | 16 | 16.5 | 7 | 216 | 54 | 25.00% | 30.86 | No | 0 | 0 | 0 | 0 | Yes | 0 | 0 |
| Q3 | DeepSeek V3 | 9 | 23.5 | 16.4 | 16.3 | 15.1 | 14.8 | 14 | 360 | 97 | 26.94% | 25.71 | No | 0 | 0 | 0 | 0 | Yes | 0 | 0 |
| Q3 | DeepSeek V3 | 10 | 20.8 | 16.1 | 14.3 | 14.3 | 17 | 15 | 346 | 93 | 26.88% | 23.07 | No | 0 | 0 | 0 | 0 | Yes | 0 | 0 |
| Q1 | Grok 4 | 1 | 13.8 | 14.7 | 15.2 | 11.3 | 21.3 | 28 | 309 | 106 | 34.30% | 11.04 | No | 0 | 0 | 0 | 0 | Yes | 0 | 0 |
| Q1 | Grok 4 | 2 | 17.8 | 14.4 | 17.6 | 12.7 | 19.7 | 13 | 192 | 63 | 32.81% | 14.77 | No | 0 | 0 | 0 | 0 | Yes | 0 | 0 |
| Q1 | Grok 4 | 3 | 13.5 | 14.2 | 15.3 | 11.2 | 20.9 | 26 | 293 | 98 | 33.45% | 11.27 | No | 0 | 0 | 0 | 0 | Yes | 0 | 0 |
| Q1 | Grok 4 | 4 | 9.6 | 15.7 | 17 | 12.8 | 21.2 | 19 | 288 | 93 | 32.29% | 15.16 | No | 0 | 0 | 0 | 0 | Yes | 0 | 0 |
| Q1 | Grok 4 | 5 | 7.2 | 14.9 | 15.6 | 11.4 | 22.1 | 25 | 268 | 97 | 36.19% | 10.72 | No | 0 | 0 | 0 | 0 | Yes | 0 | 0 |
| Q1 | Grok 4 | 6 | 14.2 | 13.6 | 14.8 | 10.4 | 21.3 | 29 | 276 | 93 | 33.70% | 9.52 | No | 0 | 0 | 0 | 0 | Yes | 0 | 0 |
| Q1 | Grok 4 | 7 | 21.1 | 12.8 | 14.4 | 10.4 | 19.5 | 24 | 244 | 77 | 31.56% | 10.17 | No | 0 | 0 | 0 | 0 | Yes | 0 | 0 |
| Q1 | Grok 4 | 8 | 16.6 | 14.3 | 16.9 | 12 | 20.1 | 17 | 229 | 73 | 31.88% | 13.47 | No | 0 | 0 | 0 | 0 | Yes | 0 | 0 |
| Q1 | Grok 4 | 9 | 14.6 | 14.8 | 17.3 | 12.4 | 19.8 | 14 | 201 | 64 | 31.84% | 14.36 | No | 0 | 0 | 0 | 0 | Yes | 0 | 0 |
| Q1 | Grok 4 | 10 | 8.1 | 15.9 | 18.5 | 13.2 | 21.4 | 13 | 195 | 68 | 34.87% | 15 | No | 0 | 0 | 0 | 0 | Yes | 0 | 0 |
| Q2 | Grok 4 | 1 | 33.4 | 11.9 | 13.7 | 10.5 | 17.7 | 41 | 540 | 133 | 24.63% | 13.17 | No | 0 | 0 | 0 | 0 | Yes | 0 | Examples in Daily Life: |
| Q2 | Grok 4 | 2 | 39.4 | 9.7 | 12.4 | 8 | 17 | 33 | 251 | 62 | 24.70% | 7.61 | No | 0 | 0 | 0 | 0 | Yes | 0 | 0 |
| Q2 | Grok 4 | 3 | 35.9 | 10.6 | 13.6 | 9.2 | 17.7 | 47 | 448 | 116 | 25.89% | 9.53 | No | 0 | 0 | 0 | 0 | Yes | 0 | 0 |
| Q2 | Grok 4 | 4 | 27.8 | 13.2 | 14.7 | 11.4 | 18.5 | 34 | 520 | 133 | 25.58% | 15.29 | No | 0 | 0 | 0 | 0 | Yes | 0 | 0 |
| Q2 | Grok 4 | 5 | 28.1 | 12.2 | 13.3 | 10.2 | 18.9 | 44 | 502 | 136 | 27.09% | 11.41 | No | 0 | 0 | 0 | 0 | Yes | 0 | Disclaimer: Grok is not a doctor; please consult one. Don't share information that can identify you.0 |
| Q2 | Grok 4 | 6 | 47.1 | 8.2 | 11.1 | 7 | 15.9 | 55 | 343 | 76 | 22.16% | 6.24 | No | 0 | 0 | 0 | 0 | Yes | 0 | 0 |
| Q2 | Grok 4 | 7 | 31.4 | 12.2 | 14.7 | 10.8 | 18.3 | 37 | 492 | 128 | 26.02% | 13.3 | No | 0 | 0 | 0 | 0 | Yes | 0 | 0 |
| Q2 | Grok 4 | 8 | 31.5 | 10.7 | 13 | 8.2 | 18.7 | 43 | 326 | 85 | 26.07% | 7.58 | No | 0 | 0 | 0 | 0 | Yes | 0 | 0 |
| Q2 | Grok 4 | 9 | 30.3 | 12.4 | 13 | 10.5 | 18.1 | 26 | 353 | 85 | 24.08% | 13.58 | No | 0 | 0 | 0 | 0 | Yes | 0 | 0 |
| Q2 | Grok 4 | 10 | 32.9 | 11.2 | 14.2 | 9.6 | 17.7 | 42 | 426 | 115 | 10.14% | 14.64 | No | 0 | 0 | 0 | 0 | Yes | 0 | 0 |
| Q3 | Grok 4 | 1 | 26.9 | 12.4 | 15 | 11.1 | 18.9 | 86 | 993 | 313 | 31.52% | 11.55 | No | 0 | 0 | 0 | 0 | Yes | 0 | 0 |
| Q3 | Grok 4 | 2 | 24.8 | 12.1 | 15.4 | 10.4 | 19.7 | 64 | 597 | 205 | 34.34% | 11.25 | No | 0 | 0 | 0 | 0 | Yes | 0 | 0 |
| Q3 | Grok 4 | 3 | 17.9 | 14.7 | 16.9 | 13 | 19.6 | 48 | 757 | 245 | 32.36% | 15.77 | No | 0 | 0 | 0 | 0 | Yes | 0 | Additional Notes |
| Q3 | Grok 4 | 4 | 22.6 | 13.6 | 15.9 | 12.1 | 19 | 81 | 1145 | 353 | 30.83% | 14.14 | No | 0 | 0 | 0 | 0 | Yes | 0 | Insights from Recent Discussions |
| Q3 | Grok 4 | 5 | 19.6 | 13.9 | 16.7 | 12.3 | 19.4 | 76 | 1040 | 346 | 33.27% | 13.68 | No | 0 | 0 | 0 | 0 | Yes | 0 | General Recommendations: |
| Q3 | Grok 4 | 6 | 25.8 | 12.6 | 14.8 | 11.1 | 18.9 | 76 | 904 | 278 | 30.75% | 11.89 | No | 0 | 0 | 0 | 0 | Yes | 0 | 2025 Context |
| Q3 | Grok 4 | 7 | 22.9 | 13.4 | 15.8 | 12.1 | 19.5 | 53 | 721 | 232 | 32.18% | 13.6 | No | 0 | 0 | 0 | 0 | Yes | 0 | 0 |
| Q3 | Grok 4 | 8 | 22.7 | 13.2 | 15.7 | 11.5 | 19.5 | 75 | 925 | 296 | 32.00% | 12.33 | No | 0 | 0 | 0 | 0 | Yes | 0 | 0 |
| Q3 | Grok 4 | 9 | 22.4 | 13.2 | 15.2 | 11.5 | 19.3 | 72 | 898 | 284 | 31.63% | 12.47 | No | 0 | 0 | 0 | 0 | Yes | 0 | 0 |
| Q3 | Grok 4 | 10 | 21.5 | 13.7 | 16.3 | 12.3 | 19.1 | 71 | 988 | 324 | 32.79% | 13.92 | No | 0 | 0 | 0 | 0 | Yes | 0 | 0 |
