# Supplementary material for: Evaluating large language models for ADHD education: A comparative study of ChatGPT 5, DeepSeek V3, and Grok 4
Source: PLoS One. 2026 Jun 1;21(6):e0335335. doi: 10.1371/journal.pone.0335335 (PMC13225419; doi:10.1371/journal.pone.0335335)
Supplement: Attachment — Submitted filename: Response to Reviewers.docx [file pone.0335335.s004.docx]

Response to editors:

Thank you very much for your time and comments. We have carefully addressed all issues raised by the editor and reviewers and revised the manuscript accordingly. Our point-by-point responses are provided as follows:

**Response to Review #1:**

**Comment1:** Interesting and provocative paper. Some issues.

Thank you very much for your time and comments. We have revised the entire text in accordance with your valuable suggestions. Our responses to comments as a follows:

**Comment 2:** Abstract: primary end point should be added

**Response:** Thank you for this suggestion. The Abstract has been revised to clearly state the primary endpoints of the study, namely content accuracy, readability, and response stability. This information has been added to the Methods section of the Abstract. The change has been highlighted in the revised manuscript.

**Revision in manuscript**: The primary endpoint has been added to the Abstract (Lines 24–28):

*“The primary endpoints were content accuracy, readability, and response stability. Readability was evaluated using the Flesch–Kincaid Reading Ease score (FKRE), the Flesch–Kincaid Grade Level (FKGL), and the Simple Measure of Gobbledygook (SMOG), alongside measures of lexical complexity.”*

**Comment 3:** Definition of high reading level should be added

**Response**: Thank you for this suggestion. We completely agree that explicitly defining a “high reading level” provides better clarity for the readers. To address this, we have clarified this definition in the revised manuscript by contrasting our models' performance with the commonly recommended public health readability benchmarks. The additions has been added to the abstract and methods:

**Revision in manuscript**:

**Abstract**: We added the specific thresholds that define the high reading levels observed in our study (Lines 33–35):

*“all models exhibited high reading levels (FKGL > 12, FKRE < 40, SMOG > 12), exceeding recommended public-health readability standards for general audiences, which are typically around Grade 6-8.”*

Results (Readability): We added the baseline definition of what is considered an acceptable reading level to provide clear context (Lines 161-163)：

*“In general, FKRE scores above 60 indicate acceptable readability, while FKGL and SMOG scores of 6 or below are considered suitable for the general population [1].”*

**Comment 4:** Introduction for a paper like this should be shortened to 1 page

**Response:** Thank you for this helpful suggestion. We have done our best to streamline the Introduction, reducing it to approximately one page. This revision has made the background context more focused and the entire manuscript more concise and clearer.

**Comment 5:** Stat Methods: normal distributions should be checked for

**Response:** Thank you for this helpful comment. In the revised manuscript, we have added a statement in the Data Analysis section clarifying that the distribution of quantitative variables was examined using the Shapiro Wilk test prior to statistical analysis. As the study primarily aimed to provide descriptive comparisons across models and prompts, the results are presented mainly using descriptive statistics.

**Revision in manuscript**: Normality testing has been added in the Data Analysis section (Lines 171-173) :

*“Prior to statistical analysis, the distribution of quantitative variables was assessed using the Shapiro Wilk test to examine normality.”*

**Comment 6:** Methods: it is not clear who reviewed the models

**Response:** Thank you for this comment. The Methods section has been revised to clarify the evaluation procedure. The manuscript now specifies that two independent reviewers (XMH and MZ) evaluated all model responses according to predefined content categories, and any discrepancies were resolved through discussion with a third researcher (RRX).

**Revision in manuscript:** This clarification has been added in the Quality Analysis subsection (Lines 138-141):

*“Two independent reviewers (XMH and MZ) evaluated all responses using this predefined evaluation framework. Each reviewer assessed the responses separately. Discrepancies between reviewers were resolved through discussion with a third researcher (RRX) until consensus was reached.”*

**Comment 7:** Methods: do these results of LLM predicted also response to therapies?

**Response:** Thank you for this comment. The aim of this study was to evaluate the informational quality and readability of ADHD-related responses generated by large language models. The study did not assess whether LLMs can predict treatment responses or evaluate the clinical effectiveness of therapies. But we agree that this is an important area for future research. Therefore, we have added a statement in the limitations section to acknowledge that future studies should examine whether LLM-generated information aligns with evidence-based treatment recommendations and whether it has potential value in supporting treatment-related decision-making.

**Revision in manuscript:** A statement clarifying this point has been added to the limitations section（Lines 366-373）:

*“A further limitation is that this study did not assess whether LLM-generated responses can predict treatment outcomes or reflect the effectiveness of therapies. Future research should examine the extent to which LLM-generated information aligns with evidence-based treatment recommendations and whether it may have value in supporting treatment-related decision-making. Finally, the study focused primarily on textual readability and stability, rather than evaluating real-world comprehension or decision-making by specific user groups such as PE teachers.”*

**Response to Review #2:**

The study is promising, but clearer methodological rigor, stronger grounding in ADHD education literature, and deeper comparative analysis are needed.

**Comment 1:** Provide the exact prompt set, prompt order, temperature or generation settings, and session controls to ensure reproducibility and fairness across models.

**Response:** Thank you for this helpful suggestion. We have revised the Methods section to provide additional details regarding reproducibility. The manuscript now specifies the exact prompt set, the prompt order used in the experiment, and the session controls applied to minimize contextual carryover between prompts. We also clarify that all responses were generated using the default generation settings of each platform, with no manual adjustments to parameters such as temperature or sampling strategies.

**Revision in manuscript:** Additional methodological details have been added to the Procedure sections (Lines 121-127)：

***“****To ensure reproducibility and fairness across models, the exact prompt set and prompt order were kept identical for all models. The prompts were entered sequentially in the following order: Q1, Q2, and Q3. All responses were generated using the default generation parameters provided by each platform. No manual adjustments were made to parameters such as temperature, top-p, or other sampling settings. Each prompt was submitted in a new chat session after clearing the browser cache to minimize contextual carryover. The same device and internet environment were used for all tests.”*

**Comment 2:** Clarify how “content accuracy” was evaluated—who rated it, using which rubric, and whether inter-rater agreement was measured.

**Response:** Thank you for this helpful suggestion. In the revised manuscript, we have clarified the evaluation procedure in the Methods section. Content accuracy was assessed based on consistency with the DSM-5-TR diagnostic criteria and clinical descriptions of ADHD. Two independent reviewers (XMH and MZ) evaluated all responses using this predefined framework, and any discrepancies were resolved through discussion with a third researcher (RRX) until consensus was reached. This clarification improves the transparency and rigor of the evaluation process.

**Revision in manuscript:** Additional details have been added in the Quality Analysis subsection of the Methods (Lines 133-141):

***“****Content accuracy was evaluated based on consistency with the diagnostic criteria and clinical descriptions outlined in the DSM-5-TR (Diagnostic and Statistical Manual of Mental Disorders, Fifth Edition, Text Revision). Specifically, responses were assessed for the correctness and completeness of information related to ADHD definition, symptoms, and commonly used treatments.*

*Two independent reviewers (XMH and MZ) evaluated all responses using this predefined evaluation framework. Each reviewer assessed the responses separately. Discrepancies between reviewers were resolved through discussion with a third researcher (RRX) until consensus was reached.”*

**Comment 3:** Expand the literature review on ADHD inclusion in physical education and coaching contexts, including established accessibility and universal design frameworks.

**Response:** Thank you for this helpful suggestion. We have expanded the literature review to provide a clearer overview of inclusive physical education and relevant accessibility considerations for students with ADHD. However, as the main focus of this study was to examine the feasibility of using large language models to support ADHD-related knowledge acquisition among non-specialist educators, especially outsourced coaches and casual physical education instructors, the introduction was intentionally structured to remain focused on the specific scope and practical purpose of the study.

**Revision in manuscript:** The literature review has been expanded in the Introduction (Lines 44-51,52-54, 56-58, 61-63, 65-66, 72-76,80-81)：

*“In recent years, increasing attention has been paid to inclusive physical education, which aims to ensure that students with diverse learning and behavioural needs can participate meaningfully in physical activity. Inclusive PE emphasizes adapting instructional strategies, activity structures, and classroom management approaches to support students with neurodevelopmental conditions such as ADHD. Studies on attentional neurodiversity in physical education suggest that effective inclusion often relies on flexible teaching strategies, differentiated instruction, and supportive peer environments that facilitate both participation and social interaction [2].”*

***“****ADHD is one of the most prevalent neurodevelopmental disorders among children worldwide [3]. It is characterized by persistent patterns of inattention, hyperactivity, and impulsivity [4].”*

***“****Children with ADHD may face difficulties participating in paganize sports and group based activities.****”***

*“The competence of outsourced coaches, particularly their knowledge of ADHD, has been questioned [5].”*

*“LLMs are increasingly used to support teachers, clinicians, and parents in understanding developmental disorders.”*

*“Therefore readability, accuracy, and response stability are critical when AI generated materials are used by non-specialist educators. However, little research has systematically examined the readability and reliability of LLM-generated ADHD-related content for this population of educators.”*

*“Different LLMs may produce response with varying clarity, structure, and accessibility.”*

**Comment 4:** While the study provides a useful comparison of LLM-generated ADHD educational material, the related work would benefit from referencing existing research on AI-based clinical decision support and automated interpretation of physiological data. This will provide relevant insights into the challenges of accuracy, stability, and accessibility when AI systems are used to communicate health-related information to non-expert users. I suggest authors consider citing; 'Spirometer and sEMG respiratory patterns for clinical decision support system'. ( Doi: 10.1109/I2MTC53148.2023.10175925 )

**Response:** Thank you for this helpful suggestion. The recommended reference has been cited in the Discussion section.

**Revision in manuscript:** we have added a comparison with comparison with previous studies (Line 301-303):

*“Despite these advancements, the results also expose significant challenges. Consistent with earlier studies, we found that readability remains a key obstacle to the real-world use of LLM generated materials [6, 7].”*

**Comment 5:** Include statistical testing (e.g., variance analysis, confidence intervals, or effect sizes) to support claims about stability and differences between models.

**Response:** Thank you for your comments. 95% CI for readability and complexity are provided in the results section.

**Revision in manuscript:** We have added specific examples, such as readability and complexity (Line 268-271)：

***“****Although its scores were consistently the lowest, with FKRE in Q2 ranging from 23.6 [95% CI: 21.8–25.5] to 32.2 [95% CI: 29.1–35.4], this suggests that its professional depth was somewhat lower than the other models.****”***

**Comment 6:** Address potential model bias and temporal variability (responses may change across sessions or updates).

**Response:** Thank you for this important suggestion. Please see response to Comment 1.

**Comment 7:** Strengthen the practical implication section by translating findings into concrete guidance for non-specialist educators (e.g., recommended prompt templates or readability calibration strategies):

**Response:** Thank you for this helpful suggestion. We have revised to provide more practical guidance for non-specialist educators and parents who may use LLMs to obtain ADHD related information. Specifically, we added examples of prompting strategies, such as requesting explanations in plain language, using bullet-point summaries, and asking for classroom-based examples. These additions translate our findings into concrete recommendations that may help improve the accessibility and usability of LLM generated health information.

**Revision in manuscript:** The Implications and Future Research section has been expanded (Lines 337-344):

***“****From a practical perspective, non-specialist educators and parents using LLMs to obtain ADHD-related information may benefit from simple prompting strategies to improve clarity and accessibility. For instance, prompts that explicitly request explanations in plain language may help reduce linguistic complexity. Users may also ask the model to provide short summaries or classroom-based examples, which can translate clinical descriptions into more practical educational guidance. Such prompt adjustments may help bridge the gap between technical health information and the needs of non-specialist audiences.****”***

**Comment 8****:** Justify the choice of readability metrics and discuss their known limitations for health education materials.

**Response:** We appreciate this important comment. The manuscript now includes a clearer justification for selecting the Flesch–Kincaid Reading Ease, Flesch–Kincaid Grade Level, and SMOG indices. We also added a discussion acknowledging the limitations of readability formulas when applied to health-education materials.

**Revision in manuscript:** Additional explanations have been added in the Methods and Discussion Strengths and limitations sections (Lines 143-147, 374-389).

***“****The readability of the responses was assessed using three well-established metrics: the FKRE, the FKGL, and the SMOG [8, 9]. The FKRE assigns a numerical score ranging from 0 to 100, with higher values indicating simpler and more accessible text. A score near 100 suggests that the content is very easy to understand, whereas lower scores denote increased complexity.****”***

*“Another limitation relates to potential model bias and temporal variability in large language model outputs. Because LLMs are trained on large and evolving datasets, their responses may reflect biases present in training data or system design. In addition, responses generated by these models may vary across sessions or change over time as the underlying models are updated by developers. Although this study attempted to minimize contextual carryover by using separate sessions and standardized prompts, the findings should be interpreted as a snapshot of model performance during the study period rather than a fixed representation of model capabilities. Another limitation concerns the use of traditional readability metrics to assess the accessibility of AI-generated health information. Indices such as the FKRE, FKGL, and SMOG primarily measure surface linguistic characteristics, including sentence length and word complexity. While these indicators are widely used in health communication research, they may not fully capture conceptual difficulty, contextual interpretation, or the actual comprehension of health information by readers. Therefore, readability scores should be interpreted as approximate indicators of textual complexity rather than definitive measures of user understanding.”*

***Reference：***

1.	Weiss BD. Health Literacy and Patient Safety: Help Patients Understand. Manual for Clinicians. 2nd ed. Chicago, IL: American Medical Association Foundation; 2007.

2.	Villa-de Gregorio M, Palomo-Nieto M, Gómez-Ruano MÁ, Ruiz-Pérez LM. Attentional neurodiversity in physical education lessons: a sustainable and inclusive challenge for teachers. Sustainability. 2023;15(6):5603. doi: <https://doi.org/10.3390/su15065603>.

3.	American Psychiatric Association. What is ADHD? [Internet]. Washington, DC: American Psychiatric Association; 2022 [cited 2025 Aug 8]. Available from: <https://www.psychiatry.org/patients-families/adhd/what-is-adhd>.

4.	American Psychiatric Association. Diagnostic and Statistical Manual of Mental Disorders. 5th ed. Arlington, VA: American Psychiatric Publishing 2013.

5.	Sperka L, Enright E. The outsourcing of health and physical education: A scoping review. European Physical Education Review. 2018;24(3):349-71.

6.	Vázquez-Madrigal C, García-Rubio N, Triguero Á. Generative Artificial Intelligence in Education: Risks and Opportunities. Teaching innovations in Economics: Towards a sustainable world: Springer; 2024. p. 233-54.

7.	Kumar AK, Assaf MH, Groza VZ, Petriu EM, editors. Spirometer and sEMG respiratory patterns for clinical decision support system. 2023 IEEE International Instrumentation and Measurement Technology Conference (I2MTC); 2023: IEEE.

8.	Flesch R. A new readability yardstick. Journal of applied psychology. 1948;32(3):221.

9.	Mc Laughlin GH. SMOG grading-a new readability formula. Journal of reading. 1969;12(8):639-46.
